# High-density genetic linkage map construction and identification of fruit-related QTLs in pear using SNP and SSR markers

**DOI:** 10.1093/jxb/eru311

**Published:** 2014-08-16

**Authors:** Jun Wu, Lei-Ting Li, Meng Li, M. Awais Khan, Xiu-Gen Li, Hui Chen, Hao Yin, Shao-Ling Zhang

**Affiliations:** ^1^Center of Pear Engineering Technology Research, Nanjing Agricultural University, Nanjing 210095, China; ^2^International Potato Center (CIP), Apartado 1558, Lima 12, Peru; ^3^Zhengzhou Fruit Research Institute, Zhengzhou 450009, China

**Keywords:** Genetic linkage map, pear, QTL, RADseq, SNP, SSR.

## Abstract

Pear (*Pyrus* spp) is an important fruit crop, grown in all temperate regions of the world, with global production ranked after grape and apples among deciduous tree crops. A high-density linkage map is a valuable tool for fine mapping quantitative trait loci (QTL) and map-based gene cloning. In this study, we firstly constructed a high-density linkage map of pear using SNPs integrated with SSRs, developed by the rapid and robust technology of restriction-associated DNA sequencing (RADseq). The linkage map consists of 3143 SNP markers and 98 SSRs, 3241 markers in total, spanning 2243.4 cM, with an average marker distance of 0.70 cM. Anchoring SSRs were able to anchor seventeen linkage groups to their corresponding chromosomes. Based on this high-density integrated pear linkage map and two years of fruit phenotyping, a total of 32 potential QTLs for 11 traits, including length of pedicel (LFP), single fruit weight (SFW), soluble solid content (SSC), transverse diameter (TD), vertical diameter (VD), calyx status (CS), flesh colour (FC), juice content (JC), number of seeds (NS), skin colour (SC), and skin smooth (SS), were identified and positioned on the genetic map. Among them, some important fruit-related traits have for the first time been identified, such as calyx status, length of pedicel, and flesh colour, and reliable localization of QTLs were verified repeatable. This high-density linkage map of pear is a worthy reference for mapping important fruit traits, QTL identification, and comparison and combination of different genetic maps.

## Summary

A highly saturated pear genetic map was constructed using over 3000 SNP markers developed by RADseq integrated with anchored SSR markers. The reliable QTLs of several fruit traits were also identified.

## Introduction

Pear (*Pyrus* spp.) is one of the most important and popular fruits in Europe, East Asia, and North America, with a cultivation history of up to 3000 years (Lombard and Westwood, 1987). The production of pear in the world was 23.9 million tones and the harvested area was 1.6 million hectares, ranking it as the second most important fruit tree in Maloideae after apple (http://faostat.fao.org). However, as pear is perennial with a long juvenile period, it is not easy for growers and breeders to directly determine genes controlling target traits. Thus, revealing the location of trait-control genes on linkage groups and obtaining applicable markers for marker-assisted selection by the construction of genetic linkage maps and agronomic trait mapping is of significant value.

A genetic linkage map is the arrangement of genetic markers on the chromosome as inferred by segregation data of genetic material in a population resulting from a specific cross (Collard *et al.*, 2005). It has been widely applied to many fruit trees for molecular genetics and breeding, especially to map QTLs for fruit quality traits and disease-resistance genes (Longhi *et al.*, 2014). For example, recently, fruit quality QTLs for colour, fruit form, and soluble solid content in apricot were detected based on linkage mapping (Socquet-Juglard *et al.*, 2013). In apple, genetic and physical characterization was investigated to fine map the columnar locus (Baldi *et al.*, 2013). Several fruit quality traits, including fruit circumference, diameter at midpoint, length, weight, total soluble solids, and total titratable acids, were newly identified in apple (Potts *et al.*, 2014). In the past dozen years, definite progress had been made in the development of genetic markers and construction of linkage maps in pear. The first pear map was of the Japanese pear cultivars ‘Kinchaku’ and ‘Kosui’, reported with 120 RAPD markers, and consisting of 18 linkage groups, spanning 768 cM (Iketani *et al.*, 2001). The following published pear linkage maps used either AFLP or SSR markers, with marker number of ranging from 154–447 (Yamamoto *et al.*, 2002*c*; Dondini *et al.*, 2005; Yamamoto *et al.*, 2007; Terakami *et al.*, 2009). This low marker density, along with the time and labour required, and the high costs of the marker type makes most of them unsuitable for fine mapping of traits of interest and for full use for breeding purposes. Furthermore, most SSRs previously published in pear genetic maps were derived from other species. However, the whole genome sequence of pear (*Pyrus bretscheideri* Rehd) was recently released (Wu *et al.*, 2013), making it possible to massively develop genetic markers of pear directly (Fan *et al.*, 2013). With the development of next generation sequencing technologies, a set of 1096 SNPs (single nucleotide polymorphism) identified from three European pear cultivars and 7692 apple SNPs in Infinium^®^ II 8K array were combined together to develop useful markers for genotyping in *Pyrus*. An SNP-based map was first tried with 857 polymorphic markers (Montanari *et al.*, 2013); however, this is still not dense and saturated enough to be a desirable and fine map for QTL localization.

Many kinds of markers have been developed for genetic map construction, including non-PCR based markers, RFLP (restriction fragment length polymorphism), arbitrarily primed PCR based markers, RAPD (random amplified polymorphic DNA), AFLP (amplified fragment length polymorphism), sequence-specific PCR-based markers, SSRs (single sequence repeats), and SNP (single nucleotide polymorphism) (Agarwal *et al.*, 2008). Among these, SSRs are the most widely applied for their conservation, synteny, and superior transferability (Marques *et al.*, 2002; Gasic *et al.*, 2009). However, the limited number of developed markers still makes high-density mapping difficult. Recently, along with the spread of next-generation sequencing (NGS) technology, the approach for understanding the underlying molecular genetic mechanism of fruit trees has promoted the development of SNP markers, such as grape (Wang *et al.*, 2012), citrus (Fujii *et al.*, 2013), and peach (Martiínez-Garciía *et al.*, 2013). The method for obtaining SNP markers includes commercial genotyping assays in peach, customized genotyping array in citrus, and restriction-site-associated DNA sequencing (RADseq) in grape. Compared with others, the RADseq approach, combining basic molecular techniques with NGS (Davey and Blaxter, 2010), is rapid, inexpensive, and species-independent, and so we chose it for conducting the current study. Meanwhile, the application of RADseq had been reported for many species besides grape, such as eggplant (Barchi *et al.*, 2011), barley (Chutimanitsakun *et al.*, 2011), and *Lolium perenne* (Pfender *et al.*, 2011), but not yet for pear. Therefore, construction of new genetic maps utilizing RADseq was needed in pear.

In pear, a SNP marker-based pear genetic map was constructed for anchoring scaffolds (Wu *et al.*, 2013). Furthermore, to fully exploit genetic maps for comparative genomics research and QTL fine mapping of important agronomic traits, we constructed a high-density genetic map integrating SNP and SSR markers, and analysed QTLs of fruit quality related traits using these markers.

## Materials and methods

### Plant material and DNA isolation

An F1 pear population of 102 individuals from a cross between ‘Bayuehong’ and ‘Dangshansuli’ was used for mapping. ‘Bayuehong’ is a descendant of European pear ‘Clapp’s Favorite’ (*Pyrus communis* L.) and the Chinese pear ‘Zaosuli’ (*Pyrus bretschneideri* Rehd.). ‘Dangshansuli’ is a native Chinese pear cultivar (*Pyrus bretschneideri* Rehd.). The population was hybridized in the year 2000, and most individuals first fruited in the year 2005 or 2006. The plants were grown in the Fruit Tree Institute of Zhengzhou in Henan Province, China. Young leaves, specifically the first few leaves of apex during the beginning of the vegetative period of each individual along with the parents were collected for DNA isolation. Collected samples were first sent to the lab in liquid nitrogen and transferred to –80 **°**C freezer. ~0.5g of each sample was ground in liquid nitrogen and genomic DNA was isolated using the plant genomics DNA kit (TIANGEN, Beijing, China) following the manufacturer’s protocol. The quality of DNA was critical and DNA concentration of each sample was required to be the same for the construction of sequencing libraries (Chutimanitsakun *et al.*, 2011). RNase was applied for the degradation of possible RNA in the isolated DNA samples, and NanoDrop 2000 was used for DNA concentration determination.

### RAD sequencing analysis

The construction of RAD libraries was similar to a set of previously published papers for RAD marker development using next-generation sequencing technology (Chutimanitsakun *et al.*, 2011; Pfender *et al.*, 2011). Briefly, seven steps were applied. First, 500ng purified genomic DNA of each sample was digested by 20 units endonuclease restriction enzyme *Eco*RI (New England BioLabs, NEB), for 60min at 37 °C in a 30 µl reaction. The product was heat inactivated for 20min at 65 **°**C. Second, the 30 µl digestion products were ligated with 2.0 µl of 100nM P1 adapter by 1.0 µl T4 DNA ligase (1000U µl^–1^, NEB), along with 1 µl of 10mM rATP, 1 µl 10X NEB Buffer, 5 µl nuclease-free water and incubated at room temperature for 20min. The P1 adapter was with the molecular identifier and sticky-end matching the *Eco*RI cleavage site (top sequence: 5′-GATCTA CACTCTTTCCCT ACACGACGCT CTTCCGATCTxxxxxx-3′, bottom sequence: 5′-phos-AAT Txxxx xxAGAT CGGAAGAGCGTCGTGTAGGGAAAGAGTGTAGATC-3′, ‘xxxxxx’ means molecular identifier). Third, a batch of samples was pooled together and randomly sheared ultrasonically (Covaris S2 (Covaris Inc.)). The average length of sheared fragments was confined to 500bp. QIAquick PCR Purification kit was applied for purifying sheared DNA fragments. Fourth, purified DNA was loaded on a 1.25% agarose gel with 100bp DNA ladder and 350–500bp DNA bands were cut from the gel and purified with MiniElute Gel Purification Kit (Qiagen). Fifth, the fragment end was repaired with Quick Blunting kit (NEB). A 3′-dA overhang was added using dA-tailing module (NEB). Then P2 adapter (Top: 5′-T GATCGG AAGAGCACAC GTCTGAACTCCAGTC ACCTTGTAAT CAGAACAA-3′, bottom: 5′-CAAGCAG AAGACGGCAT ACGAGATTACAAG GTGACTG GAGTTCAGACGTGTGCT-CTTCCGATC-3′) was ligated to the sticky end with an overhanging A. Sixth, the collected fragments were enriched by PCR amplification (Forward primer: 5′-AATGATA CGGCGACCAC CGAGATCTACA CTCTT TCCCT ACACG ACGCTCTTCCGATCT-3′, reverse primer: 5′-CAAGCAGA AGACGGC ATAC GA-3′) and purified by QIAquick PCR purification kit. Finally, each sample was normalized to 10nM for sequencing in Illumina HiSeq2000 following the manufacturer’s protocol.

### SNP identification and genotyping

SNP identification and genotype scoring were performed using Stacks package (Catchen *et al.*, 2013) with customized Perl scripts. Briefly, reads with low quality were discarded, and original reads were identified by their barcodes. Then, reads of each individual were clustered, aligned with each other and scored for SNPs. The marker code was based on the software Joinmap 4.0 and the population type CP (cross pollinators). CP is a cross between two heterozygous diploid parents, with linkage phases originally unknown (Van Ooijen, 2006). There are five segregation types of CP population (lmxll, nnxnp, hkxhk, efxeg, abxcd), but only three segregation types were genotyped here. The marker code ‘lmxll’ represents markers with first parent heterozygous and second parent homologous, ‘nnxnp’ represents markers with first parent homologous and second parent heterozygous, and ‘hkxhk’ represents markers with both parents heterozygous. The putative loci were filtered to remove erroneous data. Valid loci for genetic mapping were filtered by the following criteria. First, the expected segregation ratio for ‘lmxll’ and ‘nnxnp’ was 1:1, and including ‘hkxhk’, it was 1:2:1. Chi-square was tested and the threshold *P*-value was set to 0.05. Second, the sequencing depth of each locus of each plant was checked, and the lower depth genotype was set as missing, as it can be caused by sequencing or other errors. Third, any locus with more than 10 missing data was filtered, with the view that too much missing data would influence the mapping result.

### SSR genotyping

Sequence information of SSR primers were obtained from HiDRAS (http://www.hidras.unimi.it/) and several published papers (Liebhard *et al.*, 2002; Yamamoto *et al.*, 2002*a*, 2002*b*, 2002*c*; Sawamura *et al.*, 2004; Fernández-Fernández *et al.*, 2006; Inoue *et al.*, 2007; Celton *et al.*, 2009; Nishitani *et al.*, 2009). The segregation data for SSR markers in the same population were detected and 112 of them were used for constructing an integrated map. Microsatellites were PCR amplified in a PTC-200 Thermo Cycler (BIO-RAD) using the reaction system under the following conditions: the reaction mixture was 20 µl, and contained 2.0 µl of 10X reaction buffer (100mM Tris-HCl, pH 8.3), 50mM KCl, 1.6mM MgCl_2_, 0.25mM of each dNTP, 0.2 µM of each primer, 1.0U Taq polymerase (TaKaRa), and 25–50ng of genomic DNA. The PCR reactions were performed with the following conditions: 94 °C for 3min, then 39 cycles of annealing (55 °C for 50 s), extension (72 °C for 1min), and final cycle of extension (72 °C for 10min). The products of PCR amplification were analysed by 8% polyacrylamide gel electrophoresis. Band scoring was performed using a standard 100bp ladder and pBR322 DNA/MapI as control.

### Phenotyping fruit-related traits

The fruit traits at ripening stage were investigated in 2008 and 2009. Ten random fruits were picked from each tree and eleven major traits for fruit were measured. These traits were: length of pedicel (LFP), single fruit weight (SFW), soluble solid content (SSC), transverse diameter (TD), vertical diameter (VD), calyx status (CS), flesh colour (FC), juice content (JC), number of seeds (NS), skin colour (SC), and skin smooth (SS). LFP, TD, VD were measured using vernier calipers. SFW was measured using electronic scales. SSC was measured using a refractometer (Atago, model N-1). CS had three possible statuses, calyx-remain, calyx-remnant, and calyx-abscission. FC had five possible statuses, white, milky white, light green, light yellow, and yellow. JC was estimated to six levels from extreme little to masses. NS was observed as two levels, 10 seeds and less than 10 seeds. SC of fruit was observed as either red or green. SS was observed as rough, medial, or smooth. According to the measurement type and trait characterization, all 11 fruit traits were divided into qualitative trait group and quantitative trait group, with CS, FC, JC, SC, NS, and SS considered as qualitative traits and LFP, SFW, SSC, TD, and VD considered as quantitative traits.

### Map construction and QTL analysis

Joinmap 4.0 (Van Ooijen, 2006) was used for linkage map construction. Scored SNP and SSR markers were first mixed together with all kinds of marker types and loaded into Joinmap 4.0. The segregation rate of markers was first tested in Joinmap 4.0 and distorted markers (chi-square threshold of 0.01) were left out from further analysis. Regression mapping as the mapping algorithm, Kosambi as the mapping function, and LOD (logarithm of odds) of 9.0 as the minimum LOD score were used to establish linkage groups. After grouping, linkage group number was named after the corresponding chromosome number of the SSRs in it. MapChart (Voorrips, 2002) was used to make linkage group figures.

MapQTL5.0 (Van Ooijen, 2004) was used to conduct QTL analysis. The Kruskal–Wallis (KW) test, a non-parametric method was used to detect QTLs for qualitative data. Markers with a *P* value<0.01 by KW test were declared as candidate QTLs. Interval Mapping (IM) was used to initially detect QTLs in quantitative data and nearby loci with the highest LOD scores were selected as co-factors. Markers associated at *P*<0.02 after automatic cofactor selection were then used for multiple QTL model (MQM) computation. The LOD threshold of 2.5 (Khan *et al.*, 2013; Zhang *et al.*, 2013) was used to identify potential QTLs. A significant LOD threshold was calculated by permutation test as 3.5 at the 95% confidence level.

## Results

### RAD tag sequencing and SNP discovery

A total of 1.18 billion raw single-end reads of 49bp were obtained, yielding a total length of more than 58.0 Gb. After identifying barcodes in the start of each read and trimming raw data into the same read length of 41bp, the total number of reads was reduced to 1.12 billion, and valid sequence decreased to 45.8 Gb. The number of reads for each individual affected the capability of SNP calling. The results showed that reads per individual were not equal in the population, with a maximum of 37.1 million, and minimum of 0.4 million (Supplemental Table S1 available at *JXB* online). Among these, the sequencing depth of parents had more impact than progenies on SNP scoring, as they were the basis for genotyping for each locus. The maternal ‘Bayuehong’ obtained 24.0 million reads, a rate of 2.1%, and paternal ‘Dangshansuli’ obtained 15.5 million reads, a rate of 1.4%; both proportions were higher than average (Supplemental Table S1).

For calling SNPs, RAD reads of each individual were first aligned with each other and clustered to 93.8 million RAD tags. After disregarding unclustered data (depth<3), the total number of filtered RAD tags remained at 41.5 million. The sequencing depth of each individual indicated the liability of each locus for SNP calling, which varied from a minimum of 4.8X to the maximum of 47.0X, with an average estimated RAD tag depth of 23.9X (Supplemental Table S1). These data were then used for analysing SNPs and cataloguing polymorphism loci. 40 064 putative loci were obtained, consisting of 26 672 lmxll markers (66.6%), 9895 nnxnp markers (24.7%), and 3497 hkxhk markers (8.7%). Finally, 10 861 SNP loci passed the threshold described in the materials and methods.

### Linkage map construction

**Fig. 1.**
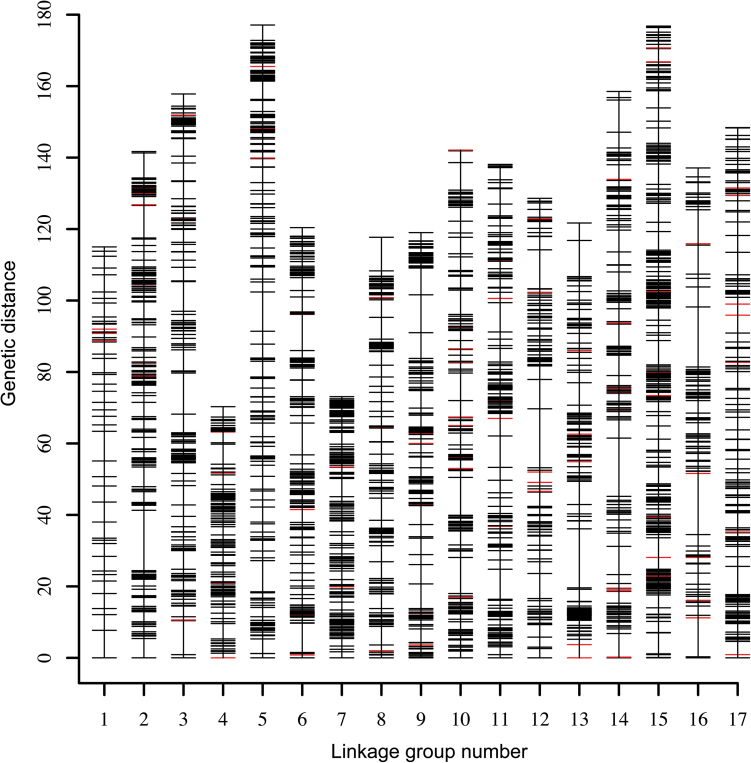
Distribution of SNP and SSR markers on 17 Linkage groups. A black bar indicates a SNP marker, and a red bar indicates an SSR marker. Linkage group number is shown on the x-axis and genetic distance is shown on the y-axis (centiMorgan as unit). (This figure is available in colour at *JXB* online.)

10 861 SNPs and 112 SSRs were loaded into Joinmap 4.0. After grouping, 17 strongly linked groups were selected, with 7732 SNPs and 98 SSRs. Comparison to the pear genome sequence (Wu *et al.* 2013) showed that 2780 SNPs did not map to the corresponding scaffolds. Then, after excluding 1809 markers with more than 10 missing data, a total of 3143 SNP markers (segregation data were listed in Supplemental Table S3 available at *JXB* online) and 98 SSRs were anchored, so these 3241 anchored markers were used for final map construction. The information of all remaining markers are organized in Supplemental Table S4 (available at *JXB* online), which includes marker names, linkage groups, genetic distances, and physical map location in the pear genome. Each LG consisted of at least one SSR, which was used for locating the corresponding chromosome number for each LG and is convenient for map comparison. LG1 and LG7 consisted of the least, with 3 SSRs, and LG10 consisted of the most, with 11 SSRs. The constructed map is shown as Supplemental Figure S1 (available at *JXB* online) and summary statistics of LGs are shown in Table 1. LG1, consisting of 50 markers spanning 115.0 cM with 2.35 cM per marker, had the fewest markers, and lowest density, indicating a lower rate of heterozygosity in Chr1. LG15, consisting of 373 markers with 0.48 cM per marker, had the most markers and highest density, which might infer a higher rate of heterozygosity in Chr15. Excluding LG1 and LG15, LG density ranged from 0.54 cM per marker (LG11) to 1.27 cM per marker (LG16), the number of markers ranged from 106 (LG4) to 281 (LG5). Genetic length of each LG ranged from 73.1 cM in LG7 to 177.1 cM in LG5. The average number of markers of each LG was 190.6, the average genetic length of each LG was 132.0 cM, and average density was 0.70 cM per marker. Overall, the integrated map consisted of 3241 markers, including 3143 SNPs and 98 SSR markers, and had a total span of 2243.4 cM. The distribution of SNPs and SSRs on LGs is shown on Fig. 1 with different colours. Generally, most SSRs were integrated into LGs, and distributed over different parts of the LG.

**Table 1.** Summary of integrated pear linkage groups

Linkage group	No. of markers	No. of SNPs	No. of SSRs	Length (cM)	Average interval between markers (cM)
1	50	47	3	115.0	2.35
2	243	237	6	141.7	0.59
3	231	227	4	157.8	0.69
4	106	101	5	70.3	0.67
5	281	276	5	177.1	0.63
6	210	205	5	120.4	0.58
7	131	128	3	73.1	0.56
8	167	163	4	117.7	0.71
9	177	172	5	119.0	0.68
10	227	216	11	142.1	0.63
11	257	251	6	138.1	0.54
12	162	157	5	128.6	0.80
13	154	149	5	121.7	0.80
14	179	170	9	158.5	0.89
15	373	363	10	176.8	0.48
16	109	104	5	137.1	1.27
17	184	177	7	148.4	0.81
Total	3241	3143	98	2243.4	0.70

### SNP pattern analysis

**Fig. 2.**
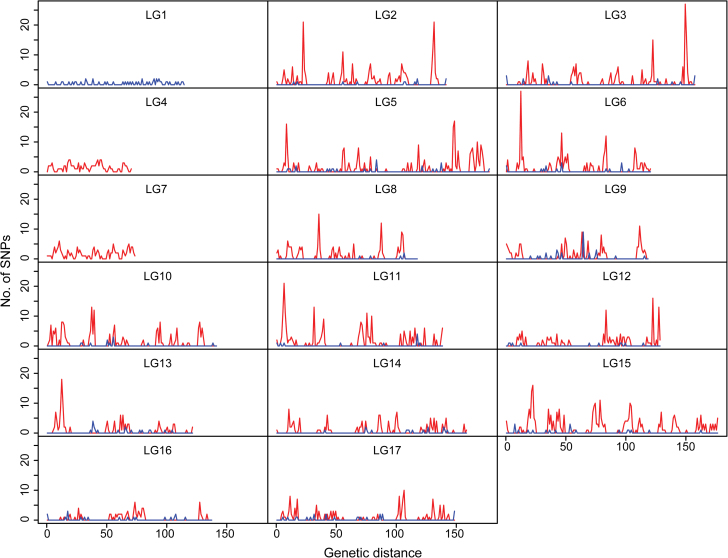
Distribution of number of SNPs on 17 Linkage groups. Red line means SNPs derived from maternal parent ‘Bayuehong’ and blue line means SNPs derived from paternal parent ‘Dangshansuli’. Genetic distance in centiMorgan (cM) is shown on the x-axis and number of SNPs per cM is shown on the y-axis. The longest linkage group on the x-axis is 177.1 cM, and the maximum number of SNPs on the y-axis is 27. Number of SNPs was count with a step-wise of 1 cM window. (This figure is available in colour at *JXB* online.)

**Fig. 3.**
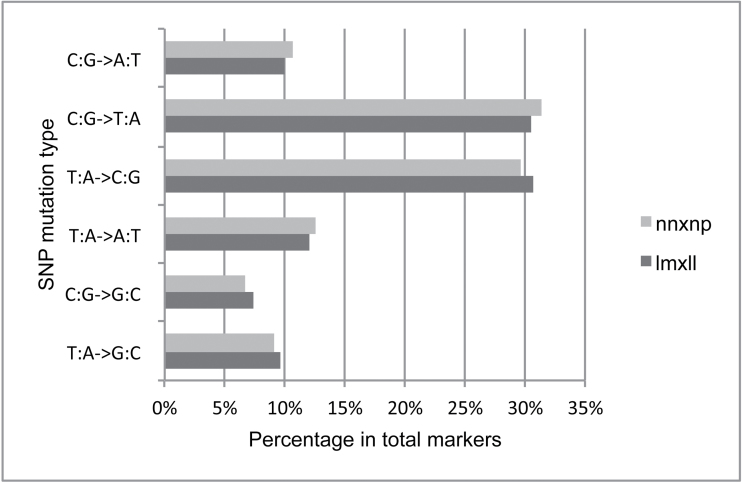
Percentage of each SNP mutation type in total markers.

To investigate the SNP pattern of pear, the distribution of the number of SNPs from each parent on each LG is shown in Fig. 2, and SNP mutation pattern distribution is shown in Fig. 3. Unequal distribution of markers was found on each LG (Fig. 2), with several peaks on most LGs. The highest peak of all LGs with the most markers was on LG3. LG1, LG4, and LG7 had fewer markers and peaks. Furthermore, we found the origin of SNPs from the two parents was also unbalanced. It was found that more segregating SNP markers were from ‘Bayuehong’, with few SNPs originating from ‘Dangshansuli’. 2651 markers were from ‘Bayuehong’, 280 were from ‘Dangshansuli’, and 212 from both, indicating a high rate of heterozygosis of the maternal cultivar. For SNP mutation pattern, we found that the pattern of T (thymine) alternated to C (cytosine) and A (adenine) alternated to G (guanine), or the reverse, of either lmxll marker or nnxnp markers had the largest ratios of pear mutation types, both at least 30% (Fig. 3). Overall, transition, the substitution inside pyridine or purine, was more frequent than transversion, the substitution between pyridine and purine, with the ratio of transition/transversion at 1.56:1 in this study.

### QTL analysis

Eleven fruit yield and quality related traits were selected for QTL analysis. SFW affects fruit production; TD, VD, SC, and SS affect external fruit quality; SSC and JC affect flavour of fruit; CS affects the content of sugar acid and vitamin C (Qi *et al.*, 2013); LFP affects the nutrition and auxin transportation (Drazeta *et al.*, 2004); FC is related to consumer preference (Adami *et al.*, 2013); and NS affects germination and fruit development (Michalak *et al.*, 2013). The eleven measured traits were divided into quantitative and qualitative trait groups. CS, FC, JC, NS, SC, and SS had non-normal data distribution, so we treated them as qualitative traits. QTLs of these traits were detected using the Kruskal–Wallis test with a significance level of 0.01. In total, 18 QTLs were detected in either data of the year 2008 or 2009 (Table 2). Among them, 2 are for CS, 2 for FC, 2 for JC, 6 for NS, 4 for SC, and 2 for SS, of which 5 pairs were confirmed in both 2008 and 2009. These are QTL of CS on LG6, QTL of FC on LG9, QTLs of NS on LG5 and LG17, and QTL of SC on LG16. Furthermore, the location of some QTLs identified in both years were very similar, e.g. QTL of NS localized at 31.2 cM in 2008 and 33.3 cM in 2009 on LG17, QTL of SC localized at 28.8 cM in 2008 and 26.5 cM in 2009 on LG16. The stability of loci for these qualitative traits in different years confirmed the reliability of QTLs.

**Table 2.** Identified QTLs of 11 fruit-related traitsI. QTLs of quantitative traits using interval mapping and multiple QTL model method.

Trait	Year	LG	Marker	Peak Position (cM)	Interval (cM)	LOD	Explained variance (%)
Length of pedicel	2008	14	Pyb14_180	74.9	68.9–85.8	4.20	23.5
	2009	2	Pybd02_013	82.8	76.3–94.7	3.02	14.4
		17	Pyb17_145	45.4	44.9–52.9	3.41	16.0
Single fruit weight	2008	17	Pyd17_012	16.3	11.3–36.4	3.00	18.5
	2009	13	Pyb13_250	99.3	98.9–101.1	2.91	14.6
Soluble solid content	2008	10	Pyb10_134	30.5	30.1–31.6	3.43	30.0
	2009	5	Pyd05_003	10.5	5.3–11.5	2.94	14.2
		14	Pyb14_176	74.0	61.5–85.8	5.18	23.8
Transverse diameter	2008	17	Pyb17_086	15.8	10.2–25.6	3.11	18.5
	2009	3	Pyb03_008	10.4	0–18.9	2.96	14.0
		11	Pyd11_052	117.9	102.3–127	3.07	16.3
Vertical diameter	2008	17	Pyb17_049	11.3	9.6–36.4	4.70	27.7
	2009	11	Pybd11_013	33.6	31.1–35.2	3.32	17.8
		17	Pyb17_292	118.2	106.1–119.2	2.85	19.4
II. QTLs of qualitative traits using Kruskal–Wallis method.							
**Trait**	**Year**	**Group**	**Peak Position (cM)**	**Locus**	**K**	**Significance levels**	
Calyx status	2008	6	101.0	Pybd06_026	11.831	**	
	2009	6	67.8	Pyb06_228	6.925	**	
Flesh colour	2008	9	1.5	Pyb09_012	7.390	**	
	2009	9	13.8	Pyb09_062	6.859	**	
Juice content	2008	1	100.6	Pyd01_080	14.881	***	
	2009	5	11.5	Pyd05_008	16.238	****	
Number of seeds	2008	5	122.1	Pyb05_258	7.671	**	
		14	113.6	Pyd14_051	15.039	***	
		17	31.2	Pyd17_022	7.174	**	
	2009	5	121.6	Pyd05_065	6.981	**	
		9	11.9	Pyb09_046	10.861	***	
		17	33.3	Pyb17_102	9.867	**	
Skin colour	2008	16	28.8	Pyd16_028	8.104	**	
	2009	4	4.8	Pyb04_016	11.349	***	
		13	38.3	Pyd13_006	11.426	***	
		16	26.5	Pyb16_055	12.009	***	
Skin smooth	2008	17	17.1	Pyd17_013	13.062	***	
	2009	2	76.3	Pybd02_010	15.118	***	

K: the Kruskal–Wallis test statistic. **: 0.01; ***: 0.001; ****: 0.0001.

Five traits, LFP, SFW, SSC, TD, and VD, had normally distributed data. Therefore, interval mapping and multiple QTL model with LOD score of 2.5 as potential QTLs were used for QTL detection, and QTLs with LOD score greater than the threshold of 3.5 were considered significant. A total of 14 candidate QTLs were detected in either data of the year 2008 or 2009 (Table 2) and 3 of them were significant QTLs. Among the 14 QTLs, 3 QTLs were detected for LFP, 2 QTLs for SFW, 3 QTLs for SSC, 3 QTLs for TD, and 3 QTLs for VD. Compared with qualitative traits, fewer QTLs for quantitative traits localized repetitively for different years. One pair, VD on LG17, was confirmed in both 2008 and 2009. However, they were not in the same location on the chromosome. QTL of VD on LG17 in 2008 was located at 11.3 cM, and explained 27.7% of variance, whereas QTL in 2009 was located at 118.2 cM and explained 19.4% of variance. Other QTLs could not be confirmed in two years. The explained variance of the 14 QTLs ranged from 13.9% (VD on 99.3 cM of LG13 in 2009) to 30.0% (SSC on 30.5 cM of LG10 in 2008).

### Genetic map comparison by anchoring SSR markers

The constructed integrated map was comprised of 98 SSR markers, listed in Supplemental Table S2 (available at *JXB* online), 63 of which were derived from apple, and 35 from pear. The LG number and map location of each SSR marker was compared with previous pear (Yamamoto *et al.*, 2007; Terakami *et al.*, 2009) and apple (http://www.hidras.unimi.it/) maps, as apple and pear are related species, both in the family of Rosaceae. The five selected genetic maps, ‘Barlett’, ‘La France’, and ‘Hosui’ of pear, and ‘Fiesta’ and ‘Discovery’ of apple were compared through commonly used SSR markers. The results showed that the map generated in this study has 45 common SSR markers with the ‘Barlett’ map, 40 common SSR markers with the ‘La France’ map, 18 common SSR markers with the ‘Hosui’ map, 35 common SSR markers with the ‘Fiesta’ map, and 31 common SSR markers with the ‘Discovery’ map. For each of the 17 linkage groups of pear maps, there was at least one common marker. However, there were no common markers for LG7 and LG13 in the apple ‘Fiesta’ map, and for LG5 and LG7 in the apple ‘Discovery’ map. Overall, 70 of 98 SSR markers used in this study could be located in at least one of the five sets of maps. Most of the 70 common SSR markers have the same order as in the reference map (Supplemental Table S2). However, order and distance disagreement of markers also existed. For example, CH02c02b, CH01d03, and CTG1064355 located at 0 cM, 40.3 cM, and 55.3 cM of LG4 in our map, respectively, while located at 55.3 cM, 19.6 cM, and 55.3 cM, respectively, on LG4 in ‘Barlett’. CH02c02a, NH046a, and CH02f06 were located at the same position, 4.0 cM of LG2, in the map of ‘La France’; while located at close loci 126.8 cM, 130.0 cM, and 131.6 cM, respectively, on LG2 in our map. Furthermore, there were six SNP markers located in between Ch02c02a and NH046a, and 27 SNP markers located in between NH046a and CH02f06.

## Discussion

The construction of RAD libraries reduces the sequencing complexity of the genome by digesting the whole genome to restriction site-associated DNA tags, rather than directly sequencing the whole genome (Davey *et al.*, 2011). It is possible to apply to model or non-model, cultivatable or wild species, with or without reference genomes, and any genome size (Davey and Blaxter, 2010). Nowadays, it has been applied to many species, e.g. barley (Chutimanitsakun *et al.*, 2011), perennial ryegrass (Pfender *et al.*, 2011), and grape (Wang *et al.*, 2012). RADseq, with the capacity of discovering thousands of markers in any organism, is an important technology for ecological population genomics (Davey and Blaxter, 2010). With recent significant advancements, RADseq can also be a tool for rapid high-density genetic map construction and QTL mapping. Besides the current work on pear, previously, it has been applied to grape to construct a high-density genetic map (Wang *et al.*, 2012), perennial ryegrass to identify QTL for stem rust resistance (Pfender *et al.*, 2011), and barley to construct linkage map and conduct QTL analysis (Chutimanitsakun *et al.*, 2011). Compared with the conventional methods for constructing a genetic map of fruit trees, e.g. SRAP, AFLP, and SSR (Zhang *et al.*, 2013), the RADseq-based SNP discovery technique is a worthy new tool for rapidly constructing high-density genetic maps.

The genetic map in this study was the first pear genetic map constructed using over 3000 SNP markers developed from RADseq and integrated with previously published SSRs in different linkage group. It found a highly unbalanced contribution from the two parents (Fig. 2), especially in LG1. This was because of the unbalanced heterozygosity in parent genomes. More SNPs will be developed in a genome with higher ratio of heterozygosity. ‘Bayuehong’ is a descendant of European pear and Asiatic pear, two major cultivar groups, and features a high ratio of heterozygosity. ‘Dangshansuli’ is an ancient cultivar with a cultivated history of more than 500 years in China (Wu *et al.*, 2013), and a subsequently lower level of heterozygosity. Compared with microsatellites, SNPs are considered a better tool for carrying out gene mapping experiments for the reason that they can be genotyped on a much larger scale and are more abundant (Slate *et al.*, 2009). Besides, the power to detect QTL is also enhanced once SNPs are integrated into a first-step SSR-based map with putative QTL locations (Slate *et al.*, 2009). As the pear whole genome sequence has been released (Wu *et al.*, 2013), it can be used in combination with the SNP-based map for fine mapping of genes, as the sequence of each marker is available and genes around each marker are easy to obtain. To test the utility of this approach, we did fruit trait QTL analysis firstly based on SNP map and compared SNP map with previous genetic maps of pear.

Mapping QTLs in pear is challenging, because pear is a self-incompatible plant with high heterozygosity and a long growth and breeding cycle (Wu *et al.*, 2013). Therefore, it is difficult to generate a population that is good for QTL mapping, such as F2 and RIL, and the number of samples for a crossed population is also smaller than annual crops. With the development of sequencing and its application in marker screening, high-resolution linkage maps have been successfully used for QTL fine mapping (Chapman *et al.*, 2012). Previously, a few studies performed mapping and QTL identification using the same F1 population of ‘Dangshansuli’ and ‘Bayuehong’. Han *et al*. (Han *et al.*, 2010) conducted a QTL study for fruit traits using SSR markers, detecting 21 QTLs for the four traits (total soluble solids concentration, mass of single fruit, transverse diameter of fruit, and vertical diameter of fruit), explaining 8.3–33.1% of the variance. In 2011, Zhang *et al.* (2011) identified nine QTLs in the same population using AFLP and SRAP markers for agronomic traits of fruit (total soluble solids concentration, mass of single fruit, transverse diameter of fruit, vertical diameter of fruit, and fruit shape index), explaining 11.4–36.4% of the variance. Another publication (Zhang *et al.*, 2013) presented 19 QTLs identified in the same population, including those for soluble solids content, fruit weight, fruit diameter, fruit shape index, and fruit maturity date, explaining 7.1–22.0% of the variance. The genetic maps these studies constructed were of lower resolution and unsaturated, and possibly unable to ensure the accuracy of identified QTLs. In contrast, markers in the map constructed in this study spanned 2243.4 cM, and had a smaller average marker interval of 0.7 cM, which facilitated localization of QTLs. A total of 32 QTLs were identified for eleven traits, some important pear fruit-related traits have for the first time been identified, such as length of pedicel, calyx status, flesh colour etc, and reliable localization of QTLs were verified repeatable; these corresponding markers are able to be easily located on the whole genome sequence and can be used to screen candidate genes related to traits on those corresponding chromosome regions. Therefore, gene discovery based on this study would be more efficient and rapid. On the other hand, the current study used more observed fruit traits and different QTL analysis methods to detect more putative QTLs and obtain more reliable results based on the higher density genetic map. The KW method was used for qualitative traits with dispersed data and IM for quantitative traits with continuous normal distributed data. The results showed 32 detected QTLs and explained variance of quantitative traits ranging from 14.0–30.0%. Among the QTLs, 12 pairs could be localized on the same LG repetitively in two successive years, but the location of other QTLs changed in different years, a phenomenon also found in previous publications. For example, Kenis *et al.* (2008) detected 74 different QTLs for the major fruit physiological traits of apple. However, only one-third (26) of the identified QTLs were stable over both harvest years, and of these year-stable QTLs only one was a major QTL. This indicates that fruit traits are complex, often involving major and minor effect genes with interaction among genes and environmental factors. Intriguingly, the SSR marker CH04c06 was located on LG17 in our map, but on linkage group 11 in a study by Han *et al.* (2010). Thus, we inferred that they were corresponding linkage groups. In addition, we found that TD and SFW were detected on LG17 in our map and LG11 in the map by Han *et al*., indicating that they might be a common QTL. However, few markers could be verified with previous studies, such as a map using AFLP and SRAP markers (Zhang *et al.*, 2013), owing to deficiency of common markers or anchoring SSR markers for these maps, making it difficult to decipher corresponding linkage groups. The 12 repeatable QTLs that showed stable and significant effects for phenotype of fruit traits are valuable resources for candidate gene exploration in the future, combined with the whole genome sequence of pear (Wu *et al.*, 2013), genes surrounding these QTLs could be listed as candidate genes for further screening and verification, and the corresponding chromosome region could be cloned, and applied to associate analysis for fine gene mapping.

The genetic maps constructed in this study contained a higher number of markers than any previous pear genetic map. Yamamoto *et al.* (2007) constructed integrated reference linkage maps of pear cultivar ‘Bartlett’ and ‘La France’. The map of ‘Bartlett’ consisted of 447 loci, including 58 SSR markers, spanning 1000 cM, with an average distance of 2.3 cM, and the ‘La France’ map consisted of 414 loci, including 66 SSR markers, spanning 1156 cM, with an average marker distance of 2.9 cM. Terakami *et al.* (2009) constructed a genetic linkage map of ‘Hosui’ consisting of 335 loci, including 105 SSR markers, spanning 1,174 cM, with an average marker distance of 3.5 cM. Apple and pear both belong to the sub-family Maloideae in Rosaceae, sharing the same chromosome number, and many SSR markers have been shown to have good transferability between apple and pear (Pierantoni *et al.*, 2004; Gasic *et al.*, 2009). The comparison of our genetic map with previously constructed pear and apple genetic maps showed that all LGs contained common SSR markers between the different maps, and most markers were comparative, with the same order (Supplemental Table S2), with some exceptions. For example, CH02c02b, CH01d03, and CTG1064355 located at 0 cM, 40.3 cM, and 55.3 cM of LG4 in our map, respectively, but were located at 55.3 cM, 19.6 cM, and 55.3 cM, respectively, on LG4 in ‘Bartlett’ (Yamamoto *et al.*, 2007). The reason why CTG1064355 and CH02c02b are located differently might be due to genome structure differences between the different pear cultivars, or their homologous SSR loci. Previously, based on the integrated physical and genetic maps of apple, Han *et al.* (2011) found both genome-wide and segmental duplications present in the apple genome. Khan *et al.* (2012) revealed inconsistent marker order among a multi-population consensus genetic map, which can probably be attributed to structural variations in the apple genome. In addition, CH02c02a, NH046a, and CH02f06 markers were located in the same position in the ‘La France’ map, showing a close relationship at the end of LG2. There were six SNP markers between Ch02c02a and NH046a, and 27 SNPs between NH046a and CH02f06, providing additional information about the locus for future research. If there were genes of interest in these regions, the high density of SNP markers would be very useful for gene screening. In brief, all the commonalities and differences between different maps needs further research, either utilizing whole genome-wide sequence or more SSR markers.

## Conclusion

The study presented here demonstrates that RADseq is a powerful method for genetic marker discovery and genetic map construction for pear. The genetic maps integrated with SNP and SSR markers were high quality and high density, and can be very useful for QTL detection, MAS, map-based cloning, and map comparison.

## Supplementary data

Supplementary data are available at *JXB* online.

Table S1. Statistics of number of reads, clustered tags, filtered tags, and estimation of the depth of filtered tags.

Table S2. Comparison of SSR markers with previously published linkage maps.

Table S3. List of segregation data of 3143 SNP markers.

Table S4. List of markers, linkage groups, genetic distances, and physical map location of SNPs and SSRs in pear.

Figure S1. Figure of ‘Bayuehong’ x ‘Dangshansuli’ genetic map constructed with 3241 markers (3143 SNPs and 98 SSRs).

Supplementary Data
