# Supplementary material for: High-density genetic linkage map construction and identification of fruit-related QTLs in pear using SNP and SSR markers
Source: J Exp Bot. 2014 Aug 16;65(20):5771–81. doi: 10.1093/jxb/eru311 (PMC4203118; doi:10.1093/jxb/eru311)
Supplement: Supplementary Data [file supp_65_20_5771__index.html]

High-density genetic linkage map construction and identification of fruit-related QTLs in pear using SNP and SSR markers — Supplementary Data 

# High-density genetic linkage map construction and identification of fruit-related QTLs in pear using SNP and SSR markers

## Supplementary Data

Data files

**Files in this Data Supplement:**

- Supplementary Data - Supplementary Data
- Supplementary Data - Supplementary Data
