# Supplementary material for: High-density genetic linkage map construction and identification of fruit-related QTLs in pear using SNP and SSR markers
Source: J Exp Bot. 2014 Aug 16;65(20):5771–81. doi: 10.1093/jxb/eru311 (PMC4203118; doi:10.1093/jxb/eru311)
Supplement: Supplementary Data [file supp_eru311_jexbot122010_file001.pdf]

Supplementary data

High-density genetic linkage map construction and identification of fruit-related QTLs in pear using SNP and SSR markers

Jun Wu[1, 4], Lei-Ting Li[1, 4], Meng Li[1], M. Awais Khan[2], Xiu-Gen Li[3], Hui Chen[1], Hao Yin[1], Shao-Ling Zhang[1*]

1 Center of Pear Engineering Technology Research, Nanjing Agricultural University, Nanjing 210095, China

2 International Potato Center (CIP), Apartado 1558, Lima 12, Peru

3 Zhengzhou Fruit Research Institute, Zhengzhou 450009, China

4 These authors contributed equally to this work

* Corresponding author

Shao-Ling Zhang[1*]

Email: slzhang@njau.edu.cn

Telephone: +86 25 84396580

Fax: +86 25 84396485

Supplemental Table S1. Statistics of number of reads, clustered tags, filtered tags, and estimation of the depth of filtered tags (Est. depth).

No. of Individual	Reads	Tags	Filtered tags	Est. depth
P1	23,951,314	1,785,049	502,317	45.1
P2	15,501,781	1,011,547	283,132	8.4
Ind-1	8,038,382	519,929	363,735	21.7
Ind-2	22,257,512	1,666,544	458,100	26.2
Ind-3	14,859,566	683,568	414,604	35.2
Ind-4	6,524,201	924,420	398,408	15.1
Ind-5	7,202,319	490,356	352,187	20.1
Ind-6	5,350,144	1,350,439	541,056	8.4
Ind-7	8,974,000	991,377	406,614	20.6
Ind-8	14,542,369	1,120,753	410,904	17.6
Ind-9	3,504,890	608,010	308,171	10.4
Ind-10	14,522,499	681,114	428,987	33.3
Ind-11	15,188,673	667,166	423,521	35.3
Ind-12	15,830,898	677,229	405,827	38.3
Ind-13	15,850,193	1,142,961	412,519	19.1
Ind-14	14,652,153	643,631	405,003	35.6
Ind-15	16,839,538	666,560	394,840	42.0
Ind-16	17,600,413	1,297,172	432,654	20.6
Ind-17	4,815,317	391,132	286,023	16.5
Ind-18	5,525,028	406,924	298,601	18.1
Ind-19	36,463	8,685	5,705	5.9
Ind-20	8,786,875	505,638	349,254	24.7
Ind-21	13,585,239	1,423,757	468,812	26.9
Ind-22	13,850,778	694,171	433,069	31.4
Ind-23	4,593,320	724,093	356,266	11.9
Ind-24	6,686,767	447,822	327,706	20.0
Ind-25	14,221,257	798,241	434,832	31.9
Ind-26	8,144,125	997,019	418,850	18.1
Ind-27	6,594,786	543,764	358,602	17.9
Ind-28	8,933,617	793,487	347,115	12.2
Ind-29	16,117,799	837,340	410,847	38.2
Ind-30	13,032,946	775,526	409,193	31.0
Ind-31	4,650,154	821,047	356,612	11.7
Ind-32	9,485,728	1,042,006	403,223	21.9
Ind-33	4,921,426	751,025	347,215	13.0
Ind-34	12,202,011	781,593	426,877	27.8
Ind-35	18,422,874	952,474	456,194	39.3
Ind-36	10,905,617	1,171,628	428,593	23.7
Ind-37	4,156,244	422,090	285,493	14.1
Ind-38	10,511,384	654,776	402,593	25.5

Ind-39	10,810,743	1,211,446	426,398	23.5
Ind-40	10,233,784	1,091,472	442,316	21.7
Ind-41	8,247,139	1,031,582	403,099	18.9
Ind-42	7,989,468	626,379	374,778	20.6
Ind-43	15,341,216	853,454	445,399	33.5
Ind-44	19,038,023	985,640	442,809	41.8
Ind-45	4,353,278	733,978	341,061	11.6
Ind-46	11,088,640	1,185,462	505,844	20.6
Ind-47	9,199,110	1,007,067	416,437	20.7
Ind-48	23,478,869	1,117,662	485,868	47.0
Ind-49	8,255,437	546,813	329,605	24.4
Ind-50	10,565,419	1,191,708	425,305	23.0
Ind-51	11,582,911	1,188,271	423,050	25.6
Ind-52	8,252,543	925,464	393,589	19.6
Ind-53	6,966,164	680,882	397,727	16.8
Ind-54	2,917,686	525,735	265,049	10.0
Ind-55	8,529,294	986,101	404,980	19.6
Ind-56	5,588,400	609,609	356,022	15.0
Ind-57	10,323,631	870,621	435,835	22.7
Ind-58	133,997	52,545	19,391	5.2
Ind-59	13,114,301	1,024,898	459,806	27.3
Ind-60	5,258,426	763,812	378,273	12.9
Ind-61	9,970,226	1,072,239	424,206	22.0
Ind-62	10,985,205	1,174,556	448,816	22.9
Ind-63	37,137,876	3,365,480	1,000,309	34.8
Ind-64	13,685,765	1,008,113	456,550	28.8
Ind-65	10,639,579	1,108,918	434,288	22.9
Ind-66	15,456,530	1,340,411	464,490	31.4
Ind-67	9,199,608	794,412	414,284	21.3
Ind-68	4,753,897	574,882	345,566	13.1
Ind-69	9,009,757	993,147	414,647	20.3
Ind-70	11,893,161	906,829	437,956	26.1
Ind-71	20,776,358	1,338,156	496,777	40.1
Ind-72	8,880,482	987,439	415,532	20.0
Ind-73	11,599,724	1,604,344	564,115	18.7
Ind-74	7,918,992	670,632	368,246	20.7
Ind-75	11,264,772	1,218,633	462,132	22.7
Ind-76	7,163,076	952,884	403,055	16.4
Ind-77	10,042,687	1,077,836	427,734	22.0
Ind-78	7,028,212	882,667	394,084	16.6
Ind-79	17,689,383	1,226,365	487,881	34.7
Ind-80	8,624,838	716,947	396,830	20.9
Ind-81	10,381,281	803,830	413,252	24.2
Ind-82	5,048,149	769,523	358,984	12.9

Ind-83	13,172,348	1,230,385	449,725	27.6
Ind-84	9,121,836	1,064,193	466,199	18.3
Ind-85	16,137,868	1,450,902	474,305	32.0
Ind-86	7,664,449	932,445	395,743	18.0
Ind-87	6,106,378	824,062	382,216	14.8
Ind-88	5,967,141	660,675	360,025	15.7
Ind-89	6,017,717	1,017,082	506,158	10.9
Ind-90	8,051,103	937,170	397,578	18.9
Ind-91	9,852,052	630,913	287,994	9.5
Ind-92	6,849,643	470,754	150,790	5.1
Ind-93	4,024,415	485,184	306,421	12.6
Ind-94	11,887,026	872,295	420,339	27.2
Ind-95	20,118,694	1,285,915	395,436	15.2
Ind-96	8,418,444	698,014	407,618	19.9
Ind-97	10,855,839	717,527	382,860	27.5
Ind-98	17,362,214	1,050,982	457,417	36.7
Ind-99	2,225,520	229,822	44,336	4.8
Ind-100	11,152,511	736,696	384,826	28.1
Ind-101	23,832,461	1,318,209	491,795	46.8
Ind-102	5,730,555	558,262	343,977	16.0
**Total**	1,116,336,851	93,842,394	41,462,387	23.9

Supplemental Table S2. Comparison of SSR markers with previously published linkage maps

LG	Map pos. (cM)	Marker name	Yamamoto et al., 2007		Terakami et al., 2009	HiDRAS	
			Barlett	La France	Housui	Fiesta	Discovery
1	88.6	KA4b	59.3		58.7	53.5	42.6
1	90.8	NH010a	69.1				
1	92.0	CH05g08	70.2			69.9	77.4
2	78.9	BGT23b	29.2	36.3	0		
2	82.7	NH033b	22.2				35.2
2	104.7	KU10		11.2			
2	126.8	CH02c02a		4		5.5	6
2	130.0	NH046a	5.6	4			
2	131.6	CH02f06	1.6	4		1.5	3.8
3	10.6	CH03g12	56.1	59.5	103	65.7	61.3
3	58.2	NB113a	30.3	13.4	54.8		
3	123.0	CH03e03				0	3.2
3	151.9	CH03g07				15.7	24.3
4	0.0	CH02c02b	55.3	68		62.7	73.3
4	20.9	CN869475					
4	40.3	CH01d03	19.6	26		24	
4	51.7	CTG1064355	55.3	68			
4	63.2	CN900214					
5	8.3	CTG1064855-2					
5	118.9	TsuENH086			21.5		
5	139.9	EMPc106					
5	148.2	CH04g09	14.8	32.3			
5	165.5	Hi09b04				11.5	
6	0.8	CH03d12	37.7	35		41.8	40.2
6	12.1	U78949x				55.7	66.4
6	41.5	CTG1063987					
6	52.8	CH01b11					
6	96.1	CTG1070694					
7	19.9	TsuENH006			69.8		
7	53.4	EMPc117					
7	58.5	EMPc111					
8	2.0	CH01h10					51.8
8	64.5	CH05a02				28.5	
8	64.7	Hi20b03				27.5	
8	100.8	NH036b	10.6	0	3.5		
9	3.6	CO898678					
9	12.6	CH05a03			38.9		
9	43.1	NB106a	35.3				
9	60.0	Hi04a05				36	54.3
9	62.9	KA20	23.5				

10	13.9	CH02b03b	62.9	69.2	64.3	64.2	82
10	16.9	MS06g03	62.9	76.4	85.4	74	96
10	53.0	CH03d11					
10	56.2	BGT24					
10	65.0	CH02c11	41.8			38.1	39.9
10	67.4	NH045a	40	43.3	33		
10	82.8	CH01f12	37.4	43.3		33.2	34.4
10	83.0	CTG1066085-					
10	86.5	AU223670					
10	92.5	NH039a	30	24.8			
10	142.1	CH02a08	15.6	13.9		24.9	23.2
11	3.5	CH04g07	68.7	67.3	70.6	53.4	72.7
11	36.8	IPPN14					
11	67.0	CH05c02			43.3		
11	72.2	NH005b					
11	100.6	CH04h02	1.6	0	0	0	0
11	111.4	EMPc11					
12	46.6	CH01f02	44.3	36.7		55.4	55.6
12	49.1	NB104a					
12	52.0	CH01d09				44.7	
12	102.3	CH04g04				30.2	29.3
12	122.9	CH05d04	0	5.7		10.8	12.6
13	0.0	CH02e02			17.7		
13	3.7	AU223486					13.9
13	54.9	CTG1060382					
13	62.6	CTG1067935					
13	86.0	NB133a	35.5	53.6			
14	0.2	NH004a	0	11.4	10		
14	18.8	CH05g07	15.5	11.4		31.5	30.9
14	19.5	EMPc108					
14	69.5	CH04f06	32.3	31.2			
14	74.1	NH001c	38.9	38.8			
14	75.5	CH05g11	38.9	40.6			
14	75.7	CH04c07	42.3	40.6			
14	93.7	CH03a03					
14	133.9	CH05d03	45.3	46.3			
15	22.9	NH027a	0	0			
15	28.1	CH02d10b					
15	39.4	CH03b06				10.7	
15	73.4	CTG1063001					
15	74.5	IPPN08					
15	74.6	CH01d08	9.1	24.8		35.1	36.7
15	80.0	NB129a	10.3	34.9			
15	102.5	EMPc104					

15	166.8	CH02c09	68.4	77.1	118.7	104.1	110.3
15	170.7	NB102a			118.7		
16	11.2	CN910353					
16	16.1	CH02d10a				0	0
16	28.1	AU301431				24.8	
16	51.6	CTG1062447					
16	115.9	NB123a	52.3				
17	0.9	NH014a	42	62.2			
17	35.2	NH008b					
17	82.7	CH01h01	11	26.9		15	24.5
17	95.9	CH05g03		20.6		13.2	21.2
17	99.0	NB110a	5.4	11.3			
17	129.4	AT000174					
17	131.5	CH04c06	4.2	3.8		2.8	0

Supplemental Table S3 List of segregation data of 3,143 SNP markers
See the supplemental Excel file

Supplemental Table S4. List of markers, linkage groups, genetic distances, and physical map location of SNPs and SSRs in pear

Marker	Type	LG	Map pos.	Anchoring Scaffold	Physical position
Pyd01_001	SNP	1	0.0	scaffold87.0	317,382
Pyd01_002	SNP	1	7.7	scaffold947.0	126,623
Pyd01_005	SNP	1	12.1	scaffold205.0	164,165
Pyd01_006	SNP	1	13.8	scaffold231.0	540,263
Pyd01_009	SNP	1	18.0	scaffold112.0	427,811
Pyd01_012	SNP	1	21.5	scaffold87.0	431,677
Pyd01_013	SNP	1	23.3	scaffold18.0	1,243,240
Pyd01_014	SNP	1	24.3	scaffold18.0	1,361,613
Pyd01_016	SNP	1	28.4	scaffold175.0	385,513
Pyd01_018	SNP	1	32.0	scaffold18.0	1,433,522
Pyd01_019	SNP	1	32.9	scaffold58.0	1,041,241
Pyd01_021	SNP	1	33.5	scaffold18.0	1,478,301
Pyd01_023	SNP	1	38.0	scaffold371.0	360,967
Pyd01_024	SNP	1	38.0	scaffold58.0	56,306
Pyd01_031	SNP	1	43.6	scaffold144.0	716,041
Pyd01_034	SNP	1	48.1	scaffold58.0	96,189
Pyd01_035	SNP	1	50.7	scaffold949.0	97,040
Pyd01_038	SNP	1	55.1	scaffold58.0	805,699
Pyd01_042	SNP	1	63.4	scaffold90.0	821,578
Pyd01_043	SNP	1	65.2	scaffold146.0	453,439
Pyd01_046	SNP	1	67.6	scaffold1672.0	10,025
Pyd01_047	SNP	1	69.4	scaffold1193.0	361
Pyd01_049	SNP	1	71.5	scaffold1193.0	24,980
Pyd01_051	SNP	1	74.6	scaffold276.0	15,149
Pyd01_052	SNP	1	76.6	scaffold90.0	682,469
Pyd01_055	SNP	1	79.3	scaffold496.0	160,111
Pyd01_056	SNP	1	79.8	scaffold146.0	484,055
Pyd01_060	SNP	1	83.8	scaffold326.0	286,908
Pyd01_061	SNP	1	85.0	scaffold276.0	435,476
Pyd01_064	SNP	1	88.4	scaffold276.0	526,459
KA4b	SSR	1	88.6	scaffold16.0	1,113,866-1,113,585
Pyd01_065	SNP	1	89.0	scaffold90.0	434,400
Pyd01_066	SNP	1	89.6	scaffold90.0	692,151
NH010a	SSR	1	90.8	scaffold280.0	176,365-176,222
Pyd01_067	SNP	1	91.1	scaffold95.0	439,232
Pyd01_068	SNP	1	91.2	scaffold95.0	150,973
Pyd01_069	SNP	1	92.0	scaffold90.0	432,488
CH05g08	SSR	1	92.0	scaffold20.0	60,528-60,961
Pyd01_070	SNP	1	93.4	scaffold95.0	613,396
Pyd01_071	SNP	1	93.6	scaffold95.0	607,956

Pyd01_072	SNP	1	94.2	scaffold95.0	59,920
Pyd01_074	SNP	1	95.4	scaffold95.0	802,294
Pyd01_078	SNP	1	99.3	scaffold37.0	658,903
Pyd01_080	SNP	1	100.6	scaffold37.0	608,763
Pyd01_081	SNP	1	102.4	scaffold37.0	665,470
Pyd01_084	SNP	1	107.2	scaffold95.0	79,302
Pyd01_085	SNP	1	109.1	scaffold95.0	795,943
Pyd01_087	SNP	1	112.4	scaffold161.0	685,206
Pyd01_089	SNP	1	113.8	scaffold161.0	383,427
Pyd01_090	SNP	1	115.0	scaffold161.0	356,409
Pyb02_002	SNP	2	0.0	scaffold358.0	294,689
Pyb02_005	SNP	2	5.4	scaffold358.0	386,884
Pyb02_006	SNP	2	5.4	scaffold978.0	105,801
Pyb02_008	SNP	2	6.1	scaffold283.0	18,322
Pyb02_009	SNP	2	6.1	scaffold283.0	3,535
Pyb02_010	SNP	2	6.2	scaffold978.0	22,326
Pyb02_012	SNP	2	6.4	scaffold358.0	220,742
Pyb02_014	SNP	2	6.8	scaffold358.0	326,015
Pyb02_016	SNP	2	7.3	scaffold358.0	386,916
Pyb02_017	SNP	2	7.3	scaffold358.0	311,699
Pybd02_004	SNP	2	8.8	scaffold672.0	8,836
Pyb02_029	SNP	2	9.3	scaffold426.0	266,895
Pyb02_031	SNP	2	9.7	scaffold426.0	274,179
Pyb02_033	SNP	2	10.1	scaffold614.0	55,820
Pyb02_044	SNP	2	12.9	scaffold291.0	176,421
Pyb02_046	SNP	2	13.0	scaffold487.0	233,418
Pyb02_048	SNP	2	13.0	scaffold305.0	167,807
Pyb02_050	SNP	2	13.1	scaffold291.0	177,344
Pyb02_053	SNP	2	13.3	scaffold305.0	291,828
Pyb02_058	SNP	2	13.7	scaffold292.0	433,321
Pyb02_061	SNP	2	13.9	scaffold305.0	504,677
Pyd02_002	SNP	2	14.4	scaffold305.0	326,236
Pyb02_066	SNP	2	16.9	scaffold48.0	128,287
Pyb02_068	SNP	2	17.0	scaffold48.0	160,253
Pyb02_069	SNP	2	17.0	scaffold609.0	66,440
Pyd02_006	SNP	2	17.1	scaffold808.0	163,090
Pyb02_073	SNP	2	17.4	scaffold48.0	107,372
Pyb02_075	SNP	2	19.2	scaffold39.0	220,609
Pyb02_077	SNP	2	20.1	scaffold39.0	784,939
Pyb02_081	SNP	2	22.0	scaffold35.0	920,969
Pyb02_082	SNP	2	22.0	scaffold35.0	1,145,136
Pyb02_086	SNP	2	22.3	scaffold35.0	780,513
Pyb02_087	SNP	2	22.3	scaffold39.0	1,229,250
Pyb02_088	SNP	2	22.4	scaffold196.0	130,466

Pyb02_090	SNP	2	22.4	scaffold35.0	821,814
Pyb02_092	SNP	2	22.4	scaffold196.0	168,810
Pyb02_093	SNP	2	22.5	scaffold35.0	1,145,184
Pyb02_095	SNP	2	22.5	scaffold35.0	1,097,420
Pyb02_096	SNP	2	22.5	scaffold230.0	387,064
Pyb02_097	SNP	2	22.5	scaffold230.0	550,036
Pyb02_098	SNP	2	22.6	scaffold230.0	240,384
Pyb02_100	SNP	2	22.7	scaffold35.0	875,757
Pyb02_101	SNP	2	22.7	scaffold35.0	1,061,743
Pyb02_102	SNP	2	22.7	scaffold39.0	1,289,152
Pyb02_103	SNP	2	22.7	scaffold39.0	1,286,832
Pyb02_104	SNP	2	22.8	scaffold39.0	1,284,413
Pyb02_105	SNP	2	22.9	scaffold35.0	1,224,695
Pyb02_106	SNP	2	22.9	scaffold35.0	1,311,779
Pyb02_107	SNP	2	22.9	scaffold35.0	593,336
Pyb02_108	SNP	2	22.9	scaffold35.0	931,690
Pyb02_109	SNP	2	23.0	scaffold35.0	836,692
Pyb02_112	SNP	2	23.2	scaffold39.0	921,218
Pyb02_113	SNP	2	23.3	scaffold39.0	1,082,848
Pyb02_115	SNP	2	23.3	scaffold196.0	251,310
Pyb02_116	SNP	2	23.4	scaffold230.0	574,234
Pyb02_119	SNP	2	24.0	scaffold35.0	943,766
Pyb02_120	SNP	2	24.1	scaffold230.0	292,290
Pyb02_122	SNP	2	24.4	scaffold35.0	575,439
Pybd02_007	SNP	2	41.3	scaffold1353.0	61,025
Pyb02_123	SNP	2	42.7	scaffold124.0	181,766
Pyb02_124	SNP	2	43.0	scaffold39.0	997,317
Pyb02_125	SNP	2	43.1	scaffold230.0	275,272
Pyb02_126	SNP	2	43.3	scaffold39.0	1,155,308
Pyb02_128	SNP	2	43.5	scaffold124.0	268,871
Pyb02_130	SNP	2	46.5	scaffold196.0	28,386
Pyb02_135	SNP	2	46.9	scaffold39.0	957,389
Pyb02_137	SNP	2	47.0	scaffold39.0	879,667
Pyb02_138	SNP	2	47.1	scaffold39.0	955,123
Pyb02_139	SNP	2	47.2	scaffold39.0	1,107,297
Pyb02_142	SNP	2	47.9	scaffold35.0	1,317,629
Pyb02_145	SNP	2	53.4	scaffold124.0	805,726
Pyb02_146	SNP	2	53.9	scaffold124.0	48,125
Pyb02_147	SNP	2	54.0	scaffold124.0	96,713
Pyb02_150	SNP	2	54.6	scaffold124.0	431,484
Pyb02_151	SNP	2	54.7	scaffold124.0	568,952
Pyb02_154	SNP	2	54.9	scaffold35.0	297,170
Pyb02_155	SNP	2	55.0	scaffold124.0	369,203
Pyb02_156	SNP	2	55.0	scaffold35.0	297,116

Pyb02_157	SNP	2	55.0	scaffold124.0	781,637
Pyb02_160	SNP	2	55.4	scaffold35.0	299,104
Pyb02_162	SNP	2	55.4	scaffold124.0	554,331
Pyb02_163	SNP	2	55.5	scaffold124.0	723,617
Pyb02_164	SNP	2	55.5	scaffold124.0	805,778
Pyb02_165	SNP	2	55.5	scaffold124.0	134,395
Pyb02_166	SNP	2	55.5	scaffold124.0	495,700
Pyd02_012	SNP	2	55.5	scaffold101.0	911,020
Pyb02_167	SNP	2	55.6	scaffold124.0	601,466
Pyb02_168	SNP	2	55.9	scaffold124.0	579,914
Pyb02_170	SNP	2	56.0	scaffold124.0	174,286
Pyb02_171	SNP	2	57.7	scaffold39.0	921,263
Pyb02_174	SNP	2	58.3	scaffold230.0	292,330
Pyb02_175	SNP	2	58.5	scaffold196.0	293,185
Pyb02_182	SNP	2	61.7	scaffold124.0	202,738
Pyb02_185	SNP	2	63.2	scaffold1148.0	42,845
Pyb02_187	SNP	2	63.3	scaffold1148.0	56,913
Pyb02_188	SNP	2	63.5	scaffold247.0	489,604
Pyb02_189	SNP	2	63.5	scaffold48.0	214,999
Pyb02_190	SNP	2	63.5	scaffold609.0	28,944
Pyb02_191	SNP	2	63.5	scaffold609.0	84,116
Pyb02_193	SNP	2	63.6	scaffold1148.0	56,868
Pyb02_194	SNP	2	64.0	scaffold609.0	131,553
Pyb02_195	SNP	2	66.7	scaffold305.0	291,801
Pyb02_196	SNP	2	66.8	scaffold292.0	403,392
Pyd02_016	SNP	2	67.0	scaffold101.0	444,021
Pyb02_197	SNP	2	67.5	scaffold808.0	159,228
Pybd02_008	SNP	2	71.5	scaffold508.0	264,917
Pybd02_009	SNP	2	73.4	scaffold4.0	1,739,394
Pyb02_203	SNP	2	74.2	scaffold447.0	6,059
Pybd02_010	SNP	2	76.3	scaffold283.0	416,246
Pyb02_206	SNP	2	76.6	scaffold283.0	98,469
Pyb02_207	SNP	2	76.9	scaffold508.0	326,136
Pyb02_208	SNP	2	77.0	scaffold508.0	35,005
Pyb02_210	SNP	2	77.3	scaffold283.0	427,223
Pyb02_211	SNP	2	77.3	scaffold508.0	335,064
Pyb02_212	SNP	2	77.4	scaffold101.0	892,655
Pyb02_213	SNP	2	77.4	scaffold508.0	319,468
Pyb02_215	SNP	2	78.2	scaffold101.0	736,390
Pyb02_216	SNP	2	78.2	scaffold101.0	842,245
Pyb02_218	SNP	2	78.3	scaffold101.0	376,174
Pyb02_219	SNP	2	78.4	scaffold101.0	838,081
Pyb02_220	SNP	2	78.7	scaffold101.0	744,745
Pyb02_221	SNP	2	78.7	scaffold101.0	389,264

Pyb02_222	SNP	2	78.7	scaffold101.0	326,622
BGT23b	SSR	2	78.9	scaffold101.0	798,465-798,665
Pyb02_224	SNP	2	79.2	scaffold101.0	436,278
Pyb02_225	SNP	2	79.4	scaffold674.0	111,289
Pyb02_228	SNP	2	80.0	scaffold4.0	1,896,566
Pybd02_011	SNP	2	80.1	scaffold4.0	1,489,478
Pyb02_231	SNP	2	80.4	scaffold4.0	1,754,109
Pyb02_237	SNP	2	81.5	scaffold4.0	1,151,440
Pyb02_238	SNP	2	81.7	scaffold4.0	1,004,233
Pyb02_240	SNP	2	81.9	scaffold4.0	1,012,599
Pyb02_244	SNP	2	82.5	scaffold4.0	796,561
NH033b	SSR	2	82.7	scaffold4.0	797,447-797,667
Pybd02_013	SNP	2	82.8	scaffold4.0	743,475
Pyb02_246	SNP	2	84.1	scaffold4.0	1,742,846
Pyb02_248	SNP	2	85.9	scaffold101.0	460,775
Pyb02_250	SNP	2	86.3	scaffold101.0	437,270
Pyb02_251	SNP	2	86.8	scaffold4.0	1,932,024
Pybd02_014	SNP	2	89.8	scaffold4.0	1,504,434
Pyb02_259	SNP	2	93.2	scaffold349.0	18,528
Pyb02_261	SNP	2	93.8	scaffold4.0	575,199
Pyb02_263	SNP	2	93.9	scaffold50.0	1,148,821
Pyb02_268	SNP	2	94.6	scaffold50.0	997,761
Pyb02_270	SNP	2	94.7	scaffold4.0	642,793
Pyb02_272	SNP	2	94.8	scaffold349.0	230,293
Pyb02_275	SNP	2	94.9	scaffold4.0	575,253
Pyb02_279	SNP	2	99.6	scaffold571.0	5,953
Pyb02_284	SNP	2	99.9	scaffold890.0	64,671
Pyb02_290	SNP	2	100.9	scaffold357.0	268,986
Pyb02_293	SNP	2	101.2	scaffold895.0	95,715
Pyb02_294	SNP	2	101.2	scaffold357.0	391,491
Pyb02_298	SNP	2	102.3	scaffold357.0	269,117
Pyb02_301	SNP	2	102.8	scaffold357.0	188,756
Pyb02_302	SNP	2	102.9	scaffold357.0	171,045
Pybd02_016	SNP	2	103.3	scaffold458.0	366,003
Pyb02_303	SNP	2	103.8	scaffold458.0	46,425
Pyb02_304	SNP	2	104.0	scaffold94.0	557,961
Pyb02_307	SNP	2	104.5	scaffold183.0	132,992
Pyb02_308	SNP	2	104.5	scaffold784.0	46,845
KU10	SSR	2	104.7	scaffold183.0	295,815-296,064
Pyb02_313	SNP	2	104.8	scaffold183.0	570,368
Pyb02_315	SNP	2	104.8	scaffold183.0	357,282
Pyb02_316	SNP	2	104.8	scaffold183.0	548,427
Pyb02_320	SNP	2	104.9	scaffold183.0	614,514
Pyb02_322	SNP	2	105.0	scaffold183.0	566,499

Pyb02_324	SNP	2	105.3	scaffold784.0	26,272
Pyb02_325	SNP	2	105.3	scaffold458.0	260,363
Pyb02_326	SNP	2	105.5	scaffold183.0	197,547
Pyb02_328	SNP	2	105.6	scaffold784.0	119,284
Pyb02_339	SNP	2	106.1	scaffold458.0	182,308
Pyb02_340	SNP	2	106.1	scaffold183.0	373,229
Pyb02_344	SNP	2	106.9	scaffold94.0	691,765
Pyb02_345	SNP	2	106.9	scaffold94.0	602,062
Pyd02_024	SNP	2	106.9	scaffold183.0	536,163
Pyb02_346	SNP	2	107.2	scaffold94.0	610,496
Pyb02_348	SNP	2	107.3	scaffold94.0	507,776
Pyb02_349	SNP	2	107.4	scaffold94.0	847,810
Pyb02_350	SNP	2	107.6	scaffold94.0	661,512
Pyd02_027	SNP	2	107.9	scaffold357.0	129,959
Pyb02_353	SNP	2	108.4	scaffold183.0	664,935
Pyb02_354	SNP	2	108.4	scaffold183.0	602,799
Pyb02_355	SNP	2	108.5	scaffold94.0	782,359
Pyb02_359	SNP	2	109.0	scaffold94.0	697,847
Pyb02_360	SNP	2	109.4	scaffold94.0	646,812
Pybd02_022	SNP	2	111.3	scaffold700.0	180,586
Pybd02_023	SNP	2	113.7	scaffold183.0	320,495
Pyd02_032	SNP	2	115.4	scaffold50.0	991,893
Pyd02_033	SNP	2	117.2	scaffold4.0	1,081,430
Pyd02_034	SNP	2	117.5	scaffold4.0	1,079,441
Pybd02_025	SNP	2	126.6	scaffold209.0	529,373
CH02c02a	SSR	2	126.8	scaffold250.0	102,711-102,861
Pyb02_374	SNP	2	129.1	scaffold113.0	639,031
Pybd02_026	SNP	2	129.5	scaffold453.0	127,797
Pyb02_379	SNP	2	129.6	scaffold113.0	71,388
Pyb02_381	SNP	2	129.7	scaffold453.0	371,054
Pyb02_382	SNP	2	129.8	scaffold453.0	82,882
Pyb02_384	SNP	2	129.9	scaffold140.0	324,428
NH046a	SSR	2	130.0	scaffold453.0	208,104-208,511
Pyb02_387	SNP	2	130.2	scaffold113.0	639,041
Pyb02_388	SNP	2	130.2	scaffold113.0	650,613
Pyb02_390	SNP	2	130.2	scaffold113.0	485,086
Pyb02_391	SNP	2	130.3	scaffold140.0	592,015
Pyb02_392	SNP	2	130.3	scaffold140.0	19,893
Pyb02_393	SNP	2	130.3	scaffold140.0	205,665
Pyb02_395	SNP	2	130.3	scaffold140.0	215,462
Pyb02_397	SNP	2	130.3	scaffold627.0	14,270
Pyb02_402	SNP	2	130.3	scaffold113.0	185,035
Pyb02_403	SNP	2	130.4	scaffold113.0	801,487
Pyb02_404	SNP	2	130.4	scaffold140.0	182,648

Pyb02_407	SNP	2	130.7	scaffold113.0	108,939
Pyb02_413	SNP	2	131.0	scaffold113.0	719,483
Pyb02_414	SNP	2	131.1	scaffold140.0	683,866
Pyb02_417	SNP	2	131.3	scaffold113.0	325,991
Pyb02_419	SNP	2	131.3	scaffold113.0	738,736
Pyb02_420	SNP	2	131.3	scaffold113.0	781,781
Pyb02_423	SNP	2	131.3	scaffold113.0	309,023
Pyb02_426	SNP	2	131.3	scaffold1057.0	23,733
Pyb02_427	SNP	2	131.3	scaffold1057.0	39,503
Pyb02_428	SNP	2	131.3	scaffold140.0	260,309
Pyb02_429	SNP	2	131.3	scaffold113.0	325,323
Pyb02_431	SNP	2	131.4	scaffold140.0	709,262
Pyb02_433	SNP	2	131.4	scaffold113.0	675,882
Pyb02_434	SNP	2	131.4	scaffold113.0	568,424
Pyb02_439	SNP	2	131.4	scaffold113.0	80,723
Pyb02_440	SNP	2	131.5	scaffold453.0	18,191
CH02f06	SSR	2	131.6	scaffold113.0	553,429-553,587
Pyb02_444	SNP	2	131.7	scaffold113.0	553,272
Pyb02_445	SNP	2	131.7	scaffold113.0	612,737
Pyb02_446	SNP	2	131.7	scaffold113.0	843,275
Pyb02_447	SNP	2	131.8	scaffold140.0	169,926
Pyb02_449	SNP	2	131.8	scaffold453.0	50,518
Pyb02_451	SNP	2	131.9	scaffold140.0	686,068
Pyb02_452	SNP	2	132.0	scaffold140.0	745,718
Pyb02_454	SNP	2	132.2	scaffold453.0	5,648
Pyb02_456	SNP	2	132.2	scaffold209.0	195,921
Pyb02_462	SNP	2	132.8	scaffold140.0	345,905
Pyb02_464	SNP	2	133.1	scaffold453.0	365,035
Pyb02_472	SNP	2	133.9	scaffold94.0	438,830
Pyb02_473	SNP	2	134.2	scaffold94.0	270,055
Pyb02_476	SNP	2	134.3	scaffold94.0	371,336
Pyd02_047	SNP	2	141.3	scaffold453.0	91,346
Pyd02_049	SNP	2	141.7	scaffold94.0	376,991
Pyd03_005	SNP	3	0.0	scaffold782.0	182,366
Pyd03_006	SNP	3	0.0	scaffold782.0	29,966
Pyd03_007	SNP	3	0.9	scaffold198.0	304,937
Pyb03_008	SNP	3	10.4	scaffold514.0	226,981
CH03g12	SSR	3	10.6	scaffold344.0	228,663-228,380
Pyb03_009	SNP	3	11.5	scaffold393.0	170,109
Pyd03_013	SNP	3	14.5	scaffold198.0	644,854
Pyd03_014	SNP	3	14.7	scaffold344.0	278,983
Pyb03_017	SNP	3	15.3	scaffold348.0	105,014
Pybd03_003	SNP	3	16.4	scaffold198.0	552,259
Pyb03_020	SNP	3	17.2	scaffold198.0	392,725

Pyb03_021	SNP	3	17.3	scaffold412.0	216,548
Pyb03_022	SNP	3	17.7	scaffold198.0	80,892
Pyb03_028	SNP	3	17.9	scaffold378.0	150,944
Pyb03_032	SNP	3	18.5	scaffold378.0	305,374
Pyb03_036	SNP	3	18.5	scaffold378.0	41,944
Pyb03_037	SNP	3	18.6	scaffold198.0	215,442
Pyb03_039	SNP	3	18.6	scaffold156.0	61,900
Pyb03_041	SNP	3	18.7	scaffold432.0	30,031
Pyb03_042	SNP	3	18.7	scaffold198.0	585,601
Pyb03_045	SNP	3	18.8	scaffold432.0	51,845
Pyb03_047	SNP	3	18.9	scaffold514.0	294,060
Pyb03_050	SNP	3	21.6	scaffold26.0	742,804
Pybd03_004	SNP	3	22.0	scaffold86.0	618,774
Pyb03_053	SNP	3	22.6	scaffold393.0	411,783
Pyb03_054	SNP	3	22.7	scaffold26.0	857,126
Pyb03_055	SNP	3	22.9	scaffold393.0	424,495
Pyb03_056	SNP	3	22.9	scaffold393.0	312,983
Pyb03_058	SNP	3	23.1	scaffold316.0	344,462
Pybd03_005	SNP	3	23.6	scaffold26.0	615,854
Pyb03_061	SNP	3	24.8	scaffold62.0	660,909
Pyb03_065	SNP	3	24.8	scaffold687.0	310
Pyb03_074	SNP	3	30.0	scaffold86.0	5,944
Pyb03_075	SNP	3	30.0	scaffold86.0	329,678
Pyb03_076	SNP	3	30.1	scaffold86.0	563,897
Pyb03_078	SNP	3	30.1	scaffold86.0	676,933
Pyb03_079	SNP	3	30.2	scaffold86.0	790,188
Pyb03_082	SNP	3	30.3	scaffold86.0	834,834
Pyb03_083	SNP	3	30.3	scaffold938.0	100,810
Pybd03_006	SNP	3	30.6	scaffold86.0	87,627
Pyb03_086	SNP	3	31.1	scaffold938.0	151,068
Pyb03_088	SNP	3	31.1	scaffold938.0	119,497
Pyb03_089	SNP	3	31.1	scaffold938.0	102,106
Pyb03_091	SNP	3	31.6	scaffold86.0	428,781
Pyb03_093	SNP	3	33.0	scaffold302.0	367,587
Pyb03_094	SNP	3	33.9	scaffold86.0	210,226
Pyd03_023	SNP	3	35.2	scaffold378.0	342,618
Pyd03_024	SNP	3	35.3	scaffold378.0	167,231
Pyd03_025	SNP	3	35.3	scaffold782.0	13,911
Pyd03_029	SNP	3	36.7	scaffold432.0	112,268
Pyd03_030	SNP	3	39.6	scaffold62.0	643,071
Pyd03_032	SNP	3	42.8	scaffold86.0	602,105
Pybd03_008	SNP	3	48.2	scaffold808.0	14,246
Pybd03_009	SNP	3	49.5	scaffold302.0	169,250
Pybd03_011	SNP	3	51.6	scaffold722.0	61,067

Pyb03_096	SNP	3	54.6	scaffold265.0	465,831
Pyb03_097	SNP	3	54.7	scaffold302.0	211,253
Pyb03_100	SNP	3	54.8	scaffold265.0	107,738
Pyd03_042	SNP	3	54.8	scaffold182.0	557,048
Pyb03_102	SNP	3	55.1	scaffold265.0	182,372
Pyb03_104	SNP	3	55.5	scaffold257.0	121,234
Pyb03_106	SNP	3	55.7	scaffold257.0	283,176
Pyb03_108	SNP	3	55.8	scaffold257.0	244,596
Pyb03_109	SNP	3	56.0	scaffold455.0	288,043
Pyb03_110	SNP	3	56.1	scaffold455.0	201,965
Pyb03_111	SNP	3	56.2	scaffold455.0	185,712
Pyb03_113	SNP	3	56.4	scaffold257.0	7,491
Pyb03_114	SNP	3	56.5	scaffold257.0	281,841
Pyb03_115	SNP	3	56.8	scaffold559.0	120,267
Pyb03_116	SNP	3	57.2	scaffold659.0	81,500
Pyb03_117	SNP	3	57.2	scaffold1128.0	7,855
Pyb03_118	SNP	3	57.8	scaffold1128.0	57,277
Pyb03_121	SNP	3	58.0	scaffold805.0	144,969
Pyb03_124	SNP	3	58.1	scaffold1357.0	24,474
Pyb03_125	SNP	3	58.1	scaffold61.0	41,972
Pyb03_126	SNP	3	58.1	scaffold456.0	343,205
Pyb03_127	SNP	3	58.1	scaffold808.0	15,189
NB113a	SSR	3	58.2	scaffold456.0	31,896-32,050
Pyb03_130	SNP	3	58.2	scaffold5.0	1,846,119
Pyb03_133	SNP	3	58.4	scaffold713.0	167,111
Pyb03_143	SNP	3	60.1	scaffold508.0	97,196
Pyb03_146	SNP	3	60.5	scaffold438.0	351,175
Pyb03_147	SNP	3	60.6	scaffold253.0	88,983
Pyb03_148	SNP	3	61.0	scaffold253.0	91,595
Pyb03_149	SNP	3	61.0	scaffold438.0	374,255
Pyb03_150	SNP	3	61.0	scaffold253.0	120,084
Pyb03_157	SNP	3	61.9	scaffold186.0	603,849
Pybd03_013	SNP	3	62.1	scaffold808.0	47,074
Pyb03_158	SNP	3	62.5	scaffold324.0	187,908
Pyb03_159	SNP	3	62.6	scaffold324.0	185,792
Pyb03_160	SNP	3	62.6	scaffold324.0	251,455
Pyb03_161	SNP	3	62.7	scaffold324.0	208,191
Pyb03_162	SNP	3	63.0	scaffold324.0	289,981
Pyb03_164	SNP	3	68.2	scaffold456.0	133,529
Pyb03_171	SNP	3	79.7	scaffold324.0	197,030
Pyb03_172	SNP	3	80.1	scaffold324.0	258,301
Pyb03_173	SNP	3	80.3	scaffold324.0	197,093
Pyb03_174	SNP	3	86.2	scaffold438.0	356,128
Pyb03_176	SNP	3	86.9	scaffold186.0	625,050

Pyb03_178	SNP	3	87.5	scaffold324.0	326,983
Pyb03_179	SNP	3	87.9	scaffold115.0	859,705
Pyb03_180	SNP	3	87.9	scaffold324.0	130,979
Pyb03_181	SNP	3	87.9	scaffold186.0	588,385
Pybd03_014	SNP	3	89.4	scaffold150.0	704,131
Pyb03_184	SNP	3	90.5	scaffold63.0	538,359
Pyb03_187	SNP	3	91.5	scaffold123.0	488,790
Pyb03_188	SNP	3	91.6	scaffold1201.0	73,115
Pyb03_189	SNP	3	91.6	scaffold123.0	490,536
Pyb03_194	SNP	3	92.2	scaffold115.0	568,974
Pyb03_195	SNP	3	92.2	scaffold123.0	358,624
Pyb03_196	SNP	3	92.3	scaffold123.0	613,889
Pyb03_197	SNP	3	92.5	scaffold123.0	613,828
Pyb03_201	SNP	3	93.2	scaffold89.0	373,564
Pyb03_203	SNP	3	93.3	scaffold89.0	556,375
Pybd03_015	SNP	3	93.4	scaffold428.0	250,320
Pyb03_207	SNP	3	93.5	scaffold52.0	510,224
Pyb03_208	SNP	3	93.5	scaffold123.0	227,389
Pyb03_209	SNP	3	93.5	scaffold428.0	313,749
Pyb03_213	SNP	3	93.9	scaffold89.0	475,262
Pyb03_220	SNP	3	94.4	scaffold150.0	556,602
Pyb03_225	SNP	3	96.1	scaffold287.0	287,795
Pyb03_226	SNP	3	97.0	scaffold485.0	183,105
Pyb03_233	SNP	3	105.3	scaffold216.0	399,637
Pyb03_234	SNP	3	105.5	scaffold89.0	506,628
Pyb03_235	SNP	3	105.5	scaffold89.0	556,409
Pybd03_017	SNP	3	109.3	scaffold428.0	247,948
Pybd03_018	SNP	3	111.3	scaffold89.0	366,640
Pyb03_236	SNP	3	113.6	scaffold115.0	509,992
Pyb03_237	SNP	3	113.6	scaffold123.0	625,152
Pyb03_239	SNP	3	113.7	scaffold123.0	700,931
Pyb03_240	SNP	3	113.7	scaffold123.0	448,674
Pyb03_241	SNP	3	113.7	scaffold123.0	495,606
Pyb03_242	SNP	3	115.7	scaffold1216.0	20,774
Pyb03_244	SNP	3	116.3	scaffold150.0	725,671
Pyb03_245	SNP	3	116.3	scaffold1216.0	22,431
Pyb03_250	SNP	3	119.9	scaffold353.0	345,559
Pyb03_251	SNP	3	120.1	scaffold353.0	264,493
Pyb03_254	SNP	3	121.0	scaffold10.0	292,128
Pyb03_256	SNP	3	121.1	scaffold10.0	253,797
Pyb03_258	SNP	3	121.7	scaffold10.0	497,954
Pyb03_263	SNP	3	122.4	scaffold10.0	1,729,200
Pyb03_264	SNP	3	122.4	scaffold10.0	976,061
Pyb03_265	SNP	3	122.6	scaffold10.0	1,290,177

Pyb03_268	SNP	3	122.7	scaffold10.0	1,199,864
Pyb03_269	SNP	3	122.8	scaffold10.0	547,670
Pyb03_270	SNP	3	122.8	scaffold10.0	1,625,773
Pyb03_271	SNP	3	122.8	scaffold10.0	1,297,847
Pyb03_272	SNP	3	122.8	scaffold10.0	1,450,015
Pyb03_273	SNP	3	122.8	scaffold10.0	1,207,393
Pyb03_274	SNP	3	122.8	scaffold10.0	1,037,583
Pyb03_276	SNP	3	122.8	scaffold10.0	1,098,690
Pyb03_277	SNP	3	122.8	scaffold10.0	1,024,624
Pyb03_278	SNP	3	122.8	scaffold10.0	1,058,352
Pyb03_279	SNP	3	122.8	scaffold10.0	1,604,358
Pyb03_280	SNP	3	122.9	scaffold10.0	1,457,914
CH03e03	SSR	3	123.0	scaffold10.0	1,166,953-1,167,159
Pyb03_281	SNP	3	123.1	scaffold10.0	1,403,090
Pybd03_019	SNP	3	124.4	scaffold89.0	422,201
Pyb03_284	SNP	3	124.5	scaffold98.0	89,837
Pyb03_288	SNP	3	125.2	scaffold10.0	1,181,914
Pyd03_052	SNP	3	126.3	scaffold123.0	155,347
Pyd03_053	SNP	3	126.4	scaffold123.0	65,097
Pyb03_290	SNP	3	128.5	scaffold2.0	2,181,940
Pyb03_294	SNP	3	133.3	scaffold2.0	2,180,491
Pyb03_295	SNP	3	133.5	scaffold353.0	233,967
Pyd03_058	SNP	3	138.5	scaffold63.0	690,186
Pyb03_298	SNP	3	140.4	scaffold2.0	2,252,653
Pyd03_064	SNP	3	145.3	scaffold98.0	268,970
Pyb03_300	SNP	3	145.5	scaffold2.0	1,930,867
Pyb03_312	SNP	3	147.0	scaffold1125.0	23,289
Pyb03_314	SNP	3	147.0	scaffold2.0	1,920,682
Pyb03_320	SNP	3	147.3	scaffold2.0	2,038,805
Pyb03_324	SNP	3	147.4	scaffold2.0	2,223,125
Pyb03_327	SNP	3	147.4	scaffold353.0	360,288
Pyb03_329	SNP	3	147.5	scaffold2.0	2,254,182
Pybd03_020	SNP	3	148.8	scaffold98.0	370,550
Pyb03_336	SNP	3	149.2	scaffold63.0	504,452
Pyb03_339	SNP	3	149.2	scaffold63.0	106,503
Pyb03_340	SNP	3	149.3	scaffold957.0	100,835
Pyb03_344	SNP	3	149.4	scaffold485.0	187,548
Pyb03_345	SNP	3	149.5	scaffold98.0	186,581
Pyb03_346	SNP	3	149.6	scaffold98.0	351,373
Pyb03_351	SNP	3	149.7	scaffold118.0	268,149
Pyb03_353	SNP	3	149.7	scaffold2.0	1,144,090
Pyb03_354	SNP	3	149.7	scaffold118.0	618,488
Pyb03_355	SNP	3	149.7	scaffold2.0	1,574,990
Pyb03_356	SNP	3	149.7	scaffold974.0	35,925

Pyb03_357	SNP	3	149.8	scaffold63.0	714,390
Pyb03_358	SNP	3	149.8	scaffold598.0	187,741
Pyb03_359	SNP	3	149.8	scaffold118.0	180,960
Pyb03_360	SNP	3	149.8	scaffold2.0	1,523,162
Pyb03_361	SNP	3	149.8	scaffold98.0	203,726
Pyb03_362	SNP	3	149.8	scaffold2.0	1,187,064
Pyb03_363	SNP	3	149.8	scaffold2.0	1,633,911
Pyb03_364	SNP	3	149.8	scaffold118.0	182,205
Pyb03_365	SNP	3	149.8	scaffold2.0	1,539,458
Pyb03_366	SNP	3	149.8	scaffold118.0	115,948
Pyb03_372	SNP	3	149.9	scaffold98.0	167,300
Pyb03_373	SNP	3	149.9	scaffold63.0	1,100,664
Pyb03_375	SNP	3	149.9	scaffold98.0	350,357
Pyb03_376	SNP	3	149.9	scaffold63.0	1,131,956
Pyb03_377	SNP	3	149.9	scaffold63.0	795,689
Pyb03_378	SNP	3	149.9	scaffold63.0	993,758
Pyb03_382	SNP	3	150.2	scaffold118.0	317,261
Pyb03_383	SNP	3	150.2	scaffold118.0	287,302
Pyb03_384	SNP	3	150.2	scaffold118.0	374,209
Pyb03_385	SNP	3	150.2	scaffold63.0	989,721
Pyb03_386	SNP	3	150.2	scaffold2.0	1,593,186
Pyb03_387	SNP	3	150.2	scaffold98.0	4,605
Pyb03_388	SNP	3	150.2	scaffold98.0	506,056
Pyb03_389	SNP	3	150.2	scaffold98.0	411,218
Pyb03_391	SNP	3	150.2	scaffold98.0	278,599
Pyb03_392	SNP	3	150.2	scaffold63.0	714,307
Pyb03_393	SNP	3	150.3	scaffold2.0	1,580,498
Pyb03_396	SNP	3	150.3	scaffold63.0	1,027,982
Pyb03_397	SNP	3	150.6	scaffold98.0	217,493
Pyb03_400	SNP	3	150.7	scaffold2.0	1,093,014
Pyb03_401	SNP	3	150.8	scaffold974.0	84,336
Pyb03_402	SNP	3	150.8	scaffold2.0	1,084,799
Pyb03_403	SNP	3	151.0	scaffold63.0	1,100,632
Pyb03_404	SNP	3	151.0	scaffold2.0	1,175,922
Pyb03_405	SNP	3	151.1	scaffold118.0	476,634
Pyb03_410	SNP	3	151.7	scaffold974.0	76,965
CH03g07	SSR	3	151.9	scaffold98.0	54,469-54,693
Pybd03_022	SNP	3	152.5	scaffold118.0	360,446
Pyb03_415	SNP	3	153.6	scaffold150.0	351,887
Pybd03_023	SNP	3	153.8	scaffold98.0	24,350
Pyb03_417	SNP	3	154.4	scaffold150.0	681,424
Pyd03_071	SNP	3	157.8	scaffold98.0	304,762
Pyd03_072	SNP	3	157.8	scaffold98.0	342,933
Pyd03_073	SNP	3	157.8	scaffold98.0	307,123

CH02c02b	SSR	4	0.0	scaffold724.0	58,048-58,158
Pyb04_004	SNP	4	1.3	scaffold475.0	285,885
Pyb04_005	SNP	4	1.8	scaffold475.0	174,134
Pyb04_006	SNP	4	2.0	scaffold475.0	253,491
Pyb04_007	SNP	4	2.5	scaffold475.0	344,896
Pyb04_011	SNP	4	3.3	scaffold475.0	160,744
Pyb04_012	SNP	4	3.4	scaffold94.0	133,588
Pyb04_013	SNP	4	4.0	scaffold724.0	183,796
Pyb04_014	SNP	4	4.2	scaffold222.0	578,700
Pyb04_016	SNP	4	4.8	scaffold1725.0	2,336
Pyb04_017	SNP	4	5.3	scaffold222.0	607,921
Pyb04_026	SNP	4	9.6	scaffold300.0	182,303
Pyb04_027	SNP	4	10.1	scaffold134.1	125,376
Pyb04_029	SNP	4	11.0	scaffold300.0	213,066
Pyb04_034	SNP	4	12.5	scaffold300.0	78,099
Pyb04_037	SNP	4	14.2	scaffold483.0	257,154
Pyb04_038	SNP	4	14.9	scaffold483.0	55,736
Pyb04_039	SNP	4	15.1	scaffold483.0	155,872
Pyb04_041	SNP	4	15.9	scaffold483.0	84,448
Pyb04_042	SNP	4	17.1	scaffold135.0	740,140
Pyb04_043	SNP	4	17.5	scaffold135.0	380,161
Pyb04_044	SNP	4	17.8	scaffold200.0	111,698
Pyb04_045	SNP	4	18.0	scaffold462.0	266,966
Pyb04_046	SNP	4	18.1	scaffold135.0	368,047
Pyb04_047	SNP	4	18.4	scaffold135.0	430,190
Pyb04_048	SNP	4	18.8	scaffold135.0	453,315
Pyb04_049	SNP	4	19.1	scaffold135.0	296,863
Pyb04_051	SNP	4	19.6	scaffold200.0	53,330
Pyb04_052	SNP	4	19.6	scaffold135.0	453,279
Pyb04_053	SNP	4	19.8	scaffold200.0	294,380
Pyb04_054	SNP	4	20.1	scaffold462.0	258,427
Pyb04_055	SNP	4	20.7	scaffold462.0	320,002
CN869475	SSR	4	20.9	scaffold191.0	178,433-178,606
Pyb04_056	SNP	4	21.1	scaffold295.0	179,853
Pyb04_057	SNP	4	21.3	scaffold295.0	195,881
Pyb04_061	SNP	4	22.7	scaffold322.0	241,553
Pyb04_062	SNP	4	22.8	scaffold191.0	342,853
Pyb04_063	SNP	4	23.4	scaffold191.0	55,265
Pyb04_064	SNP	4	24.0	scaffold322.0	252,126
Pyb04_066	SNP	4	25.8	scaffold499.0	242,340
Pyb04_067	SNP	4	26.3	scaffold913.0	81,198
Pyb04_068	SNP	4	26.4	scaffold342.0	402,859
Pyb04_070	SNP	4	26.6	scaffold832.0	129,495
Pyb04_077	SNP	4	28.7	scaffold311.0	305,536

Pyb04_078	SNP	4	28.9	scaffold311.0	407,326
Pyb04_080	SNP	4	29.6	scaffold311.0	134,125
Pyb04_082	SNP	4	30.9	scaffold65.0	453,524
Pyb04_083	SNP	4	31.1	scaffold65.0	484,435
Pyb04_085	SNP	4	31.5	scaffold65.0	432,198
Pyb04_086	SNP	4	31.9	scaffold315.0	122,705
Pyb04_087	SNP	4	32.3	scaffold315.0	194,791
Pyb04_088	SNP	4	32.7	scaffold65.0	239,925
Pyb04_089	SNP	4	33.1	scaffold65.0	212,912
Pyb04_091	SNP	4	34.4	scaffold1165.0	76,857
Pyb04_093	SNP	4	36.7	scaffold534.0	228,825
Pyb04_094	SNP	4	37.3	scaffold28.0	654,108
Pyb04_096	SNP	4	37.9	scaffold290.0	157,630
Pyb04_098	SNP	4	38.3	scaffold290.0	173,707
Pyb04_101	SNP	4	38.9	scaffold1030.0	126,642
Pyb04_102	SNP	4	39.1	scaffold1561.0	913
Pyb04_106	SNP	4	39.7	scaffold207.0	189,653
CH01d03	SSR	4	40.3	scaffold96.0	551,862-552,008
Pyb04_112	SNP	4	40.3	scaffold290.0	132,076
Pyb04_114	SNP	4	40.9	scaffold1025.0	90,606
Pyb04_115	SNP	4	41.2	scaffold1561.0	900
Pyb04_116	SNP	4	41.6	scaffold534.0	228,885
Pyb04_117	SNP	4	41.8	scaffold534.0	102,657
Pyb04_118	SNP	4	42.1	scaffold534.0	137,660
Pyb04_119	SNP	4	42.3	scaffold290.0	272,579
Pyb04_120	SNP	4	42.8	scaffold96.0	371,361
Pyb04_121	SNP	4	42.9	scaffold96.0	303,150
Pyb04_122	SNP	4	43.2	scaffold96.0	303,284
Pyb04_125	SNP	4	43.9	scaffold96.0	329,385
Pyb04_126	SNP	4	44.3	scaffold234.0	104,191
Pyb04_127	SNP	4	44.6	scaffold234.0	256,475
Pyb04_128	SNP	4	44.8	scaffold234.0	484,746
Pyb04_129	SNP	4	44.8	scaffold234.0	229,710
Pyb04_130	SNP	4	45.1	scaffold234.0	9,712
Pyb04_131	SNP	4	45.4	scaffold96.0	553,438
Pyb04_132	SNP	4	45.7	scaffold234.0	101,045
Pyb04_133	SNP	4	45.9	scaffold234.0	517,115
Pyb04_134	SNP	4	46.0	scaffold234.0	391,504
Pyb04_135	SNP	4	46.5	scaffold234.0	316,675
Pyb04_136	SNP	4	47.2	scaffold234.0	101,461
Pyb04_138	SNP	4	48.5	scaffold96.0	265,604
Pyb04_140	SNP	4	51.2	scaffold387.0	152,770
CTG1064355	SSR	4	51.7	scaffold7.0	777,042-777,250
Pyb04_142	SNP	4	52.0	scaffold325.0	206,690

Pyb04_147	SNP	4	52.9	scaffold370.0	331,187
Pyb04_148	SNP	4	53.1	scaffold370.0	442,593
Pyb04_153	SNP	4	54.2	scaffold370.0	161,778
Pyb04_163	SNP	4	59.8	scaffold545.0	151,140
Pyb04_165	SNP	4	60.2	scaffold545.0	21,018
CN900214	SSR	4	63.2	scaffold54.2	387,613-387,889
Pyb04_171	SNP	4	63.4	scaffold244.0	156,589
Pyb04_172	SNP	4	63.6	scaffold244.0	385,830
Pyb04_174	SNP	4	63.9	scaffold244.0	201,896
Pyb04_175	SNP	4	64.0	scaffold244.0	56,623
Pyb04_176	SNP	4	64.2	scaffold244.0	413,906
Pyb04_177	SNP	4	64.4	scaffold244.0	458,470
Pyb04_179	SNP	4	65.0	scaffold54.2	416,218
Pyb04_182	SNP	4	65.7	scaffold54.2	370,900
Pyb04_184	SNP	4	66.4	scaffold297.0	165,553
Pyb04_185	SNP	4	66.7	scaffold297.0	244,039
Pyb04_187	SNP	4	67.4	scaffold297.0	61,776
Pyb04_190	SNP	4	70.3	scaffold297.0	158,511
Pyb05_004	SNP	5	0.0	scaffold299.0	414,509
Pyb05_010	SNP	5	1.2	scaffold139.0	209,450
Pyb05_014	SNP	5	5.3	scaffold539.0	202,802
Pyb05_018	SNP	5	6.2	scaffold409.0	84,043
Pyb05_019	SNP	5	6.3	scaffold523.0	216,470
Pyb05_024	SNP	5	6.4	scaffold409.0	365,487
Pyb05_027	SNP	5	7.2	scaffold171.0	402,504
Pyb05_028	SNP	5	7.7	scaffold147.0	305,391
Pybd05_001	SNP	5	8.0	scaffold171.0	207,965
Pyb05_032	SNP	5	8.1	scaffold147.0	218,350
Pyb05_033	SNP	5	8.1	scaffold147.0	693,033
Pyb05_034	SNP	5	8.1	scaffold147.0	725,588
CTG1064855-2	SSR	5	8.3	scaffold147.0	657,406-657,670
Pyb05_040	SNP	5	8.3	scaffold147.0	362,712
Pyb05_041	SNP	5	8.4	scaffold317.0	244,444
Pyb05_042	SNP	5	8.4	scaffold147.0	207,982
Pyb05_043	SNP	5	8.4	scaffold171.0	11,513
Pyb05_044	SNP	5	8.4	scaffold166.0	179,122
Pyb05_045	SNP	5	8.4	scaffold147.0	50,028
Pyb05_046	SNP	5	8.4	scaffold171.0	121,050
Pyb05_047	SNP	5	8.4	scaffold147.0	482,809
Pyb05_048	SNP	5	8.4	scaffold147.0	471,103
Pyb05_049	SNP	5	8.4	scaffold147.0	634,263
Pyb05_051	SNP	5	8.4	scaffold166.0	281,137
Pyb05_052	SNP	5	8.4	scaffold166.0	128,518
Pyb05_053	SNP	5	8.4	scaffold147.0	487,517

Pyb05_061	SNP	5	9.0	scaffold147.0	58,747
Pyb05_062	SNP	5	9.0	scaffold147.0	487,483
Pyb05_064	SNP	5	9.3	scaffold171.0	450,287
Pyb05_065	SNP	5	9.5	scaffold166.0	177,840
Pyb05_066	SNP	5	9.5	scaffold147.0	776,617
Pyb05_067	SNP	5	9.6	scaffold171.0	176,562
Pyd05_002	SNP	5	9.9	scaffold523.0	70,720
Pyd05_003	SNP	5	10.5	scaffold147.0	212,930
Pyd05_008	SNP	5	11.5	scaffold171.0	123,191
Pyb05_070	SNP	5	14.1	scaffold733.0	133,782
Pyb05_071	SNP	5	14.7	scaffold139.0	236,507
Pyb05_072	SNP	5	14.7	scaffold139.0	644,259
Pyb05_075	SNP	5	15.4	scaffold356.0	160,801
Pyb05_078	SNP	5	16.2	scaffold356.0	264,301
Pyd05_013	SNP	5	16.6	scaffold65.0	1,056,035
Pyd05_014	SNP	5	16.7	scaffold65.0	1,083,005
Pyb05_084	SNP	5	18.2	scaffold388.0	53,353
Pyb05_090	SNP	5	18.4	scaffold807.0	124,554
Pyb05_092	SNP	5	18.5	scaffold243.0	504,276
Pyb05_101	SNP	5	27.2	scaffold925.0	7,164
Pyb05_102	SNP	5	27.2	scaffold45.0	1,077,013
Pyb05_103	SNP	5	28.0	scaffold45.0	794,874
Pybd05_007	SNP	5	29.9	scaffold45.0	1,089,733
Pyb05_104	SNP	5	32.9	scaffold643.0	146,752
Pyb05_105	SNP	5	33.1	scaffold65.0	1,072,107
Pyb05_106	SNP	5	33.3	scaffold643.0	113,659
Pyb05_110	SNP	5	33.9	scaffold966.0	32,317
Pyb05_112	SNP	5	35.2	scaffold925.0	16,542
Pyb05_113	SNP	5	37.5	scaffold312.1	37,807
Pyb05_115	SNP	5	38.0	scaffold69.0	204,205
Pyd05_024	SNP	5	42.2	scaffold1538.0	5,866
Pyd05_025	SNP	5	44.2	scaffold229.0	569,145
Pyd05_029	SNP	5	46.7	scaffold671.0	165,974
Pyd05_031	SNP	5	47.2	scaffold299.0	299,694
Pyd05_034	SNP	5	50.8	scaffold139.0	13,350
Pyb05_119	SNP	5	53.9	scaffold6.0	486,786
Pyb05_121	SNP	5	55.0	scaffold6.0	502,336
Pyb05_124	SNP	5	55.7	scaffold6.0	1,286,204
Pyb05_125	SNP	5	55.9	scaffold6.0	1,375,241
Pyb05_126	SNP	5	55.9	scaffold6.0	638,384
Pyb05_128	SNP	5	55.9	scaffold6.0	1,073,189
Pyb05_129	SNP	5	55.9	scaffold6.0	865,153
Pyb05_130	SNP	5	55.9	scaffold6.0	1,211,288
Pyb05_131	SNP	5	56.0	scaffold6.0	1,448,044

Pyb05_132	SNP	5	56.0	scaffold6.0	497,403
Pyb05_135	SNP	5	56.1	scaffold1173.0	97,037
Pyb05_137	SNP	5	56.2	scaffold1080.0	72,058
Pyb05_138	SNP	5	56.2	scaffold1080.0	72,114
Pyb05_139	SNP	5	56.3	scaffold6.0	1,280,767
Pyb05_140	SNP	5	56.4	scaffold6.0	582,414
Pyb05_141	SNP	5	56.5	scaffold69.0	1,052,284
Pyb05_142	SNP	5	59.2	scaffold69.0	764,696
Pyb05_143	SNP	5	60.2	scaffold69.0	482,483
Pyb05_144	SNP	5	60.3	scaffold69.0	576,144
Pyb05_146	SNP	5	60.4	scaffold69.0	382,415
Pyb05_149	SNP	5	61.9	scaffold69.0	345,129
Pyb05_154	SNP	5	65.7	scaffold45.0	794,829
Pyb05_157	SNP	5	66.7	scaffold45.0	1,006,260
Pyb05_158	SNP	5	67.1	scaffold925.0	59,025
Pyb05_159	SNP	5	67.6	scaffold561.0	101,399
Pyb05_160	SNP	5	67.7	scaffold45.0	1,091,289
Pyb05_164	SNP	5	67.8	scaffold925.0	99,558
Pyb05_169	SNP	5	68.8	scaffold529.0	319,783
Pyb05_171	SNP	5	68.8	scaffold340.0	195,343
Pyb05_173	SNP	5	68.8	scaffold410.0	240,682
Pyb05_175	SNP	5	68.8	scaffold410.0	20,313
Pyb05_177	SNP	5	68.8	scaffold410.0	113,372
Pyb05_178	SNP	5	68.8	scaffold410.0	391,292
Pyb05_180	SNP	5	68.8	scaffold678.0	10,993
Pyb05_182	SNP	5	68.8	scaffold600.0	48,153
Pyb05_183	SNP	5	69.1	scaffold691.0	93,425
Pyb05_184	SNP	5	69.1	scaffold410.0	34,316
Pyb05_185	SNP	5	71.6	scaffold405.0	57,709
Pyb05_191	SNP	5	73.0	scaffold405.0	225,496
Pyb05_192	SNP	5	73.1	scaffold405.0	154,472
Pyb05_193	SNP	5	73.1	scaffold405.0	130,092
Pyd05_039	SNP	5	73.1	scaffold139.0	223,182
Pyb05_198	SNP	5	75.5	scaffold53.0	86,834
Pyd05_046	SNP	5	78.2	scaffold28.0	468,711
Pyb05_202	SNP	5	78.4	scaffold28.0	413,056
Pyb05_203	SNP	5	78.4	scaffold28.0	361,514
Pyb05_204	SNP	5	78.5	scaffold388.0	14,944
Pyb05_205	SNP	5	78.5	scaffold28.0	361,525
Pyb05_207	SNP	5	78.6	scaffold859.0	10,498
Pyb05_214	SNP	5	83.0	scaffold780.0	21,173
Pyd05_052	SNP	5	83.5	scaffold1119.0	99,682
Pyd05_053	SNP	5	83.6	scaffold405.0	158,928
Pyd05_054	SNP	5	83.7	scaffold1596.0	16,564

Pyd05_055	SNP	5	83.9	scaffold599.0	220,570
Pybd05_010	SNP	5	87.8	scaffold523.0	206,179
Pyb05_217	SNP	5	91.4	scaffold666.0	17,787
Pybd05_014	SNP	5	102.4	scaffold405.0	158,985
Pyb05_224	SNP	5	105.2	scaffold45.0	1,063,271
Pyb05_226	SNP	5	106.0	scaffold45.0	818,784
Pyb05_232	SNP	5	109.4	scaffold241.0	426,553
Pyb05_233	SNP	5	109.5	scaffold241.0	218,904
Pyb05_234	SNP	5	109.8	scaffold372.0	133,610
Pybd05_017	SNP	5	111.9	scaffold69.0	576,182
Pyb05_237	SNP	5	112.3	scaffold69.0	719,630
Pyb05_238	SNP	5	112.4	scaffold69.0	702,450
Pyb05_239	SNP	5	112.5	scaffold69.0	612,329
Pybd05_018	SNP	5	114.7	scaffold69.0	178,695
Pyb05_241	SNP	5	117.9	scaffold6.0	399,158
Pyb05_242	SNP	5	118.0	scaffold6.0	351,346
Pyb05_244	SNP	5	118.5	scaffold6.0	410,896
Pyb05_246	SNP	5	118.6	scaffold6.0	430,366
Pyb05_247	SNP	5	118.7	scaffold6.0	461,765
Pyb05_250	SNP	5	118.7	scaffold6.0	459,459
Pyb05_252	SNP	5	118.7	scaffold6.0	295,304
Pyb05_253	SNP	5	118.8	scaffold6.0	413,209
Pyb05_254	SNP	5	118.8	scaffold6.0	202,074
TsuENH086	SSR	5	118.9	scaffold6.0	1,043,997-1,044,355
Pyb05_255	SNP	5	118.9	scaffold6.0	206,117
Pybd05_020	SNP	5	120.0	scaffold1173.0	54,794
Pyd05_065	SNP	5	121.6	scaffold69.0	729,130
Pyd05_066	SNP	5	121.6	scaffold69.0	680,905
Pyb05_258	SNP	5	122.1	scaffold40.0	705,750
Pyb05_259	SNP	5	122.3	scaffold40.0	655,904
Pyb05_260	SNP	5	123.0	scaffold40.0	510,712
Pyb05_262	SNP	5	123.0	scaffold40.0	501,298
Pyb05_263	SNP	5	123.0	scaffold40.0	571,294
Pyb05_264	SNP	5	123.5	scaffold40.0	410,654
Pyb05_266	SNP	5	126.0	scaffold69.0	303,451
Pyb05_268	SNP	5	127.0	scaffold554.0	54,388
Pyb05_269	SNP	5	127.1	scaffold554.0	73,448
Pybd05_021	SNP	5	129.8	scaffold40.0	162,780
Pyd05_073	SNP	5	130.8	scaffold40.0	1,216,289
Pyd05_074	SNP	5	133.0	scaffold92.0	279,792
Pyd05_076	SNP	5	137.3	scaffold92.0	808,601
Pyd05_077	SNP	5	137.3	scaffold110.0	929,331
Pyd05_078	SNP	5	137.4	scaffold110.0	773,805
Pyb05_271	SNP	5	139.7	scaffold430.0	373,131

EMPc106	SSR	5	139.9	scaffold92.0	68,217-68,357
Pyb05_273	SNP	5	141.6	scaffold6.0	464,760
Pyb05_274	SNP	5	141.6	scaffold430.0	109,494
Pyb05_275	SNP	5	141.9	scaffold430.0	387,930
Pyb05_276	SNP	5	142.0	scaffold6.0	120,918
Pyb05_284	SNP	5	143.7	scaffold40.0	698,329
Pyb05_285	SNP	5	143.9	scaffold40.0	693,537
Pyb05_287	SNP	5	145.2	scaffold40.0	242,724
Pybd05_023	SNP	5	145.6	scaffold110.0	673,140
Pyb05_293	SNP	5	147.0	scaffold110.0	110,651
Pyb05_295	SNP	5	147.1	scaffold110.0	113,908
Pyb05_297	SNP	5	147.4	scaffold110.0	386,150
Pyb05_298	SNP	5	147.4	scaffold110.0	828,098
Pyb05_301	SNP	5	147.6	scaffold92.0	778,297
Pyb05_303	SNP	5	147.6	scaffold92.0	632,878
Pyb05_304	SNP	5	147.6	scaffold92.0	669,709
Pyb05_305	SNP	5	147.6	scaffold92.0	597,256
Pyb05_306	SNP	5	147.6	scaffold92.0	891,775
Pyb05_307	SNP	5	147.7	scaffold92.0	351,919
Pyb05_308	SNP	5	147.7	scaffold56.0	65,089
Pyb05_312	SNP	5	147.9	scaffold110.0	413,387
Pyb05_314	SNP	5	147.9	scaffold92.0	450,647
Pyb05_316	SNP	5	147.9	scaffold92.0	800,875
Pyb05_317	SNP	5	147.9	scaffold92.0	638,329
Pyb05_318	SNP	5	148.0	scaffold110.0	636,041
Pyb05_321	SNP	5	148.0	scaffold110.0	182,658
Pyb05_322	SNP	5	148.0	scaffold92.0	614,236
Pyb05_323	SNP	5	148.0	scaffold110.0	691,743
Pyb05_324	SNP	5	148.0	scaffold110.0	800,511
Pyb05_326	SNP	5	148.0	scaffold110.0	636,082
Pyb05_327	SNP	5	148.0	scaffold110.0	710,623
Pyb05_328	SNP	5	148.0	scaffold110.0	699,949
Pyb05_329	SNP	5	148.1	scaffold110.0	742,697
Pyb05_331	SNP	5	148.1	scaffold110.0	563,373
Pyb05_332	SNP	5	148.1	scaffold110.0	774,306
CH04g09	SSR	5	148.2	scaffold448.0	380,563-380,679
Pyb05_336	SNP	5	148.4	scaffold40.0	350,093
Pyb05_338	SNP	5	148.4	scaffold92.0	259,689
Pyb05_340	SNP	5	148.6	scaffold40.0	416,193
Pyb05_341	SNP	5	148.6	scaffold40.0	385,511
Pyb05_343	SNP	5	148.6	scaffold40.0	453,095
Pyb05_345	SNP	5	148.7	scaffold40.0	401,702
Pybd05_024	SNP	5	148.8	scaffold110.0	95,123
Pyb05_350	SNP	5	149.5	scaffold110.0	87,117

Pyb05_351	SNP	5	149.9	scaffold110.0	117,877
Pyb05_352	SNP	5	150.0	scaffold56.0	21,844
Pyb05_354	SNP	5	151.2	scaffold56.0	131,850
Pyb05_356	SNP	5	151.4	scaffold56.0	147,417
Pyb05_357	SNP	5	151.6	scaffold216.0	3,597
Pyb05_358	SNP	5	151.6	scaffold97.0	291,006
Pyb05_359	SNP	5	151.7	scaffold97.0	166,403
Pyb05_360	SNP	5	151.7	scaffold97.0	58,460
Pyb05_361	SNP	5	151.9	scaffold97.0	429,731
Pyb05_362	SNP	5	152.1	scaffold593.0	10,431
Pyb05_365	SNP	5	152.9	scaffold97.0	903,911
Pybd05_026	SNP	5	156.0	scaffold97.0	152,346
Pybd05_027	SNP	5	156.3	scaffold1.0	1,209,639
Pyb05_368	SNP	5	161.4	scaffold1313.0	13,263
Pyb05_370	SNP	5	161.5	scaffold1650.0	1,004
Pyb05_371	SNP	5	161.6	scaffold97.0	397,070
Pyb05_372	SNP	5	161.8	scaffold1698.0	4,890
Pyb05_373	SNP	5	161.8	scaffold216.0	3,631
Pyb05_374	SNP	5	161.9	scaffold97.0	170,553
Pyb05_375	SNP	5	162.1	scaffold1.0	4,114,200
Pyb05_376	SNP	5	162.4	scaffold97.0	203,429
Pyb05_379	SNP	5	162.6	scaffold97.0	470,663
Pyb05_380	SNP	5	162.6	scaffold97.0	741,019
Pyb05_386	SNP	5	162.8	scaffold97.0	691,486
Pyb05_387	SNP	5	162.8	scaffold97.0	522,466
Pyb05_389	SNP	5	162.8	scaffold97.0	502,339
Pyb05_390	SNP	5	163.1	scaffold1.0	3,930,775
Pyb05_394	SNP	5	163.6	scaffold1.0	4,080,130
Pyb05_395	SNP	5	163.6	scaffold1.0	3,999,158
Pyb05_396	SNP	5	163.6	scaffold1.0	3,736,120
Pyb05_398	SNP	5	163.7	scaffold1.0	3,907,827
Pyb05_400	SNP	5	163.7	scaffold1.0	3,990,985
Pyb05_401	SNP	5	163.7	scaffold1.0	3,882,333
Pyb05_404	SNP	5	163.9	scaffold1.0	4,004,763
Pyb05_405	SNP	5	164.0	scaffold1.0	4,100,458
Pyb05_409	SNP	5	164.1	scaffold1.0	4,109,939
Pyb05_410	SNP	5	164.4	scaffold1.0	3,669,638
Pyb05_411	SNP	5	164.4	scaffold1.0	3,765,974
Hi09b04	SSR	5	165.5	scaffold1.0	2,776,229-2,775,873
Pyb05_415	SNP	5	166.5	scaffold1.0	3,541,400
Pyb05_416	SNP	5	166.6	scaffold1.0	3,458,406
Pyb05_419	SNP	5	167.0	scaffold1.0	2,849,681
Pyb05_420	SNP	5	167.1	scaffold1.0	3,437,243
Pyb05_421	SNP	5	167.1	scaffold1.0	3,437,221

Pyb05_423	SNP	5	167.1	scaffold1.0	3,402,067
Pyb05_425	SNP	5	167.1	scaffold1.0	3,466,097
Pyb05_427	SNP	5	167.2	scaffold1.0	3,391,916
Pyb05_428	SNP	5	167.2	scaffold1.0	3,418,214
Pyb05_430	SNP	5	167.6	scaffold1.0	2,571,854
Pyb05_431	SNP	5	167.8	scaffold1.0	2,915,515
Pyb05_433	SNP	5	167.8	scaffold1.0	3,300,242
Pyb05_437	SNP	5	168.0	scaffold1.0	2,390,065
Pyb05_439	SNP	5	168.1	scaffold1.0	3,330,261
Pyb05_441	SNP	5	168.1	scaffold1.0	3,042,100
Pyb05_442	SNP	5	168.4	scaffold1.0	2,758,877
Pyb05_448	SNP	5	169.3	scaffold1.0	2,504,217
Pyb05_449	SNP	5	169.5	scaffold1.0	2,122,134
Pyb05_455	SNP	5	170.6	scaffold1.0	539,478
Pyb05_456	SNP	5	170.6	scaffold1.0	1,156,339
Pyb05_458	SNP	5	170.7	scaffold1.0	1,453,219
Pyb05_460	SNP	5	170.7	scaffold1.0	102,599
Pyb05_464	SNP	5	170.9	scaffold1.0	1,642,728
Pyb05_465	SNP	5	170.9	scaffold1.0	40,489
Pyb05_466	SNP	5	170.9	scaffold1.0	532,737
Pyb05_467	SNP	5	170.9	scaffold1.0	252,473
Pyb05_468	SNP	5	170.9	scaffold1.0	522,732
Pyb05_469	SNP	5	171.0	scaffold1.0	890,253
Pyb05_470	SNP	5	171.0	scaffold1.0	1,160,091
Pyb05_474	SNP	5	171.5	scaffold1.0	312,520
Pyb05_475	SNP	5	171.5	scaffold1.0	172,474
Pyb05_476	SNP	5	171.5	scaffold1.0	247,689
Pyb05_477	SNP	5	171.6	scaffold1.0	539,211
Pyb05_478	SNP	5	171.8	scaffold1.0	1,647,741
Pyb05_480	SNP	5	172.0	scaffold1.0	239,906
Pyb05_481	SNP	5	172.0	scaffold1.0	325,212
Pyb05_482	SNP	5	172.1	scaffold1.0	1,154,626
Pyb05_483	SNP	5	172.1	scaffold1.0	1,156,311
Pybd05_030	SNP	5	172.8	scaffold97.0	39,061
Pyd05_094	SNP	5	177.1	scaffold97.0	893,290
Pyd06_007	SNP	6	0.0	scaffold180.0	322,168
Pyd06_008	SNP	6	0.0	scaffold180.0	295,352
CH03d12	SSR	6	0.8	scaffold263.0	498,540-498,718
Pyb06_011	SNP	6	1.2	scaffold760.0	99,646
Pyb06_012	SNP	6	1.2	scaffold263.0	279,653
Pyb06_013	SNP	6	1.2	scaffold444.0	233,033
Pyb06_015	SNP	6	1.5	scaffold444.0	132,184
Pyb06_028	SNP	6	9.7	scaffold180.0	294,546
Pyb06_030	SNP	6	9.7	scaffold180.0	147,141

Pyb06_031	SNP	6	9.8	scaffold180.0	281,803
Pyb06_033	SNP	6	10.5	scaffold511.0	172,740
Pyb06_035	SNP	6	11.4	scaffold47.0	757,366
Pyb06_036	SNP	6	11.7	scaffold47.0	84,483
Pyb06_039	SNP	6	11.8	scaffold47.0	398,092
Pyb06_041	SNP	6	12.0	scaffold30.0	1,061,718
Pyb06_042	SNP	6	12.1	scaffold82.0	210,814
U78949x	SSR	6	12.1	scaffold30.0	261,825-262,021
Pyb06_045	SNP	6	12.2	scaffold47.0	316,385
Pyb06_047	SNP	6	12.3	scaffold47.0	94,860
Pyb06_048	SNP	6	12.3	scaffold47.0	513,472
Pyb06_049	SNP	6	12.3	scaffold30.0	11,449
Pyb06_052	SNP	6	12.4	scaffold47.0	83,110
Pyb06_054	SNP	6	12.4	scaffold30.0	571,302
Pyb06_056	SNP	6	12.4	scaffold30.0	846,392
Pyb06_059	SNP	6	12.4	scaffold30.0	72,859
Pyb06_060	SNP	6	12.5	scaffold511.0	2,868
Pyb06_061	SNP	6	12.5	scaffold30.0	250,698
Pyb06_062	SNP	6	12.5	scaffold30.0	143,766
Pyb06_065	SNP	6	12.5	scaffold30.0	240,803
Pyb06_066	SNP	6	12.5	scaffold30.0	229,107
Pyb06_067	SNP	6	12.6	scaffold30.0	143,813
Pyb06_068	SNP	6	12.6	scaffold511.0	17,185
Pyb06_070	SNP	6	12.6	scaffold30.0	216,073
Pyb06_073	SNP	6	12.8	scaffold30.0	730,951
Pyb06_074	SNP	6	12.8	scaffold30.0	942,498
Pyb06_075	SNP	6	12.9	scaffold47.0	400,927
Pyb06_076	SNP	6	12.9	scaffold30.0	1,273,156
Pyb06_077	SNP	6	12.9	scaffold30.0	745,264
Pyb06_078	SNP	6	12.9	scaffold47.0	121,447
Pyb06_080	SNP	6	12.9	scaffold30.0	1,003,321
Pyb06_081	SNP	6	12.9	scaffold30.0	1,273,128
Pyb06_083	SNP	6	12.9	scaffold47.0	77,577
Pyb06_085	SNP	6	13.2	scaffold30.0	1,028,209
Pyb06_086	SNP	6	13.2	scaffold47.0	812,162
Pyb06_087	SNP	6	13.2	scaffold30.0	411,104
Pyb06_090	SNP	6	13.6	scaffold180.0	114,893
Pyb06_093	SNP	6	14.0	scaffold180.0	65,325
Pyb06_094	SNP	6	14.0	scaffold511.0	195,730
Pyb06_095	SNP	6	14.2	scaffold511.0	184,816
Pyb06_096	SNP	6	14.7	scaffold30.0	654,726
Pyb06_097	SNP	6	14.8	scaffold30.0	705,653
Pyb06_100	SNP	6	16.6	scaffold30.0	877,306
Pyb06_103	SNP	6	17.7	scaffold30.0	1,271,006

Pybd06_005	SNP	6	19.5	scaffold30.0	601,634
Pybd06_006	SNP	6	20.1	scaffold30.0	135,699
Pybd06_007	SNP	6	21.7	scaffold30.0	1,288,081
Pyb06_106	SNP	6	23.8	scaffold511.0	324,480
Pybd06_008	SNP	6	26.4	scaffold47.0	553,919
Pybd06_009	SNP	6	28.8	scaffold47.0	903,721
Pyd06_017	SNP	6	29.6	scaffold67.0	647,937
Pyd06_020	SNP	6	31.5	scaffold67.0	1,093,237
Pyd06_024	SNP	6	33.6	scaffold642.0	46,169
Pyd06_026	SNP	6	33.8	scaffold642.0	50,044
Pyb06_109	SNP	6	35.9	scaffold180.0	383,516
Pyb06_110	SNP	6	36.0	scaffold180.0	500,172
Pyb06_112	SNP	6	36.2	scaffold180.0	484,193
Pyb06_113	SNP	6	36.3	scaffold180.0	436,047
Pybd06_010	SNP	6	36.6	scaffold373.0	24,475
Pyb06_117	SNP	6	37.3	scaffold180.0	554,111
Pyb06_120	SNP	6	37.9	scaffold180.0	580,575
CTG1063987	SSR	6	41.5	scaffold373.0	194,270-194,396
Pyb06_134	SNP	6	42.1	scaffold373.0	217,199
Pyd06_032	SNP	6	42.2	scaffold67.0	742,259
Pyb06_136	SNP	6	42.4	scaffold373.0	238,621
Pyb06_138	SNP	6	42.5	scaffold373.0	359,243
Pyb06_142	SNP	6	43.2	scaffold373.0	314,075
Pyb06_149	SNP	6	43.6	scaffold373.0	44,164
Pyb06_150	SNP	6	43.7	scaffold506.0	256,904
Pyb06_151	SNP	6	43.7	scaffold506.0	162,608
Pyb06_152	SNP	6	43.7	scaffold506.0	226,734
Pyb06_154	SNP	6	44.1	scaffold373.0	16,480
Pyb06_155	SNP	6	44.1	scaffold172.0	100,783
Pyb06_156	SNP	6	44.1	scaffold74.0	370,955
Pyb06_159	SNP	6	44.2	scaffold506.0	286,551
Pyb06_162	SNP	6	45.7	scaffold81.0	558,459
Pyb06_163	SNP	6	45.9	scaffold67.0	1,025,002
Pyb06_164	SNP	6	46.0	scaffold67.0	1,094,220
Pyb06_165	SNP	6	46.0	scaffold81.0	417,197
Pyb06_166	SNP	6	46.1	scaffold81.0	295,028
Pyd06_038	SNP	6	46.1	scaffold81.0	469,190
Pyb06_168	SNP	6	46.1	scaffold81.0	485,795
Pyb06_169	SNP	6	46.1	scaffold81.0	238,230
Pyb06_170	SNP	6	46.1	scaffold75.0	880,248
Pyb06_171	SNP	6	46.2	scaffold81.0	439,264
Pyb06_173	SNP	6	46.3	scaffold81.0	454,653
Pyb06_174	SNP	6	46.3	scaffold67.0	758,255
Pyb06_175	SNP	6	46.3	scaffold81.0	61,264

Pyd06_039	SNP	6	46.3	scaffold506.0	117,776
Pyb06_177	SNP	6	46.3	scaffold81.0	503,800
Pyb06_181	SNP	6	46.6	scaffold67.0	355,354
Pyb06_182	SNP	6	46.6	scaffold67.0	369,909
Pyd06_040	SNP	6	46.8	scaffold172.0	39,404
Pyb06_184	SNP	6	47.3	scaffold81.0	255,682
Pyb06_186	SNP	6	48.4	scaffold67.0	382,781
Pyb06_188	SNP	6	48.8	scaffold1160.0	47,211
Pyb06_189	SNP	6	48.9	scaffold1160.0	93,046
Pyb06_191	SNP	6	49.3	scaffold21.0	273,149
Pyb06_192	SNP	6	49.3	scaffold21.0	184,602
Pyb06_193	SNP	6	49.4	scaffold21.0	304,799
Pyb06_194	SNP	6	49.4	scaffold21.0	261,648
Pyb06_196	SNP	6	49.5	scaffold21.0	179,709
Pyb06_199	SNP	6	50.3	scaffold389.0	203,541
Pyb06_202	SNP	6	50.5	scaffold21.0	142,755
Pyb06_203	SNP	6	50.7	scaffold389.0	201,539
Pyb06_206	SNP	6	51.1	scaffold389.0	190,525
Pyb06_207	SNP	6	51.1	scaffold389.0	84,800
Pyb06_208	SNP	6	51.2	scaffold21.0	153,690
Pyb06_209	SNP	6	51.4	scaffold389.0	185,893
Pyb06_210	SNP	6	51.7	scaffold729.0	87,001
Pyb06_211	SNP	6	51.8	scaffold1318.0	68,448
Pyb06_212	SNP	6	52.2	scaffold389.0	310,255
CH01b11	SSR	6	52.8	scaffold67.0	703,750-703,931
Pybd06_014	SNP	6	52.8	scaffold67.0	590,039
Pybd06_015	SNP	6	54.3	scaffold67.0	589,975
Pybd06_016	SNP	6	56.8	scaffold67.0	385,227
Pyb06_221	SNP	6	65.7	scaffold176.0	453,131
Pyb06_222	SNP	6	65.7	scaffold540.0	44,697
Pyb06_227	SNP	6	67.8	scaffold729.0	86,974
Pyb06_228	SNP	6	67.8	scaffold109.0	647,544
Pyb06_229	SNP	6	67.8	scaffold109.0	484,290
Pyb06_237	SNP	6	70.1	scaffold932.0	26,268
Pyb06_238	SNP	6	70.1	scaffold932.0	27,094
Pyb06_239	SNP	6	70.3	scaffold932.0	113,719
Pybd06_018	SNP	6	70.9	scaffold176.0	502,030
Pyb06_241	SNP	6	71.3	scaffold1356.0	45,522
Pyb06_244	SNP	6	71.9	scaffold540.0	32,377
Pyb06_248	SNP	6	72.0	scaffold540.0	1,560
Pybd06_019	SNP	6	72.3	scaffold540.0	11,473
Pyb06_252	SNP	6	72.6	scaffold176.0	486,828
Pybd06_020	SNP	6	73.1	scaffold176.0	623,403
Pyb06_266	SNP	6	77.7	scaffold104.0	160,733

Pyb06_267	SNP	6	77.8	scaffold104.0	85,081
Pyb06_281	SNP	6	81.1	scaffold582.0	21,375
Pyb06_283	SNP	6	81.5	scaffold7.0	444,253
Pyb06_284	SNP	6	81.6	scaffold946.0	133,185
Pyb06_286	SNP	6	81.7	scaffold201.0	504,936
Pyb06_290	SNP	6	81.9	scaffold894.0	142,236
Pyb06_291	SNP	6	82.0	scaffold215.0	188,506
Pyb06_292	SNP	6	82.0	scaffold7.0	367,453
Pyb06_294	SNP	6	82.1	scaffold672.0	92,977
Pyb06_295	SNP	6	82.1	scaffold1523.0	10,370
Pyb06_300	SNP	6	82.7	scaffold894.0	87,366
Pyb06_301	SNP	6	82.7	scaffold215.0	36,889
Pyb06_302	SNP	6	82.8	scaffold737.0	86,847
Pyb06_306	SNP	6	83.0	scaffold672.0	81,588
Pyd06_057	SNP	6	83.0	scaffold7.0	348,899
Pyb06_308	SNP	6	83.0	scaffold7.0	254,717
Pyb06_311	SNP	6	83.1	scaffold880.0	138,819
Pyb06_313	SNP	6	83.2	scaffold525.0	203,418
Pyb06_314	SNP	6	83.2	scaffold182.0	10,389
Pyb06_316	SNP	6	83.4	scaffold215.0	106,442
Pyb06_317	SNP	6	83.5	scaffold894.0	84,820
Pyb06_318	SNP	6	83.5	scaffold215.0	18,867
Pyb06_320	SNP	6	83.7	scaffold582.0	11,538
Pyb06_321	SNP	6	83.8	scaffold215.0	192,502
Pyb06_322	SNP	6	83.9	scaffold215.0	115,003
Pyb06_323	SNP	6	83.9	scaffold894.0	129,413
Pyd06_059	SNP	6	86.0	scaffold215.0	617,202
CTG1070694	SSR	6	96.1	scaffold136.0	379,291-379,516
Pyd06_062	SNP	6	96.2	scaffold136.0	497,617
Pyd06_063	SNP	6	96.5	scaffold136.0	364,308
Pyd06_064	SNP	6	96.8	scaffold136.0	292,858
Pybd06_026	SNP	6	101.0	scaffold240.0	291,698
Pyd06_072	SNP	6	102.8	scaffold203.0	554,888
Pyb06_330	SNP	6	106.4	scaffold223.0	351,021
Pyb06_335	SNP	6	107.0	scaffold223.0	44,147
Pyb06_336	SNP	6	107.2	scaffold240.0	488,645
Pyb06_337	SNP	6	107.5	scaffold136.0	537,478
Pyb06_338	SNP	6	107.5	scaffold136.0	289,908
Pyb06_339	SNP	6	107.5	scaffold136.0	750,004
Pyb06_341	SNP	6	107.6	scaffold223.0	391,199
Pyb06_344	SNP	6	107.8	scaffold223.0	44,210
Pyb06_345	SNP	6	107.9	scaffold136.0	374,520
Pyb06_348	SNP	6	108.3	scaffold136.0	309,006
Pyb06_349	SNP	6	108.3	scaffold136.0	292,903

Pyb06_350	SNP	6	108.3	scaffold136.0	358,167
Pyb06_353	SNP	6	108.5	scaffold223.0	404,234
Pyb06_355	SNP	6	108.6	scaffold223.0	577,801
Pyb06_356	SNP	6	108.8	scaffold136.0	29,135
Pyb06_358	SNP	6	109.2	scaffold136.0	290,003
Pybd06_028	SNP	6	109.6	scaffold240.0	134,758
Pyb06_360	SNP	6	110.9	scaffold203.0	338,368
Pyb06_365	SNP	6	111.6	scaffold240.0	279,115
Pyb06_372	SNP	6	112.2	scaffold240.0	308,593
Pyb06_374	SNP	6	112.4	scaffold240.0	192,137
Pyb06_375	SNP	6	112.4	scaffold240.0	23,717
Pyb06_379	SNP	6	113.1	scaffold240.0	35,620
Pyb06_380	SNP	6	113.1	scaffold240.0	119,798
Pyb06_381	SNP	6	113.4	scaffold203.0	113,762
Pybd06_029	SNP	6	113.9	scaffold215.0	27,963
Pyb06_386	SNP	6	115.2	scaffold362.0	179,300
Pyb06_389	SNP	6	115.6	scaffold7.0	241,908
Pyb06_397	SNP	6	115.8	scaffold7.0	273,179
Pybd06_031	SNP	6	115.9	scaffold201.0	405,962
Pyb06_399	SNP	6	116.4	scaffold737.0	155,856
Pyb06_400	SNP	6	116.5	scaffold215.0	177,750
Pybd06_032	SNP	6	117.4	scaffold215.0	394,576
Pyb06_403	SNP	6	117.8	scaffold574.0	129,807
Pyb06_405	SNP	6	118.0	scaffold36.0	1,067,422
Pyb06_408	SNP	6	120.4	scaffold7.0	444,203
Pyb07_002	SNP	7	0.0	scaffold37.0	686,864
Pyb07_003	SNP	7	1.7	scaffold37.0	655,651
Pyb07_004	SNP	7	2.4	scaffold37.0	538,682
Pyb07_005	SNP	7	3.3	scaffold37.0	384,928
Pyb07_010	SNP	7	5.4	scaffold37.0	25,984
Pyb07_012	SNP	7	5.8	scaffold95.0	544,881
Pyb07_013	SNP	7	6.3	scaffold37.0	490,260
Pyb07_014	SNP	7	6.6	scaffold37.0	454,292
Pyb07_015	SNP	7	6.7	scaffold37.0	274,800
Pyb07_016	SNP	7	6.8	scaffold37.0	803,232
Pyb07_017	SNP	7	7.2	scaffold161.0	568,034
Pyb07_019	SNP	7	7.7	scaffold37.0	769,798
Pyb07_020	SNP	7	8.0	scaffold37.0	204,331
Pyb07_022	SNP	7	8.4	scaffold37.0	790,867
Pyb07_023	SNP	7	8.7	scaffold37.0	741,059
Pyb07_025	SNP	7	9.2	scaffold37.0	648,456
Pyb07_026	SNP	7	9.4	scaffold95.0	808,433
Pyb07_027	SNP	7	9.7	scaffold161.0	45,995
Pyb07_028	SNP	7	9.9	scaffold161.0	155,316

Pyb07_029	SNP	7	10.0	scaffold161.0	233,323
Pyb07_031	SNP	7	10.1	scaffold161.0	164,769
Pyb07_032	SNP	7	10.2	scaffold161.0	61,888
Pyb07_033	SNP	7	10.3	scaffold95.0	899,087
Pyb07_034	SNP	7	10.5	scaffold161.0	392,059
Pyb07_035	SNP	7	10.8	scaffold161.0	179,410
Pyb07_038	SNP	7	11.5	scaffold95.0	114,605
Pyb07_040	SNP	7	11.6	scaffold95.0	534,248
Pyb07_041	SNP	7	11.7	scaffold95.0	524,875
Pyb07_043	SNP	7	12.1	scaffold95.0	62,814
Pyb07_044	SNP	7	12.5	scaffold95.0	510,146
Pyb07_045	SNP	7	13.4	scaffold454.0	298,394
Pyb07_052	SNP	7	15.6	scaffold454.0	303,719
Pyb07_055	SNP	7	17.0	scaffold90.0	902,764
Pyb07_057	SNP	7	17.3	scaffold454.0	106,302
Pyb07_058	SNP	7	17.9	scaffold90.0	55,731
Pyb07_060	SNP	7	18.2	scaffold90.0	568,606
Pyb07_061	SNP	7	18.5	scaffold90.0	512,897
Pyb07_064	SNP	7	19.3	scaffold90.0	394,841
TsuENH006	SSR	7	19.9	scaffold90.0	687,239-687,378
Pyb07_065	SNP	7	20.2	scaffold447.0	278,207
Pyb07_066	SNP	7	20.5	scaffold90.0	383,396
Pyb07_068	SNP	7	21.9	scaffold276.0	315,120
Pyb07_073	SNP	7	24.3	scaffold1012.0	11,534
Pyb07_075	SNP	7	25.0	scaffold237.0	293,366
Pyb07_077	SNP	7	25.6	scaffold237.0	305,253
Pyb07_078	SNP	7	25.9	scaffold418.0	71,810
Pyb07_079	SNP	7	25.9	scaffold728.0	35,901
Pyb07_081	SNP	7	26.0	scaffold728.0	40,771
Pyb07_083	SNP	7	26.6	scaffold1012.0	4,220
Pyb07_085	SNP	7	27.1	scaffold237.0	93,546
Pyb07_086	SNP	7	27.3	scaffold1150.0	14,026
Pyb07_087	SNP	7	27.7	scaffold237.0	299,029
Pyb07_088	SNP	7	28.0	scaffold237.0	78,582
Pyb07_089	SNP	7	28.3	scaffold1150.0	40,460
Pyb07_091	SNP	7	29.1	scaffold1150.0	50,304
Pyb07_093	SNP	7	30.8	scaffold278.0	49,975
Pyb07_094	SNP	7	31.5	scaffold278.0	393,182
Pyb07_095	SNP	7	32.0	scaffold278.0	502,462
Pyb07_100	SNP	7	34.3	scaffold668.0	38,606
Pyb07_102	SNP	7	35.3	scaffold278.0	38,587
Pyb07_104	SNP	7	38.3	scaffold563.0	226,344
Pyb07_105	SNP	7	38.5	scaffold563.0	130,015
Pyb07_106	SNP	7	38.6	scaffold709.0	65,654

Pyb07_108	SNP	7	39.0	scaffold1193.0	24,930
Pyb07_109	SNP	7	39.2	scaffold496.0	318,964
Pyb07_110	SNP	7	39.8	scaffold146.0	597,089
Pyb07_112	SNP	7	39.9	scaffold146.0	461,771
Pyb07_114	SNP	7	40.5	scaffold496.0	232,823
Pyb07_115	SNP	7	41.0	scaffold146.0	462,543
Pyb07_116	SNP	7	41.7	scaffold326.0	349,673
Pyb07_117	SNP	7	42.2	scaffold1092.0	76,490
Pyb07_119	SNP	7	42.6	scaffold949.0	19,875
Pyb07_120	SNP	7	42.9	scaffold557.0	203,263
Pyb07_121	SNP	7	43.3	scaffold326.0	284,477
Pyb07_122	SNP	7	43.8	scaffold550.0	250,758
Pyb07_124	SNP	7	45.1	scaffold550.0	141,773
Pyb07_129	SNP	7	50.2	scaffold58.0	639,207
Pyb07_130	SNP	7	50.5	scaffold429.0	1,875
Pyb07_132	SNP	7	51.4	scaffold58.0	930,743
Pyb07_133	SNP	7	51.7	scaffold58.0	988,674
Pyb07_134	SNP	7	51.9	scaffold58.0	891,986
Pyb07_135	SNP	7	53.3	scaffold18.0	1,485,470
EMPc117	SSR	7	53.4	scaffold58.0	1,068,166-1,067,743
Pyb07_136	SNP	7	53.9	scaffold18.0	1,355,569
Pyb07_138	SNP	7	54.4	scaffold18.0	1,284,097
Pyb07_139	SNP	7	54.7	scaffold175.0	587,095
Pyb07_140	SNP	7	55.0	scaffold175.0	169,144
Pyb07_141	SNP	7	55.2	scaffold18.0	653,934
Pyb07_142	SNP	7	55.5	scaffold18.0	834,637
Pyb07_143	SNP	7	55.5	scaffold18.0	744,242
Pyb07_144	SNP	7	55.9	scaffold175.0	233,421
Pyb07_146	SNP	7	56.2	scaffold175.0	283,336
Pyb07_147	SNP	7	56.4	scaffold175.0	145,690
Pyb07_149	SNP	7	56.8	scaffold175.0	609,221
Pyb07_150	SNP	7	57.1	scaffold18.0	915,836
Pyb07_152	SNP	7	57.5	scaffold175.0	474,015
Pyb07_156	SNP	7	58.2	scaffold175.0	340,311
EMPc111	SSR	7	58.5	scaffold112.0	297,012-297,107
Pyb07_158	SNP	7	58.5	scaffold112.0	414,733
Pyb07_159	SNP	7	59.1	scaffold175.0	613,553
Pyb07_160	SNP	7	59.8	scaffold112.0	298,506
Pyb07_167	SNP	7	63.1	scaffold205.0	104,863
Pyb07_169	SNP	7	63.4	scaffold205.0	195,631
Pyb07_170	SNP	7	63.6	scaffold205.0	367,450
Pyb07_172	SNP	7	64.3	scaffold285.0	196,331
Pyb07_173	SNP	7	64.6	scaffold285.0	196,604
Pyb07_175	SNP	7	65.7	scaffold682.0	146,199

Pyb07_176	SNP	7	66.2	scaffold682.0	146,158
Pyb07_177	SNP	7	66.6	scaffold87.0	618,362
Pyb07_179	SNP	7	67.5	scaffold87.0	932,906
Pyb07_180	SNP	7	67.7	scaffold174.0	466,483
Pyb07_181	SNP	7	67.8	scaffold174.0	114,642
Pyb07_182	SNP	7	67.9	scaffold87.0	970,458
Pyb07_183	SNP	7	68.1	scaffold87.0	733,498
Pyb07_184	SNP	7	68.3	scaffold174.0	353,887
Pyb07_185	SNP	7	68.4	scaffold174.0	373,420
Pyb07_186	SNP	7	68.6	scaffold56.0	787,414
Pyb07_188	SNP	7	68.9	scaffold174.0	555,659
Pyb07_189	SNP	7	69.2	scaffold87.0	793,638
Pyb07_193	SNP	7	69.9	scaffold87.0	757,350
Pyb07_194	SNP	7	70.2	scaffold87.0	733,034
Pyb07_195	SNP	7	70.5	scaffold87.0	773,372
Pyb07_196	SNP	7	70.8	scaffold87.0	60,216
Pyb07_197	SNP	7	70.9	scaffold87.0	498,086
Pyb07_198	SNP	7	71.0	scaffold87.0	539,425
Pyb07_199	SNP	7	71.3	scaffold87.0	748,717
Pyb07_200	SNP	7	71.6	scaffold87.0	755,838
Pyb07_201	SNP	7	71.9	scaffold87.0	797,614
Pyb07_202	SNP	7	72.1	scaffold87.0	287,700
Pyb07_203	SNP	7	72.5	scaffold87.0	320,823
Pyb07_204	SNP	7	73.1	scaffold87.0	982,855
Pyb08_001	SNP	8	0.0	scaffold238.0	189,025
Pyb08_005	SNP	8	0.8	scaffold293.0	118,875
Pyb08_007	SNP	8	1.0	scaffold416.0	204,762
Pyb08_009	SNP	8	1.5	scaffold12.0	1,071,706
Pyb08_011	SNP	8	1.6	scaffold416.0	108,732
CH01h10	SSR	8	2.0	scaffold416.0	292,612-292,728
Pyb08_018	SNP	8	3.6	scaffold238.0	315,210
Pyb08_020	SNP	8	8.8	scaffold43.0	42,115
Pyb08_023	SNP	8	9.3	scaffold12.0	1,493,202
Pyb08_024	SNP	8	9.3	scaffold43.0	692,194
Pyb08_026	SNP	8	9.6	scaffold55.0	765,527
Pyb08_027	SNP	8	9.6	scaffold12.0	1,334,800
Pyb08_028	SNP	8	9.7	scaffold12.0	1,560,911
Pyb08_029	SNP	8	9.7	scaffold12.0	1,099,722
Pybd08_001	SNP	8	10.1	scaffold577.0	32,804
Pyb08_036	SNP	8	10.5	scaffold293.0	5,563
Pyb08_037	SNP	8	10.5	scaffold293.0	35,256
Pyb08_039	SNP	8	10.5	scaffold293.0	24,079
Pyb08_042	SNP	8	10.7	scaffold293.0	28,394
Pyb08_050	SNP	8	11.5	scaffold577.0	145,216

Pyb08_052	SNP	8	11.6	scaffold293.0	272,724
Pyb08_053	SNP	8	11.6	scaffold293.0	281,213
Pyb08_056	SNP	8	11.9	scaffold293.0	318,444
Pyb08_058	SNP	8	12.0	scaffold293.0	400,570
Pyb08_059	SNP	8	12.0	scaffold293.0	309,948
Pyb08_060	SNP	8	12.0	scaffold293.0	457,579
Pyb08_064	SNP	8	12.6	scaffold293.0	364,202
Pyb08_068	SNP	8	13.8	scaffold293.0	372,243
Pyb08_074	SNP	8	17.9	scaffold238.0	55,592
Pyb08_076	SNP	8	18.1	scaffold152.0	392,816
Pyb08_077	SNP	8	18.6	scaffold838.0	19,554
Pyb08_079	SNP	8	19.1	scaffold238.0	193,205
Pyb08_082	SNP	8	19.3	scaffold238.0	190,218
Pyb08_083	SNP	8	19.3	scaffold238.0	204,908
Pyb08_086	SNP	8	19.8	scaffold152.0	535,085
Pyb08_090	SNP	8	20.9	scaffold613.0	106,263
Pyb08_091	SNP	8	20.9	scaffold152.0	160,423
Pyb08_092	SNP	8	20.9	scaffold152.0	162,242
Pyb08_093	SNP	8	21.0	scaffold613.0	99,090
Pyb08_094	SNP	8	21.0	scaffold578.0	131,756
Pyb08_095	SNP	8	21.1	scaffold152.0	127,889
Pyb08_098	SNP	8	21.7	scaffold778.0	19,503
Pybd08_002	SNP	8	22.2	scaffold12.0	1,339,951
Pybd08_003	SNP	8	26.9	scaffold43.0	552,834
Pybd08_005	SNP	8	31.4	scaffold43.0	75,235
Pyb08_104	SNP	8	32.4	scaffold43.0	875,296
Pybd08_006	SNP	8	32.6	scaffold43.0	1,056,815
Pybd08_007	SNP	8	33.6	scaffold43.0	29,842
Pyb08_106	SNP	8	34.0	scaffold43.0	677,111
Pyb08_112	SNP	8	34.8	scaffold12.0	1,580,712
Pyb08_115	SNP	8	34.9	scaffold12.0	1,310,886
Pyb08_116	SNP	8	34.9	scaffold577.0	113,254
Pyb08_118	SNP	8	35.2	scaffold416.0	395,949
Pyb08_120	SNP	8	35.3	scaffold577.0	32,849
Pyb08_121	SNP	8	35.4	scaffold577.0	155,842
Pyb08_122	SNP	8	35.4	scaffold12.0	1,520,822
Pyb08_123	SNP	8	35.4	scaffold577.0	134,644
Pyb08_124	SNP	8	35.5	scaffold12.0	1,494,141
Pyb08_125	SNP	8	35.5	scaffold1049.0	84,119
Pyb08_126	SNP	8	35.5	scaffold12.0	1,177,550
Pyb08_127	SNP	8	35.5	scaffold416.0	128,845
Pyb08_128	SNP	8	35.6	scaffold43.0	927,433
Pyb08_130	SNP	8	35.6	scaffold577.0	127,037
Pyb08_131	SNP	8	35.6	scaffold43.0	1,156,498

Pyb08_132	SNP	8	35.6	scaffold43.0	756,967
Pyb08_134	SNP	8	35.9	scaffold43.0	1,166,996
Pyb08_135	SNP	8	35.9	scaffold43.0	842,667
Pyb08_136	SNP	8	36.0	scaffold43.0	855,769
Pyb08_138	SNP	8	36.3	scaffold43.0	887,061
Pyb08_139	SNP	8	36.3	scaffold43.0	889,314
Pybd08_009	SNP	8	38.5	scaffold43.0	272,006
Pyb08_145	SNP	8	44.7	scaffold238.0	54,117
Pyb08_147	SNP	8	46.0	scaffold238.0	351,116
Pyb08_148	SNP	8	46.0	scaffold152.0	443,123
Pyb08_149	SNP	8	46.7	scaffold838.0	125,098
Pyb08_150	SNP	8	47.2	scaffold613.0	81,425
Pyb08_152	SNP	8	47.4	scaffold778.0	2,845
Pyb08_154	SNP	8	47.6	scaffold778.0	19,455
Pyb08_155	SNP	8	47.7	scaffold613.0	135,847
Pyb08_157	SNP	8	49.1	scaffold1118.0	106,936
Pyb08_159	SNP	8	49.7	scaffold1118.0	89,994
Pyb08_164	SNP	8	51.6	scaffold68.0	213,403
Pyb08_165	SNP	8	51.8	scaffold99.0	361,121
Pyb08_171	SNP	8	52.0	scaffold99.0	508,390
Pyb08_177	SNP	8	52.3	scaffold99.0	420,710
Pyb08_178	SNP	8	52.4	scaffold99.0	361,185
Pyb08_179	SNP	8	52.5	scaffold99.0	14,043
Pyb08_181	SNP	8	53.1	scaffold99.0	420,670
Pybd08_011	SNP	8	53.8	scaffold99.0	87,367
Pyb08_185	SNP	8	54.3	scaffold99.0	199,405
Pyb08_187	SNP	8	57.0	scaffold304.0	376,634
Pyb08_203	SNP	8	60.4	scaffold318.0	214,677
Pyb08_204	SNP	8	60.4	scaffold318.0	216,692
Pybd08_012	SNP	8	60.5	scaffold227.0	231,894
Pyb08_216	SNP	8	64.3	scaffold502.0	35,446
CH05a02	SSR	8	64.5	scaffold502.0	124,284-124,014
Pyb08_218	SNP	8	64.6	scaffold502.0	19,103
Pyb08_219	SNP	8	64.6	scaffold27.0	1,275,963
Hi20b03	SSR	8	64.7	scaffold816.0	46,823-47,049
Pyb08_220	SNP	8	64.7	scaffold502.0	35,387
Pyb08_221	SNP	8	64.9	scaffold502.0	58,646
Pybd08_015	SNP	8	67.5	scaffold816.0	153,536
Pyd08_004	SNP	8	69.0	scaffold502.0	237,213
Pyb08_225	SNP	8	71.7	scaffold190.0	383,332
Pybd08_016	SNP	8	74.3	scaffold887.0	1,942
Pybd08_017	SNP	8	76.0	scaffold1304.0	64,603
Pyd08_011	SNP	8	78.6	scaffold190.0	386,928
Pyb08_233	SNP	8	81.9	scaffold887.0	72,534

Pyb08_245	SNP	8	85.7	scaffold33.0	722,729
Pyb08_248	SNP	8	85.9	scaffold33.0	600,236
Pyb08_252	SNP	8	86.5	scaffold398.0	100,585
Pyb08_254	SNP	8	86.6	scaffold49.2	94,935
Pyb08_257	SNP	8	86.9	scaffold33.0	603,233
Pyb08_258	SNP	8	87.0	scaffold398.0	344,963
Pybd08_021	SNP	8	87.0	scaffold398.0	415,954
Pyb08_260	SNP	8	87.0	scaffold269.0	462,999
Pyb08_264	SNP	8	87.2	scaffold269.0	530,503
Pyb08_265	SNP	8	87.2	scaffold49.2	286,913
Pyb08_266	SNP	8	87.3	scaffold49.2	298,996
Pyb08_267	SNP	8	87.4	scaffold49.2	43,129
Pyb08_268	SNP	8	87.4	scaffold49.2	303,544
Pyb08_270	SNP	8	87.6	scaffold398.0	66,078
Pyb08_272	SNP	8	87.7	scaffold398.0	134,924
Pyb08_273	SNP	8	87.7	scaffold398.0	406,168
Pyb08_275	SNP	8	87.9	scaffold398.0	74,861
Pyb08_276	SNP	8	87.9	scaffold398.0	134,279
Pyb08_277	SNP	8	88.0	scaffold398.0	387,156
Pyb08_279	SNP	8	88.3	scaffold33.0	600,248
Pyb08_285	SNP	8	89.2	scaffold190.0	386,897
Pybd08_023	SNP	8	95.3	scaffold131.0	391,386
Pybd08_024	SNP	8	100.2	scaffold131.0	708,811
Pyb08_311	SNP	8	100.6	scaffold464.0	39,473
NH036b	SSR	8	100.8	scaffold464.0	21,703-21,980
Pyb08_320	SNP	8	101.6	scaffold464.0	263,429
Pyb08_321	SNP	8	101.6	scaffold464.0	74,977
Pyb08_322	SNP	8	101.7	scaffold464.0	87,576
Pyb08_323	SNP	8	101.8	scaffold464.0	117,033
Pyb08_324	SNP	8	102.0	scaffold464.0	87,536
Pyb08_325	SNP	8	102.2	scaffold464.0	95,544
Pyb08_328	SNP	8	103.3	scaffold72.0	197,085
Pyd08_017	SNP	8	103.4	scaffold72.0	111,016
Pyb08_329	SNP	8	103.6	scaffold288.2	19,070
Pyb08_330	SNP	8	103.6	scaffold288.2	7,308
Pybd08_025	SNP	8	104.0	scaffold288.2	30,414
Pyb08_335	SNP	8	104.1	scaffold131.0	46,617
Pyb08_336	SNP	8	104.2	scaffold131.0	658,769
Pyb08_337	SNP	8	104.2	scaffold131.0	377,773
Pybd08_026	SNP	8	104.2	scaffold131.0	580,398
Pyb08_341	SNP	8	104.3	scaffold131.0	374,874
Pyb08_345	SNP	8	104.4	scaffold131.0	351,701
Pyb08_346	SNP	8	104.4	scaffold131.0	492,247
Pyb08_350	SNP	8	104.5	scaffold131.0	320,168

Pyb08_353	SNP	8	104.5	scaffold131.0	108,750
Pyb08_361	SNP	8	104.7	scaffold131.0	590,822
Pyb08_365	SNP	8	105.3	scaffold72.0	40,067
Pyb08_366	SNP	8	105.3	scaffold72.0	550,356
Pyb08_367	SNP	8	105.3	scaffold131.0	755,432
Pyb08_368	SNP	8	105.4	scaffold131.0	743,940
Pyb08_370	SNP	8	105.5	scaffold288.1	74,214
Pyb08_371	SNP	8	105.5	scaffold72.0	660,399
Pyb08_373	SNP	8	105.5	scaffold131.0	761,264
Pyb08_374	SNP	8	105.9	scaffold72.0	578,201
Pyb08_376	SNP	8	106.2	scaffold72.0	558,684
Pyd08_021	SNP	8	106.7	scaffold49.2	175,088
Pyd08_022	SNP	8	106.8	scaffold49.2	63,863
Pybd08_027	SNP	8	108.3	scaffold398.0	240,651
Pybd08_028	SNP	8	117.7	scaffold72.0	755,448
Pyb09_001	SNP	9	0.0	scaffold618.0	264,570
Pyb09_003	SNP	9	0.1	scaffold618.0	85,614
Pyb09_004	SNP	9	0.2	scaffold151.0	511,675
Pyb09_007	SNP	9	0.6	scaffold105.0	850,825
Pyb09_009	SNP	9	0.6	scaffold105.0	854,018
Pyb09_010	SNP	9	1.0	scaffold105.0	659,994
Pyb09_011	SNP	9	1.2	scaffold151.0	623,223
Pyb09_012	SNP	9	1.5	scaffold105.0	257,965
Pyb09_013	SNP	9	1.5	scaffold151.0	674,917
Pyb09_014	SNP	9	2.5	scaffold59.0	171,517
Pyb09_015	SNP	9	2.5	scaffold246.0	366,409
Pyb09_016	SNP	9	2.7	scaffold246.0	459,199
Pyb09_017	SNP	9	3.2	scaffold59.0	176,973
Pyb09_018	SNP	9	3.2	scaffold59.0	178,566
CO898678	SSR	9	3.6	scaffold59.0	135,823-136,059
Pyb09_023	SNP	9	4.2	scaffold59.0	338,210
Pyb09_024	SNP	9	4.3	scaffold59.0	326,492
Pyb09_027	SNP	9	8.1	scaffold55.0	194,432
Pyb09_028	SNP	9	8.9	scaffold55.0	450,725
Pyb09_029	SNP	9	9.3	scaffold725.0	30,999
Pyb09_032	SNP	9	10.6	scaffold127.0	665,118
Pyb09_034	SNP	9	11.0	scaffold1120.0	6,218
Pyb09_037	SNP	9	11.3	scaffold1342.0	45,426
Pyb09_039	SNP	9	11.5	scaffold397.0	114,393
Pyb09_043	SNP	9	11.7	scaffold368.0	95,263
Pyb09_046	SNP	9	11.9	scaffold1039.0	25,543
Pyb09_050	SNP	9	12.4	scaffold127.0	484,409
Pyb09_051	SNP	9	12.4	scaffold1039.0	19,797
Pyb09_053	SNP	9	12.5	scaffold127.0	206,326

CH05a03	SSR	9	12.6	scaffold127.0	644,703-644,906
Pyb09_057	SNP	9	12.7	scaffold127.0	10,837
Pyb09_058	SNP	9	12.8	scaffold397.0	49,063
Pyb09_061	SNP	9	13.4	scaffold55.0	495,352
Pyb09_062	SNP	9	13.8	scaffold55.0	208,115
Pyd09_002	SNP	9	20.7	scaffold618.0	145,652
Pyd09_009	SNP	9	26.1	scaffold246.0	436,847
Pyd09_012	SNP	9	28.9	scaffold213.0	483,043
Pyd09_015	SNP	9	33.5	scaffold29.0	301,979
Pyd09_017	SNP	9	33.9	scaffold29.0	446,294
Pyd09_022	SNP	9	37.3	scaffold225.0	185,059
Pybd09_002	SNP	9	42.6	scaffold354.0	342,431
Pyd09_025	SNP	9	42.7	scaffold486.0	134,677
Pyd09_026	SNP	9	42.8	scaffold486.0	136,368
Pyd09_028	SNP	9	42.9	scaffold486.0	179,108
NB106a	SSR	9	43.1	scaffold486.0	271,706-271,825
Pyb09_068	SNP	9	43.2	scaffold50.0	168,538
Pybd09_003	SNP	9	43.4	scaffold375.0	348,658
Pyd09_029	SNP	9	43.5	scaffold354.0	405,158
Pyd09_030	SNP	9	43.5	scaffold354.0	273,184
Pybd09_004	SNP	9	44.6	scaffold375.0	330,579
Pyb09_070	SNP	9	46.0	scaffold29.0	605,721
Pyd09_033	SNP	9	46.1	scaffold375.0	337,116
Pyb09_071	SNP	9	46.2	scaffold128.0	293,658
Pyb09_074	SNP	9	46.4	scaffold29.0	602,919
Pyb09_075	SNP	9	46.5	scaffold29.0	678,398
Pyd09_036	SNP	9	46.6	scaffold50.0	895,515
Pyb09_078	SNP	9	46.9	scaffold657.0	122,061
Pyb09_089	SNP	9	48.7	scaffold225.0	589,076
Pyb09_093	SNP	9	49.1	scaffold225.0	92,205
Pyb09_096	SNP	9	49.3	scaffold225.0	234,198
Pyb09_098	SNP	9	49.3	scaffold155.0	402,959
Pyb09_100	SNP	9	49.6	scaffold486.0	152,335
Pyb09_102	SNP	9	49.7	scaffold486.0	293,414
Pyb09_104	SNP	9	49.8	scaffold273.0	60,427
Pyb09_105	SNP	9	49.8	scaffold225.0	8,331
Pyb09_109	SNP	9	50.3	scaffold225.0	106,058
Pyb09_111	SNP	9	50.6	scaffold452.0	277,933
Pyb09_112	SNP	9	50.6	scaffold486.0	169,365
Pyb09_115	SNP	9	50.7	scaffold273.0	92,990
Pyb09_116	SNP	9	50.7	scaffold452.0	180,419
Pyb09_120	SNP	9	53.2	scaffold50.0	447,853
Pyb09_125	SNP	9	56.2	scaffold29.0	1,202,639
Pyb09_126	SNP	9	56.3	scaffold29.0	941,534

Pyb09_127	SNP	9	56.4	scaffold29.0	1,240,523
Pyb09_129	SNP	9	57.4	scaffold65.0	576,696
Pyb09_132	SNP	9	59.8	scaffold704.0	197,548
Pyb09_134	SNP	9	59.8	scaffold93.0	850,065
Hi04a05	SSR	9	60.0	scaffold704.0	196,872-197,099
Pybd09_005	SNP	9	61.3	scaffold375.0	239,877
Pyb09_137	SNP	9	62.5	scaffold375.0	342,303
Pyb09_138	SNP	9	62.6	scaffold50.0	828,755
KA20	SSR	9	62.9	scaffold1284.0	43,035-43,497
Pybd09_007	SNP	9	63.1	scaffold50.0	674,621
Pyb09_140	SNP	9	63.3	scaffold354.0	387,634
Pyb09_144	SNP	9	63.3	scaffold1284.0	11,347
Pyb09_148	SNP	9	63.4	scaffold354.0	309,830
Pyb09_150	SNP	9	63.4	scaffold1284.0	32,679
Pyb09_151	SNP	9	63.4	scaffold50.0	679,259
Pyb09_153	SNP	9	63.7	scaffold50.0	612,458
Pyb09_154	SNP	9	63.7	scaffold50.0	507,856
Pyb09_156	SNP	9	63.7	scaffold50.0	503,245
Pyb09_158	SNP	9	63.7	scaffold50.0	570,465
Pyd09_038	SNP	9	64.0	scaffold50.0	519,245
Pyd09_039	SNP	9	64.0	scaffold50.0	541,439
Pyd09_041	SNP	9	64.0	scaffold50.0	847,955
Pyd09_043	SNP	9	64.0	scaffold375.0	363,147
Pyd09_044	SNP	9	64.2	scaffold375.0	319,857
Pyd09_045	SNP	9	64.5	scaffold354.0	449,632
Pyd09_046	SNP	9	64.5	scaffold375.0	241,742
Pyd09_047	SNP	9	64.5	scaffold375.0	379,430
Pyd09_048	SNP	9	64.5	scaffold375.0	430,074
Pyb09_162	SNP	9	64.6	scaffold126.0	692,052
Pyb09_168	SNP	9	67.9	scaffold27.0	486,066
Pyb09_171	SNP	9	68.3	scaffold126.0	661,083
Pyb09_173	SNP	9	68.3	scaffold27.0	670,192
Pyb09_174	SNP	9	68.3	scaffold126.0	711,095
Pyb09_175	SNP	9	68.3	scaffold27.0	729,195
Pyb09_177	SNP	9	68.4	scaffold27.0	738,158
Pyb09_184	SNP	9	68.5	scaffold27.0	659,900
Pyd09_057	SNP	9	69.9	scaffold126.0	710,701
Pyd09_059	SNP	9	69.9	scaffold126.0	601,536
Pyd09_066	SNP	9	74.8	scaffold954.0	124,249
Pyd09_069	SNP	9	75.0	scaffold954.0	131,910
Pyd09_070	SNP	9	75.2	scaffold27.0	710,460
Pyd09_072	SNP	9	75.7	scaffold27.0	502,551
Pyb09_187	SNP	9	76.9	scaffold266.0	513,647
Pyb09_191	SNP	9	77.5	scaffold266.0	512,860

Pyb09_192	SNP	9	77.5	scaffold50.0	123,721
Pyb09_195	SNP	9	78.4	scaffold126.0	611,029
Pyb09_201	SNP	9	79.4	scaffold133.0	134,538
Pyb09_202	SNP	9	79.5	scaffold53.0	601,646
Pyb09_203	SNP	9	79.7	scaffold53.0	581,167
Pyb09_207	SNP	9	79.8	scaffold80.0	258,038
Pyb09_208	SNP	9	79.8	scaffold53.0	545,582
Pyb09_209	SNP	9	79.8	scaffold53.0	488,822
Pyb09_211	SNP	9	79.9	scaffold334.0	157,297
Pyb09_212	SNP	9	79.9	scaffold334.0	278,510
Pyb09_216	SNP	9	80.4	scaffold53.0	794,008
Pyb09_217	SNP	9	80.4	scaffold53.0	822,536
Pyb09_223	SNP	9	81.0	scaffold53.0	947,632
Pyb09_224	SNP	9	81.0	scaffold53.0	1,022,440
Pyb09_225	SNP	9	81.0	scaffold27.0	81,345
Pyb09_228	SNP	9	81.4	scaffold27.0	180,222
Pyb09_235	SNP	9	82.6	scaffold1220.0	79,778
Pyb09_236	SNP	9	82.8	scaffold27.0	767,602
Pyb09_237	SNP	9	83.1	scaffold126.0	314,846
Pyb09_240	SNP	9	83.8	scaffold685.0	214,374
Pyd09_074	SNP	9	91.0	scaffold422.0	94,797
Pybd09_011	SNP	9	101.6	scaffold382.0	326,381
Pyb09_254	SNP	9	109.1	scaffold422.0	94,778
Pyb09_255	SNP	9	109.3	scaffold817.0	162,203
Pyb09_256	SNP	9	109.4	scaffold382.0	313,979
Pyb09_257	SNP	9	109.5	scaffold382.0	350,105
Pybd09_014	SNP	9	109.7	scaffold382.0	206,156
Pyb09_267	SNP	9	110.5	scaffold817.0	159,858
Pyb09_268	SNP	9	110.6	scaffold940.0	7,605
Pyb09_269	SNP	9	110.6	scaffold940.0	100,283
Pyb09_272	SNP	9	110.8	scaffold292.0	189,524
Pyb09_274	SNP	9	111.0	scaffold382.0	88,034
Pyb09_275	SNP	9	111.1	scaffold677.0	29,644
Pyb09_280	SNP	9	111.3	scaffold940.0	4,843
Pyb09_281	SNP	9	111.3	scaffold940.0	42,727
Pyb09_283	SNP	9	111.3	scaffold940.0	67,736
Pyb09_286	SNP	9	111.3	scaffold940.0	52,859
Pyb09_288	SNP	9	111.4	scaffold70.0	460,389
Pyb09_291	SNP	9	111.7	scaffold940.0	52,845
Pyb09_292	SNP	9	111.7	scaffold422.0	49,553
Pyb09_299	SNP	9	111.8	scaffold382.0	256,941
Pyb09_300	SNP	9	111.8	scaffold292.0	204,980
Pyb09_301	SNP	9	112.0	scaffold940.0	87,867
Pyb09_302	SNP	9	112.0	scaffold817.0	13,366

Pyb09_304	SNP	9	112.2	scaffold382.0	289,107
Pyb09_306	SNP	9	112.6	scaffold817.0	94,528
Pyb09_307	SNP	9	112.6	scaffold214.0	72,716
Pyb09_308	SNP	9	112.9	scaffold817.0	162,266
Pyb09_309	SNP	9	113.2	scaffold292.0	169,954
Pyb09_310	SNP	9	113.3	scaffold292.0	144,885
Pyb09_311	SNP	9	113.4	scaffold292.0	15,044
Pyb09_314	SNP	9	114.7	scaffold53.0	476,743
Pyb09_315	SNP	9	114.8	scaffold133.0	75,019
Pyb09_318	SNP	9	115.0	scaffold334.0	190,025
Pyb09_319	SNP	9	115.1	scaffold334.0	169,748
Pyd09_085	SNP	9	115.7	scaffold50.0	149,371
Pyb09_323	SNP	9	115.9	scaffold53.0	881,848
Pyb09_325	SNP	9	116.6	scaffold27.0	79,144
Pyb09_326	SNP	9	116.7	scaffold53.0	640,132
Pyb09_328	SNP	9	119.0	scaffold53.0	635,286
Pybd10_001	SNP	10	0.0	scaffold568.0	130,242
Pyb10_001	SNP	10	1.9	scaffold169.0	350,245
Pyb10_003	SNP	10	2.0	scaffold1206.0	16,412
Pyb10_005	SNP	10	2.3	scaffold1302.0	47,373
Pyb10_012	SNP	10	3.0	scaffold169.0	292,597
Pyb10_013	SNP	10	3.1	scaffold169.0	344,150
Pyb10_014	SNP	10	3.1	scaffold169.0	149,078
Pyb10_015	SNP	10	3.1	scaffold169.0	376,189
Pyb10_016	SNP	10	3.1	scaffold32.0	930,312
Pyb10_017	SNP	10	3.1	scaffold169.0	400,280
Pyb10_020	SNP	10	3.5	scaffold552.0	208,133
Pyb10_022	SNP	10	5.0	scaffold1036.0	2,195
Pyb10_023	SNP	10	5.2	scaffold32.0	915,693
Pyb10_024	SNP	10	5.2	scaffold32.0	520,197
Pyb10_026	SNP	10	5.3	scaffold32.0	546,661
Pyb10_027	SNP	10	5.3	scaffold32.0	802,948
Pyb10_032	SNP	10	6.3	scaffold32.0	382,088
Pyb10_033	SNP	10	6.3	scaffold32.0	429,298
Pyb10_034	SNP	10	6.7	scaffold32.0	301,164
Pyb10_037	SNP	10	6.9	scaffold32.0	338,898
Pyb10_038	SNP	10	7.0	scaffold32.0	273,417
Pyb10_039	SNP	10	7.0	scaffold32.0	201,257
Pyb10_042	SNP	10	7.3	scaffold32.0	280,676
Pyb10_044	SNP	10	7.7	scaffold32.0	225,891
Pyb10_045	SNP	10	7.8	scaffold32.0	259,150
Pyb10_046	SNP	10	7.9	scaffold32.0	198,542
Pyb10_049	SNP	10	10.1	scaffold761.0	79,842
Pyb10_051	SNP	10	10.8	scaffold761.0	147,583

Pyb10_054	SNP	10	12.3	scaffold85.0	478,191
Pyb10_057	SNP	10	12.5	scaffold85.0	683,062
Pyb10_060	SNP	10	12.8	scaffold24.0	218,340
Pyb10_061	SNP	10	12.8	scaffold24.0	161,304
Pyb10_063	SNP	10	12.9	scaffold85.0	633,231
Pyb10_064	SNP	10	12.9	scaffold24.0	133,362
Pyb10_065	SNP	10	12.9	scaffold314.0	16,550
Pyb10_069	SNP	10	12.9	scaffold85.0	845,750
Pyb10_070	SNP	10	13.0	scaffold24.0	49,908
Pybd10_002	SNP	10	13.1	scaffold24.0	308,527
Pybd10_003	SNP	10	13.2	scaffold24.0	315,098
Pyb10_072	SNP	10	13.7	scaffold24.0	298,594
Pyb10_073	SNP	10	13.7	scaffold24.0	431,646
Pyb10_074	SNP	10	13.7	scaffold24.0	1,229,453
Pyb10_075	SNP	10	13.7	scaffold24.0	969,949
Pyb10_076	SNP	10	13.7	scaffold24.0	423,813
CH02b03b	SSR	10	13.9	scaffold24.0	497232-497087
Pyb10_078	SNP	10	13.9	scaffold24.0	957,437
Pyb10_079	SNP	10	13.9	scaffold24.0	1,152,794
Pyb10_081	SNP	10	14.0	scaffold24.0	662,513
Pyb10_082	SNP	10	14.0	scaffold24.0	1,106,996
Pyb10_083	SNP	10	14.0	scaffold24.0	1,400,484
Pyb10_087	SNP	10	14.1	scaffold85.0	434,765
Pyb10_090	SNP	10	14.3	scaffold24.0	640,476
Pyb10_100	SNP	10	14.8	scaffold1039.0	112,473
Pyb10_105	SNP	10	14.9	scaffold1039.0	118,634
Pyb10_112	SNP	10	15.3	scaffold24.0	949,175
Pyb10_114	SNP	10	15.4	scaffold24.0	1,030,058
Pyb10_117	SNP	10	15.5	scaffold24.0	898,805
Pyb10_125	SNP	10	16.6	scaffold57.0	1,157,119
Pyb10_127	SNP	10	16.7	scaffold57.0	340,575
MS06g03	SSR	10	16.9	scaffold57.0	882,928-883,157
Pyb10_128	SNP	10	17.3	scaffold261.0	3,319
Pyb10_129	SNP	10	18.0	scaffold261.0	351,082
Pyd10_004	SNP	10	28.1	scaffold169.0	149,011
Pyd10_011	SNP	10	29.9	scaffold810.0	103,052
Pyb10_133	SNP	10	30.1	scaffold24.0	261,104
Pyb10_134	SNP	10	30.5	scaffold57.0	1,013,202
Pybd10_004	SNP	10	31.6	scaffold57.0	580,646
Pyb10_139	SNP	10	35.5	scaffold261.0	177,004
Pyb10_140	SNP	10	35.5	scaffold57.0	767,650
Pyb10_141	SNP	10	35.7	scaffold261.0	107,714
Pyb10_142	SNP	10	36.1	scaffold24.0	922,242
Pyb10_143	SNP	10	36.2	scaffold24.0	429,669

Pyb10_144	SNP	10	36.4	scaffold57.0	587,420
Pyb10_145	SNP	10	36.4	scaffold261.0	37,959
Pyb10_146	SNP	10	36.4	scaffold57.0	518,689
Pyd10_017	SNP	10	36.9	scaffold24.0	431,630
Pyb10_152	SNP	10	37.2	scaffold24.0	1,032,168
Pyb10_154	SNP	10	37.2	scaffold24.0	408,674
Pyb10_156	SNP	10	37.2	scaffold24.0	1,164,381
Pyb10_157	SNP	10	37.2	scaffold24.0	959,884
Pyb10_160	SNP	10	37.4	scaffold24.0	787,261
Pyb10_163	SNP	10	37.8	scaffold85.0	90,993
Pyb10_164	SNP	10	37.8	scaffold85.0	551,502
Pyb10_165	SNP	10	37.8	scaffold85.0	586,003
Pyb10_166	SNP	10	37.8	scaffold85.0	810,123
Pyb10_167	SNP	10	37.8	scaffold24.0	69,811
Pyb10_169	SNP	10	37.9	scaffold85.0	962,328
Pyb10_170	SNP	10	37.9	scaffold85.0	517,869
Pyb10_172	SNP	10	37.9	scaffold85.0	741,296
Pyb10_173	SNP	10	38.0	scaffold85.0	774,382
Pyb10_174	SNP	10	38.0	scaffold85.0	651,994
Pyb10_175	SNP	10	38.0	scaffold85.0	517,936
Pyb10_177	SNP	10	38.2	scaffold85.0	805,236
Pyb10_183	SNP	10	39.1	scaffold57.0	625,725
Pyb10_185	SNP	10	39.1	scaffold57.0	1,036,318
Pyb10_186	SNP	10	39.1	scaffold57.0	612,911
Pyb10_187	SNP	10	39.2	scaffold261.0	43,704
Pyb10_188	SNP	10	39.2	scaffold57.0	819,766
Pyb10_189	SNP	10	39.2	scaffold57.0	553,370
Pyb10_190	SNP	10	39.2	scaffold261.0	326,352
Pyb10_193	SNP	10	39.3	scaffold261.0	13,320
Pyb10_199	SNP	10	39.7	scaffold261.0	357,846
Pyb10_200	SNP	10	39.8	scaffold57.0	553,323
Pyb10_202	SNP	10	39.9	scaffold261.0	63,764
Pyb10_203	SNP	10	39.9	scaffold261.0	63,752
Pyd10_020	SNP	10	50.5	scaffold1302.0	47,319
Pyd10_021	SNP	10	50.5	scaffold1206.0	39,172
Pybd10_007	SNP	10	52.4	scaffold619.0	128,348
Pyb10_210	SNP	10	52.5	scaffold157.0	744,140
Pyb10_213	SNP	10	52.7	scaffold810.0	49,233
Pyd10_022	SNP	10	52.7	scaffold177.0	211,601
Pyb10_214	SNP	10	52.7	scaffold810.0	160,585
Pyb10_216	SNP	10	52.8	scaffold810.0	135,700
CH03d11	SSR	10	53.0	scaffold177.0	204,200-203,936
Pyd10_025	SNP	10	55.4	scaffold420.0	55,065
Pyd10_026	SNP	10	55.4	scaffold403.0	286,242

Pyd10_027	SNP	10	55.5	scaffold220.0	281,817
Pyb10_224	SNP	10	55.6	scaffold177.0	385,946
Pyb10_227	SNP	10	55.8	scaffold177.0	122,104
Pyb10_228	SNP	10	55.8	scaffold157.0	565,502
Pyb10_229	SNP	10	55.9	scaffold157.0	511,773
Pyb10_230	SNP	10	56.0	scaffold177.0	295,185
Pyb10_231	SNP	10	56.0	scaffold177.0	283,826
Pyb10_233	SNP	10	56.1	scaffold157.0	371,865
Pyb10_234	SNP	10	56.1	scaffold157.0	516,224
BGT24	SSR	10	56.2	scaffold157.0	229,119-229,348
Pyb10_236	SNP	10	56.3	scaffold157.0	555,880
Pyb10_239	SNP	10	56.6	scaffold157.0	256,658
Pyb10_241	SNP	10	56.9	scaffold177.0	253,434
Pyb10_248	SNP	10	58.4	scaffold177.0	24,754
Pyb10_253	SNP	10	59.6	scaffold177.0	111,332
Pyb10_256	SNP	10	62.9	scaffold420.0	92,850
Pyb10_260	SNP	10	63.6	scaffold403.0	306,980
Pyb10_261	SNP	10	63.6	scaffold403.0	285,073
Pyb10_262	SNP	10	63.7	scaffold403.0	282,798
Pyb10_265	SNP	10	64.0	scaffold220.0	574,520
Pyb10_266	SNP	10	64.1	scaffold403.0	392,049
Pybd10_008	SNP	10	64.8	scaffold560.0	221,815
CH02c11	SSR	10	65.0	scaffold220.0	189,073-189,299
Pyb10_271	SNP	10	66.4	scaffold377.0	69,679
Pybd10_010	SNP	10	66.5	scaffold220.0	574,568
Pyb10_272	SNP	10	66.6	scaffold220.0	549,847
Pyb10_274	SNP	10	67.2	scaffold377.0	89,199
NH045a	SSR	10	67.4	scaffold377.0	153,709-154,378
Pybd10_011	SNP	10	72.0	scaffold220.0	311,626
Pyb10_277	SNP	10	79.7	scaffold562.0	121,441
Pyb10_281	SNP	10	80.3	scaffold308.0	106,100
Pyb10_284	SNP	10	80.8	scaffold562.0	134,138
Pybd10_012	SNP	10	82.6	scaffold881.0	44,898
CH01f12	SSR	10	82.8	scaffold881.0	117,385-117,531
CTG1066085-	SSR	10	83.0	Not_anchored	-
Pybd10_013	SNP	10	83.0	scaffold62.0	67,361
Pyd10_042	SNP	10	84.7	scaffold423.0	126,744
Pybd10_014	SNP	10	86.3	scaffold650.0	89,917
AU223670	SSR	10	86.5	scaffold650.0	192,966-193,162
Pybd10_017	SNP	10	88.8	scaffold1156.0	5,644
Pyb10_289	SNP	10	91.6	scaffold34.0	1,313,358
Pyb10_291	SNP	10	91.7	scaffold298.0	106,091
Pyb10_292	SNP	10	91.8	scaffold189.0	291,953
Pyb10_293	SNP	10	91.9	scaffold521.0	283,634

Pyb10_294	SNP	10	91.9	scaffold521.0	304,505
Pyb10_298	SNP	10	92.3	scaffold282.0	359,862
Pyb10_299	SNP	10	92.4	scaffold962.0	130,123
NH039a	SSR	10	92.5	scaffold282.0	484,551-484,678
Pyb10_303	SNP	10	92.6	scaffold408.0	165,717
Pyb10_305	SNP	10	92.6	scaffold408.0	101,938
Pyb10_307	SNP	10	92.8	scaffold176.0	132,191
Pyb10_308	SNP	10	92.8	scaffold189.0	313,733
Pyb10_315	SNP	10	93.5	scaffold321.0	461,137
Pyb10_327	SNP	10	94.3	scaffold308.0	120,536
Pyb10_329	SNP	10	94.4	scaffold562.0	15,956
Pyb10_331	SNP	10	94.4	scaffold308.0	475,102
Pyb10_332	SNP	10	94.5	scaffold606.0	111,795
Pyb10_333	SNP	10	94.5	scaffold308.0	102,775
Pyb10_334	SNP	10	94.5	scaffold308.0	20,460
Pyb10_341	SNP	10	94.9	scaffold562.0	23,970
Pyb10_342	SNP	10	94.9	scaffold562.0	121,423
Pyb10_347	SNP	10	95.6	scaffold606.0	242,621
Pyb10_352	SNP	10	96.1	scaffold220.0	271,996
Pyb10_361	SNP	10	102.9	scaffold338.0	366,419
Pyb10_367	SNP	10	103.3	scaffold192.0	30,377
Pyb10_371	SNP	10	103.4	scaffold164.0	321,899
Pyb10_374	SNP	10	103.4	scaffold192.0	96,598
Pyb10_375	SNP	10	103.5	scaffold338.0	177,493
Pyb10_381	SNP	10	106.8	scaffold163.0	161,950
Pyb10_384	SNP	10	107.1	scaffold84.0	898,461
Pyb10_385	SNP	10	107.1	scaffold296.0	17,935
Pyb10_389	SNP	10	108.1	scaffold84.0	221,728
Pyb10_393	SNP	10	108.2	scaffold985.0	101,886
Pyb10_394	SNP	10	108.2	scaffold196.0	370,351
Pyb10_395	SNP	10	108.2	scaffold84.0	308,576
Pyb10_402	SNP	10	108.4	scaffold84.0	281,042
Pyb10_403	SNP	10	108.4	scaffold84.0	426,477
Pybd10_027	SNP	10	112.2	scaffold232.0	213,084
Pyb10_406	SNP	10	113.1	scaffold196.0	349,110
Pyb10_410	SNP	10	117.7	scaffold192.0	45,681
Pyb10_413	SNP	10	118.9	scaffold163.0	234,085
Pybd10_029	SNP	10	122.2	scaffold84.0	697,685
Pyb10_423	SNP	10	126.1	scaffold164.0	132,442
Pyb10_429	SNP	10	126.6	scaffold164.0	216,492
Pyb10_430	SNP	10	126.6	scaffold164.0	55,067
Pyb10_431	SNP	10	126.6	scaffold164.0	132,363
Pyb10_433	SNP	10	126.8	scaffold84.0	942,492
Pyb10_435	SNP	10	126.9	scaffold84.0	763,137

Pyb10_438	SNP	10	127.0	scaffold84.0	772,932
Pyb10_439	SNP	10	127.0	scaffold715.0	101,923
Pyb10_440	SNP	10	127.0	scaffold296.0	128,212
Pyb10_452	SNP	10	127.4	scaffold874.0	69,756
Pyb10_456	SNP	10	127.4	scaffold271.0	11,815
Pyb10_460	SNP	10	127.5	scaffold443.0	65,941
Pyb10_469	SNP	10	127.7	scaffold443.0	319,882
Pyb10_470	SNP	10	127.7	scaffold667.0	55,130
Pyb10_474	SNP	10	128.1	scaffold196.0	376,415
Pyb10_480	SNP	10	128.7	scaffold164.0	153,029
Pyb10_481	SNP	10	128.8	scaffold164.0	43,135
Pyb10_482	SNP	10	129.0	scaffold164.0	93,396
Pyb10_486	SNP	10	129.8	scaffold256.0	390,099
Pyb10_488	SNP	10	129.9	scaffold192.0	175,066
Pyb10_489	SNP	10	129.9	scaffold338.0	341,739
Pyb10_492	SNP	10	129.9	scaffold338.0	262,523
Pyb10_493	SNP	10	129.9	scaffold338.0	415,806
Pyb10_496	SNP	10	130.0	scaffold862.0	74,175
Pyb10_500	SNP	10	130.3	scaffold976.0	71,363
Pyb10_506	SNP	10	130.9	scaffold256.0	312,274
Pyd10_054	SNP	10	138.6	scaffold164.0	238,999
Pybd10_033	SNP	10	141.9	scaffold232.0	131,094
CH02a08	SSR	10	142.1	scaffold232.0	408,616-408,303
Pyd11_001	SNP	11	0.0	scaffold538.0	171,962
Pyb11_003	SNP	11	0.9	scaffold8.0	498,964
Pyb11_004	SNP	11	0.9	scaffold8.0	481,189
Pybd11_004	SNP	11	3.1	scaffold1138.0	2,362
CH04g07	SSR	11	3.5	scaffold679.0	19,235-19,401
Pyb11_006	SNP	11	3.5	scaffold1138.0	101,172
Pyd11_005	SNP	11	3.9	scaffold26.0	119,238
Pyb11_012	SNP	11	4.3	scaffold538.0	303,354
Pyb11_013	SNP	11	4.3	scaffold538.0	76,690
Pyb11_014	SNP	11	4.4	scaffold538.0	68,782
Pybd11_005	SNP	11	5.4	scaffold538.0	303,904
Pyb11_024	SNP	11	5.4	scaffold538.0	58,299
Pyb11_025	SNP	11	5.4	scaffold961.0	103,268
Pyb11_027	SNP	11	5.4	scaffold961.0	56,521
Pyb11_028	SNP	11	5.4	scaffold961.0	141,993
Pyb11_029	SNP	11	5.4	scaffold538.0	10,641
Pyb11_031	SNP	11	5.5	scaffold156.0	206,224
Pyb11_032	SNP	11	5.5	scaffold538.0	226,358
Pyb11_034	SNP	11	5.5	scaffold926.0	150,684
Pyb11_035	SNP	11	5.5	scaffold538.0	91,741
Pyb11_036	SNP	11	5.5	scaffold538.0	189,813

Pyb11_038	SNP	11	5.9	scaffold26.0	26,367
Pyb11_039	SNP	11	6.1	scaffold538.0	13,122
Pyb11_041	SNP	11	6.1	scaffold165.1	41,988
Pyb11_042	SNP	11	6.1	scaffold165.2	236,461
Pybd11_006	SNP	11	6.2	scaffold26.0	265,475
Pyd11_006	SNP	11	6.3	scaffold165.1	67,609
Pyb11_051	SNP	11	6.5	scaffold156.0	225,304
Pyb11_052	SNP	11	6.5	scaffold301.0	171,596
Pyb11_058	SNP	11	6.5	scaffold26.0	241,707
Pyb11_060	SNP	11	6.5	scaffold165.2	89,675
Pyb11_061	SNP	11	6.5	scaffold156.0	4,393
Pyb11_062	SNP	11	6.5	scaffold156.0	72,957
Pyb11_063	SNP	11	6.5	scaffold301.0	131,104
Pyb11_064	SNP	11	6.5	scaffold26.0	227,537
Pyb11_065	SNP	11	6.5	scaffold165.2	227,733
Pyb11_066	SNP	11	6.5	scaffold301.0	250,256
Pyb11_067	SNP	11	6.5	scaffold165.2	128,166
Pyb11_070	SNP	11	6.5	scaffold156.0	130,725
Pyb11_071	SNP	11	6.5	scaffold26.0	331,586
Pyb11_072	SNP	11	6.5	scaffold301.0	256,846
Pyb11_073	SNP	11	6.5	scaffold156.0	13,555
Pyb11_074	SNP	11	6.5	scaffold165.2	284,661
Pyb11_076	SNP	11	6.6	scaffold156.0	229,434
Pyb11_079	SNP	11	6.6	scaffold165.2	207,815
Pyb11_082	SNP	11	7.0	scaffold156.0	26,662
Pyb11_083	SNP	11	7.6	scaffold156.0	312,363
Pyb11_084	SNP	11	7.6	scaffold8.0	668,040
Pyb11_085	SNP	11	7.6	scaffold679.0	150,860
Pyb11_086	SNP	11	7.6	scaffold8.0	735,201
Pyb11_087	SNP	11	7.6	scaffold679.0	37,222
Pyb11_088	SNP	11	7.6	scaffold679.0	26,162
Pyb11_089	SNP	11	7.6	scaffold8.0	898,309
Pyb11_091	SNP	11	7.6	scaffold8.0	1,064,183
Pyb11_094	SNP	11	7.6	scaffold8.0	1,080,474
Pyb11_099	SNP	11	7.7	scaffold8.0	934,325
Pyb11_103	SNP	11	8.0	scaffold165.2	266,475
Pyb11_105	SNP	11	8.3	scaffold8.0	1,081,352
Pyb11_106	SNP	11	8.4	scaffold8.0	629,175
Pyb11_107	SNP	11	8.4	scaffold679.0	89,105
Pyb11_108	SNP	11	8.4	scaffold8.0	1,312,012
Pyb11_113	SNP	11	9.2	scaffold8.0	762,756
Pyb11_115	SNP	11	10.7	scaffold8.0	1,725,612
Pyb11_116	SNP	11	10.8	scaffold8.0	1,732,062
Pyb11_118	SNP	11	11.6	scaffold337.0	193,995

Pyb11_119	SNP	11	11.9	scaffold603.0	12,651
Pyb11_121	SNP	11	12.2	scaffold603.0	35,199
Pybd11_007	SNP	11	12.5	scaffold1246.0	42,800
Pyb11_124	SNP	11	12.7	scaffold45.0	622,714
Pyb11_125	SNP	11	12.9	scaffold45.0	688,643
Pyb11_126	SNP	11	13.0	scaffold337.0	334,174
Pyb11_127	SNP	11	13.0	scaffold337.0	99,508
Pybd11_008	SNP	11	13.9	scaffold337.0	270,900
Pyb11_129	SNP	11	14.7	scaffold12.0	585,921
Pyb11_130	SNP	11	16.1	scaffold229.0	155,435
Pyb11_131	SNP	11	16.1	scaffold45.0	14,327
Pybd11_010	SNP	11	16.4	scaffold229.0	157,783
Pyb11_132	SNP	11	24.8	scaffold1552.0	22,705
Pyb11_140	SNP	11	27.4	scaffold156.0	244,583
Pyb11_142	SNP	11	30.0	scaffold45.0	579,361
Pyb11_144	SNP	11	31.0	scaffold337.0	407,947
Pyb11_146	SNP	11	31.0	scaffold45.0	584,264
Pyb11_149	SNP	11	31.1	scaffold1552.0	3,339
Pyb11_151	SNP	11	31.1	scaffold603.0	71,893
Pyb11_152	SNP	11	31.1	scaffold45.0	687,471
Pyb11_153	SNP	11	31.1	scaffold337.0	306,655
Pyb11_155	SNP	11	31.1	scaffold337.0	361,362
Pyb11_157	SNP	11	31.1	scaffold12.0	540,244
Pyb11_158	SNP	11	31.1	scaffold337.0	315,216
Pyb11_162	SNP	11	31.4	scaffold603.0	12,597
Pyb11_164	SNP	11	31.4	scaffold8.0	1,520,242
Pyb11_166	SNP	11	31.5	scaffold337.0	446,792
Pyb11_168	SNP	11	31.8	scaffold603.0	39,019
Pybd11_013	SNP	11	33.6	scaffold497.0	207,288
Pyb11_175	SNP	11	34.9	scaffold497.0	106,226
Pyb11_176	SNP	11	35.2	scaffold1070.0	56,049
Pyb11_179	SNP	11	36.1	scaffold383.0	225,973
Pyb11_181	SNP	11	36.1	scaffold383.0	161,712
IPPN14	SSR	11	36.8	scaffold1283.0	49,468-49,717
Pyb11_187	SNP	11	37.0	scaffold56.0	603,970
Pyb11_188	SNP	11	37.0	scaffold720.0	156,650
Pyb11_190	SNP	11	37.0	scaffold720.0	131,106
Pyb11_201	SNP	11	38.1	scaffold912.0	26,320
Pyb11_202	SNP	11	38.1	scaffold41.0	990,318
Pyb11_204	SNP	11	38.5	scaffold41.0	945,082
Pyb11_205	SNP	11	38.5	scaffold41.0	1,233,496
Pyb11_207	SNP	11	38.5	scaffold41.0	1,036,808
Pyb11_208	SNP	11	39.2	scaffold138.0	611,021
Pyb11_210	SNP	11	39.3	scaffold138.0	577,320

Pyb11_211	SNP	11	39.3	scaffold306.0	461,060
Pyb11_213	SNP	11	39.3	scaffold306.0	453,590
Pyb11_214	SNP	11	39.3	scaffold493.0	104,107
Pyb11_215	SNP	11	39.3	scaffold138.0	178,022
Pyb11_219	SNP	11	39.4	scaffold138.0	148,382
Pyb11_220	SNP	11	39.5	scaffold1288.0	4,163
Pyb11_224	SNP	11	39.8	scaffold267.0	512,128
Pyb11_230	SNP	11	40.4	scaffold1288.0	8,834
Pybd11_017	SNP	11	42.4	scaffold1519.0	20,186
Pybd11_019	SNP	11	44.8	scaffold138.0	583,142
Pybd11_021	SNP	11	46.2	scaffold41.0	1,248,274
Pybd11_023	SNP	11	49.7	scaffold138.0	634,673
Pyd11_017	SNP	11	53.3	scaffold56.0	518,761
Pyb11_240	SNP	11	62.1	scaffold847.0	136,913
CH05c02	SSR	11	67.0	scaffold5.0	865,900-866,080
Pyb11_247	SNP	11	68.4	scaffold892.0	146,068
Pyb11_250	SNP	11	68.6	scaffold892.0	147,622
Pyb11_251	SNP	11	68.9	scaffold847.0	63,300
Pyb11_253	SNP	11	69.1	scaffold1074.0	84,257
Pyb11_255	SNP	11	69.1	scaffold236.0	482,882
Pyb11_264	SNP	11	69.9	scaffold5.0	870,213
Pyb11_265	SNP	11	69.9	scaffold5.0	1,131,368
Pyb11_266	SNP	11	69.9	scaffold5.0	1,439,895
Pyb11_268	SNP	11	70.0	scaffold5.0	1,108,064
Pyb11_269	SNP	11	70.0	scaffold5.0	850,288
Pyb11_270	SNP	11	70.1	scaffold5.0	816,686
Pyb11_271	SNP	11	70.1	scaffold5.0	867,764
Pyb11_272	SNP	11	70.2	scaffold5.0	1,430,186
Pyb11_273	SNP	11	70.3	scaffold5.0	1,116,066
Pyb11_276	SNP	11	70.4	scaffold5.0	1,008,202
Pyb11_278	SNP	11	70.5	scaffold5.0	948,369
Pyb11_281	SNP	11	71.0	scaffold268.0	472,754
Pyb11_283	SNP	11	71.1	scaffold576.0	257,124
Pyb11_284	SNP	11	71.1	scaffold5.0	1,120,971
Pyb11_286	SNP	11	71.5	scaffold210.0	157,575
Pyb11_288	SNP	11	71.6	scaffold1016.0	85,925
Pyb11_290	SNP	11	71.7	scaffold518.0	32,817
Pyb11_292	SNP	11	71.9	scaffold909.0	33,619
NH005b	SSR	11	72.2	scaffold227.0	300,357-300,680
Pyb11_294	SNP	11	72.3	scaffold723.0	185,301
Pyb11_296	SNP	11	72.5	scaffold669.0	157,515
Pyb11_300	SNP	11	72.7	scaffold669.0	17,742
Pybd11_027	SNP	11	73.0	scaffold576.0	219,323
Pybd11_029	SNP	11	73.9	scaffold498.0	93,564

Pyb11_302	SNP	11	75.0	scaffold510.0	14,261
Pyb11_303	SNP	11	75.0	scaffold52.0	824,821
Pyb11_306	SNP	11	75.3	scaffold52.0	686,026
Pyb11_307	SNP	11	75.4	scaffold498.0	40,708
Pyb11_310	SNP	11	75.5	scaffold460.0	171,174
Pyb11_313	SNP	11	75.5	scaffold153.0	63,152
Pyb11_316	SNP	11	75.7	scaffold52.0	535,314
Pyb11_319	SNP	11	75.9	scaffold52.0	645,262
Pyb11_321	SNP	11	75.9	scaffold52.0	1,065,019
Pyb11_322	SNP	11	75.9	scaffold52.0	1,064,983
Pyb11_325	SNP	11	75.9	scaffold52.0	1,087,047
Pyb11_327	SNP	11	76.0	scaffold52.0	856,630
Pyb11_328	SNP	11	76.0	scaffold52.0	542,673
Pyb11_329	SNP	11	76.1	scaffold52.0	887,363
Pybd11_030	SNP	11	76.4	scaffold643.0	182,821
Pybd11_031	SNP	11	76.9	scaffold52.0	742,272
Pyb11_332	SNP	11	77.7	scaffold407.0	416,128
Pyb11_335	SNP	11	77.8	scaffold407.0	376,330
Pyb11_336	SNP	11	77.9	scaffold227.0	586,862
Pyb11_341	SNP	11	78.0	scaffold52.0	47,666
Pyb11_343	SNP	11	79.4	scaffold643.0	115,278
Pyb11_344	SNP	11	79.5	scaffold407.0	218,074
Pyb11_345	SNP	11	79.8	scaffold407.0	92,030
Pyb11_346	SNP	11	79.8	scaffold407.0	336,194
Pyb11_347	SNP	11	79.8	scaffold407.0	70,552
Pyb11_348	SNP	11	79.8	scaffold407.0	253,121
Pyb11_349	SNP	11	79.9	scaffold407.0	107,219
Pyb11_350	SNP	11	79.9	scaffold643.0	254,134
Pyb11_351	SNP	11	79.9	scaffold407.0	179,823
Pyb11_352	SNP	11	79.9	scaffold407.0	185,067
Pyb11_353	SNP	11	80.0	scaffold407.0	97,711
Pybd11_032	SNP	11	80.7	scaffold31.0	656,689
Pyb11_359	SNP	11	81.8	scaffold31.0	579,634
Pybd11_033	SNP	11	82.2	scaffold36.0	327,509
Pyb11_366	SNP	11	82.8	scaffold36.0	328,589
Pyb11_372	SNP	11	86.6	scaffold286.0	185,051
Pyb11_374	SNP	11	87.4	scaffold286.0	278,703
Pyd11_032	SNP	11	89.2	scaffold460.0	18,646
Pyb11_375	SNP	11	91.2	scaffold286.0	209,724
Pybd11_035	SNP	11	99.4	scaffold259.0	284,322
CH04h02	SSR	11	100.6	scaffold513.0	20,317-20,501
Pybd11_036	SNP	11	102.3	scaffold513.0	45,690
Pyb11_377	SNP	11	103.5	scaffold513.0	152,218
Pyb11_378	SNP	11	103.5	scaffold513.0	165,366

Pyb11_379	SNP	11	103.6	scaffold513.0	308,325
Pyb11_380	SNP	11	103.7	scaffold513.0	234,298
Pyb11_381	SNP	11	106.1	scaffold114.0	458,333
Pyb11_382	SNP	11	106.7	scaffold114.0	313,086
Pyb11_383	SNP	11	106.7	scaffold114.0	801,547
Pyb11_384	SNP	11	106.7	scaffold513.0	189,963
Pyb11_385	SNP	11	106.8	scaffold114.0	430,148
Pyb11_386	SNP	11	107.0	scaffold114.0	361,922
Pyb11_388	SNP	11	107.4	scaffold114.0	621,616
Pyb11_393	SNP	11	107.9	scaffold121.0	573,604
Pyb11_398	SNP	11	108.9	scaffold355.0	17,705
Pyb11_408	SNP	11	111.0	scaffold516.0	234,627
Pyb11_411	SNP	11	111.2	scaffold153.0	776,474
EMPc11	SSR	11	111.4	scaffold259.0	215,979-216,123
Pyb11_415	SNP	11	111.5	scaffold1137.0	27,051
Pyb11_416	SNP	11	111.6	scaffold516.0	247,572
Pyb11_418	SNP	11	111.8	scaffold259.0	454,208
Pybd11_039	SNP	11	111.9	scaffold259.0	284,360
Pyb11_421	SNP	11	113.4	scaffold259.0	221,458
Pyb11_422	SNP	11	113.5	scaffold259.0	384,602
Pyb11_423	SNP	11	113.5	scaffold259.0	323,525
Pyb11_425	SNP	11	114.4	scaffold259.0	174,289
Pyb11_428	SNP	11	115.5	scaffold181.0	502,758
Pyb11_429	SNP	11	115.5	scaffold181.0	509,637
Pyb11_430	SNP	11	115.5	scaffold699.0	208,965
Pyb11_432	SNP	11	115.6	scaffold181.0	75,914
Pyb11_434	SNP	11	115.7	scaffold622.0	94,421
Pyb11_435	SNP	11	115.8	scaffold181.0	509,607
Pybd11_040	SNP	11	115.9	scaffold699.0	93,338
Pyb11_441	SNP	11	116.2	scaffold181.0	90,245
Pyb11_442	SNP	11	116.5	scaffold181.0	494,186
Pyd11_052	SNP	11	117.9	scaffold259.0	431,251
Pyd11_053	SNP	11	117.9	scaffold931.0	133,915
Pyd11_054	SNP	11	117.9	scaffold259.0	376,712
Pyd11_055	SNP	11	117.9	scaffold259.0	211,830
Pybd11_041	SNP	11	118.4	scaffold181.0	491,061
Pyd11_057	SNP	11	119.0	scaffold622.0	165,512
Pyb11_456	SNP	11	120.9	scaffold461.0	295,871
Pyb11_459	SNP	11	123.0	scaffold286.0	316,534
Pyb11_461	SNP	11	123.3	scaffold286.0	309,146
Pyb11_462	SNP	11	123.3	scaffold286.0	319,378
Pyb11_463	SNP	11	123.3	scaffold286.0	310,274
Pyb11_467	SNP	11	123.3	scaffold286.0	364,478
Pyb11_468	SNP	11	123.7	scaffold1294.0	1,336

Pybd11_043	SNP	11	127.0	scaffold286.0	279,697
Pyb11_474	SNP	11	131.3	scaffold484.0	327,340
Pyb11_475	SNP	11	131.4	scaffold36.0	172,924
Pyb11_476	SNP	11	131.4	scaffold36.0	155,058
Pyb11_484	SNP	11	131.5	scaffold484.0	152,299
Pyb11_487	SNP	11	133.0	scaffold643.0	195,216
Pyb11_492	SNP	11	133.8	scaffold643.0	219,790
Pyb11_494	SNP	11	137.2	scaffold52.0	491,878
Pyb11_495	SNP	11	137.5	scaffold52.0	106,254
Pyb11_497	SNP	11	137.9	scaffold52.0	789,826
Pyb11_498	SNP	11	138.0	scaffold52.0	529,376
Pyb11_499	SNP	11	138.0	scaffold52.0	789,567
Pyb11_501	SNP	11	138.0	scaffold52.0	934,228
Pyb11_502	SNP	11	138.0	scaffold52.0	540,885
Pyb11_504	SNP	11	138.1	scaffold1106.0	105,940
Pyb11_509	SNP	11	138.1	scaffold52.0	556,025
Pybd12_001	SNP	12	0.0	scaffold1393.0	115
Pyd12_006	SNP	12	2.6	scaffold472.0	307,143
Pyd12_008	SNP	12	3.0	scaffold472.0	235,548
Pyd12_010	SNP	12	5.8	scaffold255.0	335,854
Pyb12_001	SNP	12	9.3	scaffold942.0	8,446
Pyb12_002	SNP	12	9.4	scaffold365.0	64,163
Pyb12_003	SNP	12	9.7	scaffold365.0	64,220
Pyb12_007	SNP	12	10.7	scaffold942.0	123,331
Pyb12_008	SNP	12	10.7	scaffold942.0	36,439
Pyb12_015	SNP	12	11.2	scaffold942.0	147,743
Pyb12_016	SNP	12	11.2	scaffold365.0	45,969
Pyb12_019	SNP	12	11.3	scaffold365.0	38,759
Pyb12_021	SNP	12	11.4	scaffold942.0	12,686
Pyd12_020	SNP	12	12.2	scaffold365.0	368,128
Pyb12_023	SNP	12	12.4	scaffold365.0	193,414
Pyb12_024	SNP	12	12.8	scaffold120.0	339,848
Pyb12_027	SNP	12	13.1	scaffold365.0	166,876
Pyb12_028	SNP	12	13.6	scaffold120.0	234,706
Pyb12_029	SNP	12	13.6	scaffold120.0	327,752
Pyb12_030	SNP	12	13.6	scaffold120.0	339,788
Pyb12_031	SNP	12	13.6	scaffold120.0	213,412
Pyb12_036	SNP	12	14.4	scaffold365.0	268,492
Pyb12_041	SNP	12	16.0	scaffold120.0	531,557
Pyb12_042	SNP	12	16.0	scaffold120.0	832,716
Pyb12_046	SNP	12	16.6	scaffold255.0	194,277
Pybd12_003	SNP	12	23.6	scaffold242.0	377,605
Pyb12_050	SNP	12	26.9	scaffold242.0	298,542
Pyb12_052	SNP	12	27.7	scaffold242.0	336,894

Pyb12_065	SNP	12	30.1	scaffold242.0	140,580
Pyb12_068	SNP	12	30.2	scaffold242.0	219,240
Pyb12_073	SNP	12	31.5	scaffold242.0	285,256
Pyb12_088	SNP	12	34.1	scaffold217.0	283,270
Pyb12_089	SNP	12	34.1	scaffold217.0	377,788
Pyb12_090	SNP	12	34.3	scaffold130.0	40,670
Pyb12_097	SNP	12	35.2	scaffold217.0	452,970
Pyb12_099	SNP	12	35.9	scaffold217.0	27,255
Pyb12_102	SNP	12	36.0	scaffold217.0	251,067
Pyb12_103	SNP	12	36.7	scaffold217.0	517,832
Pyb12_104	SNP	12	37.0	scaffold468.0	30,593
Pyb12_106	SNP	12	37.5	scaffold468.0	90,424
Pyb12_108	SNP	12	37.9	scaffold468.0	344,819
Pyb12_110	SNP	12	38.1	scaffold468.0	283,765
Pyb12_111	SNP	12	39.8	scaffold468.0	339,646
Pyb12_113	SNP	12	40.1	scaffold468.0	280,987
Pyb12_116	SNP	12	42.0	scaffold500.0	43,382
Pyb12_118	SNP	12	42.3	scaffold783.0	19,772
Pyb12_119	SNP	12	42.3	scaffold783.0	12,104
Pyb12_120	SNP	12	42.4	scaffold783.0	113,112
Pyb12_122	SNP	12	46.4	scaffold70.0	39,608
Pyb12_126	SNP	12	46.6	scaffold70.0	17,156
CH01f02	SSR	12	46.6	scaffold70.0	484,819-484,981
Pyb12_130	SNP	12	47.3	scaffold262.0	478,057
Pyb12_137	SNP	12	48.1	scaffold262.0	532,164
NB104a	SSR	12	49.1	scaffold303.0	394,431-394,599
CH01d09	SSR	12	52.0	scaffold15.0	1,174,649-1,174,791
Pyb12_142	SNP	12	52.5	scaffold15.0	555,135
Pyb12_143	SNP	12	52.5	scaffold15.0	597,735
Pyb12_146	SNP	12	53.2	scaffold15.0	593,801
Pyd12_031	SNP	12	69.7	scaffold1316.0	59,974
Pyd12_036	SNP	12	77.9	scaffold827.0	69,026
Pyb12_149	SNP	12	81.7	scaffold309.0	310,114
Pyb12_150	SNP	12	81.7	scaffold15.0	1,495,333
Pyb12_153	SNP	12	81.8	scaffold589.0	31,266
Pyb12_154	SNP	12	81.8	scaffold309.0	359,779
Pyb12_157	SNP	12	82.1	scaffold580.0	197,658
Pybd12_006	SNP	12	82.9	scaffold309.0	423,312
Pyb12_161	SNP	12	83.1	scaffold589.0	85,573
Pyb12_162	SNP	12	83.1	scaffold15.0	738,704
Pyb12_163	SNP	12	83.1	scaffold15.0	410,249
Pyb12_165	SNP	12	83.3	scaffold589.0	123,041
Pyb12_166	SNP	12	83.4	scaffold15.0	894,268
Pyb12_167	SNP	12	83.4	scaffold589.0	108,000

Pyb12_169	SNP	12	83.4	scaffold15.0	1,225,598
Pyb12_171	SNP	12	83.5	scaffold303.0	370,373
Pyb12_172	SNP	12	83.5	scaffold15.0	1,155,594
Pyb12_176	SNP	12	83.6	scaffold309.0	96,149
Pyb12_177	SNP	12	83.7	scaffold15.0	765,398
Pyb12_178	SNP	12	83.7	scaffold303.0	365,432
Pyb12_179	SNP	12	84.0	scaffold1037.0	37,136
Pyb12_180	SNP	12	84.8	scaffold15.0	741,890
Pyb12_181	SNP	12	85.8	scaffold402.0	293,037
Pyb12_183	SNP	12	86.2	scaffold402.0	211,725
Pyb12_186	SNP	12	86.5	scaffold402.0	308,720
Pyb12_187	SNP	12	86.5	scaffold402.0	282,380
Pybd12_007	SNP	12	87.4	scaffold827.0	126,368
Pyb12_189	SNP	12	87.4	scaffold319.0	313,034
Pyb12_190	SNP	12	88.4	scaffold319.0	71,742
Pyb12_192	SNP	12	88.9	scaffold478.0	162,864
Pyb12_195	SNP	12	89.1	scaffold478.0	144,911
Pyb12_196	SNP	12	89.1	scaffold319.0	71,784
Pyb12_198	SNP	12	90.1	scaffold204.0	420,107
Pyb12_201	SNP	12	92.0	scaffold395.1	9,127
Pyb12_202	SNP	12	92.3	scaffold889.0	149,182
Pyb12_203	SNP	12	92.3	scaffold395.1	57,387
Pybd12_008	SNP	12	93.7	scaffold379.0	103,319
Pyb12_212	SNP	12	94.5	scaffold445.0	309,265
Pyd12_039	SNP	12	95.2	scaffold89.0	616,278
Pyb12_220	SNP	12	95.5	scaffold953.0	39,162
Pyb12_221	SNP	12	95.5	scaffold1418.0	3,303
Pyb12_223	SNP	12	95.5	scaffold445.0	307,620
Pyb12_228	SNP	12	95.6	scaffold916.0	23,454
Pyd12_043	SNP	12	96.3	scaffold103.0	240,011
Pyb12_234	SNP	12	97.2	scaffold379.0	89,055
Pyb12_235	SNP	12	97.2	scaffold107.0	52,351
Pyb12_238	SNP	12	97.5	scaffold521.0	15,237
Pyb12_242	SNP	12	98.6	scaffold806.0	98,628
Pyb12_243	SNP	12	98.7	scaffold806.0	119,775
Pyb12_245	SNP	12	98.7	scaffold806.0	110,610
Pyd12_049	SNP	12	99.2	scaffold367.0	152,528
Pyb12_248	SNP	12	100.1	scaffold806.0	119,737
Pyb12_251	SNP	12	100.9	scaffold7.0	1,500,182
Pyb12_254	SNP	12	101.0	scaffold7.0	1,738,251
Pyb12_255	SNP	12	101.0	scaffold7.0	1,544,290
Pyb12_259	SNP	12	101.5	scaffold1052.0	18,325
Pyb12_261	SNP	12	102.1	scaffold7.0	1,261,545
Pyb12_262	SNP	12	102.1	scaffold7.0	1,312,747

Pyb12_263	SNP	12	102.1	scaffold7.0	1,417,387
CH04g04	SSR	12	102.3	scaffold7.0	1,304,491-1,304,656
Pyb12_270	SNP	12	103.2	scaffold7.0	1,432,227
Pyb12_271	SNP	12	103.3	scaffold7.0	1,423,574
Pyd12_062	SNP	12	114.2	scaffold7.0	1,168,751
Pyb12_279	SNP	12	117.9	scaffold266.0	509,715
Pyb12_280	SNP	12	117.9	scaffold103.0	8,959
Pyb12_281	SNP	12	117.9	scaffold103.0	120,169
Pyb12_283	SNP	12	118.0	scaffold103.0	77,986
Pyb12_286	SNP	12	119.0	scaffold103.0	146,951
Pyb12_288	SNP	12	120.2	scaffold103.0	287,199
Pyb12_291	SNP	12	122.1	scaffold103.0	234,316
Pyb12_292	SNP	12	122.1	scaffold103.0	234,362
Pyb12_296	SNP	12	122.3	scaffold228.0	187,663
Pyb12_297	SNP	12	122.3	scaffold228.0	473,206
Pyb12_298	SNP	12	122.3	scaffold228.0	385,813
Pyb12_300	SNP	12	122.3	scaffold249.0	34,178
Pyb12_301	SNP	12	122.3	scaffold228.0	592,736
Pyb12_303	SNP	12	122.3	scaffold228.0	255,341
Pyb12_304	SNP	12	122.3	scaffold228.0	77,129
Pyb12_306	SNP	12	122.3	scaffold249.0	57,741
Pyb12_307	SNP	12	122.3	scaffold228.0	578,970
Pyb12_308	SNP	12	122.3	scaffold228.0	300,892
Pyb12_312	SNP	12	122.4	scaffold228.0	237,918
Pyb12_313	SNP	12	122.4	scaffold228.0	343,459
Pyb12_314	SNP	12	122.4	scaffold228.0	424,109
Pyb12_316	SNP	12	122.7	scaffold228.0	429,799
CH05d04	SSR	12	122.9	scaffold128.0	177,666-177,843
Pyb12_319	SNP	12	123.3	scaffold264.0	429,874
Pyb12_320	SNP	12	125.5	scaffold128.0	246,691
Pyb12_322	SNP	12	125.5	scaffold128.0	626,948
Pyb12_324	SNP	12	127.3	scaffold143.0	694,330
Pyb12_327	SNP	12	127.7	scaffold143.0	54,316
Pyb12_328	SNP	12	127.7	scaffold1068.0	96,500
Pyb12_329	SNP	12	127.7	scaffold1068.0	73,579
Pyb12_332	SNP	12	127.7	scaffold128.0	66,324
Pyb12_333	SNP	12	127.7	scaffold143.0	477,588
Pyb12_334	SNP	12	127.7	scaffold143.0	8,700
Pyb12_335	SNP	12	127.8	scaffold143.0	430,658
Pyb12_336	SNP	12	127.8	scaffold143.0	690,406
Pyb12_337	SNP	12	127.8	scaffold143.0	314,290
Pyb12_338	SNP	12	127.8	scaffold1068.0	102,404
Pyb12_340	SNP	12	127.8	scaffold143.0	603,558
Pyb12_343	SNP	12	127.8	scaffold143.0	268,969

Pyb12_346	SNP	12	128.6	scaffold143.0	382,662
Pyb12_348	SNP	12	128.6	scaffold128.0	73,294
CH02e02	SSR	13	0.0	scaffold83.0	46,964-47,077
AU223486	SSR	13	3.7	scaffold51.0	882,852-883,061
Pyb13_004	SNP	13	5.2	scaffold174.0	36,243
Pyb13_013	SNP	13	6.2	scaffold19.0	503,063
Pyb13_014	SNP	13	6.6	scaffold535.0	90,022
Pyb13_015	SNP	13	7.4	scaffold48.0	408,192
Pyb13_016	SNP	13	7.5	scaffold48.0	556,005
Pyb13_017	SNP	13	7.5	scaffold48.0	597,558
Pyb13_018	SNP	13	7.5	scaffold48.0	429,617
Pyb13_020	SNP	13	7.5	scaffold48.0	408,135
Pyb13_021	SNP	13	7.5	scaffold48.0	670,478
Pyb13_022	SNP	13	7.5	scaffold48.0	616,913
Pyb13_023	SNP	13	8.3	scaffold48.0	991,369
Pyb13_025	SNP	13	8.5	scaffold48.0	1,114,353
Pyb13_026	SNP	13	8.5	scaffold48.0	998,771
Pyb13_027	SNP	13	8.5	scaffold48.0	1,060,170
Pyb13_030	SNP	13	9.1	scaffold451.0	72,791
Pyb13_032	SNP	13	10.5	scaffold83.0	28,453
Pyb13_034	SNP	13	10.8	scaffold51.0	176,215
Pyb13_039	SNP	13	11.1	scaffold83.0	793,295
Pyb13_043	SNP	13	11.4	scaffold83.0	517,238
Pyb13_044	SNP	13	11.4	scaffold66.0	788,076
Pyb13_048	SNP	13	11.7	scaffold83.0	290,830
Pyb13_050	SNP	13	11.8	scaffold83.0	371,861
Pyb13_051	SNP	13	11.8	scaffold83.0	508,053
Pyb13_052	SNP	13	11.8	scaffold83.0	569,977
Pyb13_056	SNP	13	11.9	scaffold83.0	863,257
Pyb13_058	SNP	13	12.0	scaffold83.0	540,151
Pyb13_059	SNP	13	12.0	scaffold83.0	546,781
Pyb13_063	SNP	13	12.1	scaffold83.0	490,425
Pyb13_067	SNP	13	12.3	scaffold83.0	67,232
Pyb13_069	SNP	13	12.3	scaffold83.0	13,039
Pyb13_071	SNP	13	12.3	scaffold51.0	190,104
Pyb13_073	SNP	13	12.3	scaffold83.0	290,801
Pyb13_074	SNP	13	12.3	scaffold83.0	67,423
Pyb13_075	SNP	13	12.5	scaffold451.0	3,473
Pyb13_076	SNP	13	12.5	scaffold83.0	28,507
Pyb13_077	SNP	13	12.6	scaffold51.0	84,093
Pyb13_078	SNP	13	12.6	scaffold51.0	1,530
Pyb13_079	SNP	13	12.7	scaffold51.0	692,690
Pyb13_084	SNP	13	12.8	scaffold51.0	552,117
Pyb13_085	SNP	13	12.8	scaffold51.0	667,857

Pyb13_086	SNP	13	12.8	scaffold51.0	588,472
Pyb13_087	SNP	13	12.9	scaffold51.0	776,805
Pyb13_088	SNP	13	12.9	scaffold51.0	534,459
Pyb13_092	SNP	13	13.1	scaffold51.0	908,323
Pyb13_094	SNP	13	13.3	scaffold451.0	249,688
Pyb13_095	SNP	13	13.3	scaffold51.0	705,953
Pyb13_096	SNP	13	13.3	scaffold451.0	281,046
Pyb13_098	SNP	13	13.4	scaffold51.0	328,997
Pyb13_100	SNP	13	13.4	scaffold51.0	342,565
Pyb13_101	SNP	13	13.4	scaffold51.0	841,772
Pyb13_102	SNP	13	13.7	scaffold51.0	817,661
Pyb13_104	SNP	13	13.8	scaffold51.0	722,478
Pyb13_105	SNP	13	13.8	scaffold51.0	491,416
Pyb13_107	SNP	13	14.0	scaffold51.0	1,162,888
Pyb13_109	SNP	13	14.5	scaffold51.0	338,342
Pyb13_111	SNP	13	15.7	scaffold83.0	710,193
Pyb13_112	SNP	13	15.8	scaffold83.0	706,668
Pyb13_117	SNP	13	19.2	scaffold51.0	238,227
Pyb13_118	SNP	13	19.6	scaffold66.0	839,668
Pyd13_005	SNP	13	36.1	scaffold451.0	286,039
Pyd13_006	SNP	13	38.3	scaffold51.0	884,159
Pyd13_007	SNP	13	38.4	scaffold51.0	898,009
Pyd13_010	SNP	13	38.4	scaffold22.0	555,429
Pyd13_011	SNP	13	38.4	scaffold219.0	48,073
Pyd13_012	SNP	13	39.6	scaffold735.0	854
Pyd13_013	SNP	13	39.6	scaffold411.0	113,754
Pyd13_014	SNP	13	40.3	scaffold22.0	907,551
Pyd13_016	SNP	13	42.9	scaffold22.0	1,087,018
Pyb13_130	SNP	13	49.4	scaffold83.0	874,148
Pyb13_132	SNP	13	49.8	scaffold83.0	764,586
Pyb13_133	SNP	13	50.3	scaffold66.0	809,196
Pyb13_134	SNP	13	50.5	scaffold66.0	723,874
Pyb13_135	SNP	13	50.6	scaffold66.0	800,977
Pyb13_138	SNP	13	50.9	scaffold83.0	878,063
Pyb13_143	SNP	13	51.8	scaffold693.0	195,392
Pybd13_003	SNP	13	53.5	scaffold83.0	482,901
CTG1060382	SSR	13	54.9	Not_anchored	-
Pyb13_163	SNP	13	55.3	scaffold693.0	191,132
Pyb13_165	SNP	13	56.2	scaffold693.0	180,048
Pyb13_168	SNP	13	56.5	scaffold439.0	104,638
Pyb13_169	SNP	13	56.7	scaffold693.0	122,204
Pyb13_170	SNP	13	56.9	scaffold439.0	119,503
Pyd13_021	SNP	13	59.0	scaffold693.0	138,176
Pyb13_172	SNP	13	59.2	scaffold274.0	342,179

Pybd13_006	SNP	13	60.0	scaffold28.0	1,183,049
Pyb13_175	SNP	13	61.0	scaffold28.0	879,077
Pyb13_176	SNP	13	61.2	scaffold28.0	898,364
Pyb13_177	SNP	13	61.2	scaffold28.0	882,923
Pyb13_178	SNP	13	61.3	scaffold28.0	883,841
Pyb13_180	SNP	13	61.5	scaffold28.0	786,644
Pyb13_181	SNP	13	61.9	scaffold28.0	515,819
Pyb13_183	SNP	13	62.2	scaffold28.0	1,107,516
CTG1067935	SSR	13	62.6	scaffold307.0	452,767-453,055
Pyb13_186	SNP	13	63.6	scaffold28.0	969,518
Pyb13_187	SNP	13	63.6	scaffold28.0	667,656
Pyb13_188	SNP	13	63.7	scaffold28.0	800,499
Pyb13_190	SNP	13	63.8	scaffold28.0	651,549
Pyb13_191	SNP	13	63.8	scaffold28.0	661,625
Pyb13_192	SNP	13	63.9	scaffold28.0	622,347
Pyb13_193	SNP	13	64.0	scaffold221.0	14,985
Pyb13_196	SNP	13	65.4	scaffold221.0	64,412
Pyd13_025	SNP	13	65.5	scaffold83.0	92,217
Pyd13_026	SNP	13	65.6	scaffold83.0	458,735
Pyb13_198	SNP	13	65.7	scaffold221.0	226,314
Pyb13_199	SNP	13	65.8	scaffold221.0	108,075
Pyb13_200	SNP	13	66.2	scaffold188.0	662,015
Pyd13_028	SNP	13	66.9	scaffold51.0	144,775
Pyb13_202	SNP	13	67.1	scaffold979.0	10,387
Pyb13_205	SNP	13	67.5	scaffold188.0	28,583
Pyb13_207	SNP	13	67.9	scaffold188.0	25,254
Pyb13_208	SNP	13	68.2	scaffold221.0	352,622
Pyb13_210	SNP	13	68.3	scaffold188.0	164,063
Pyb13_211	SNP	13	68.3	scaffold221.0	238,495
Pybd13_007	SNP	13	68.5	scaffold274.0	408,706
Pyd13_029	SNP	13	70.7	scaffold22.0	1,405,119
Pyd13_033	SNP	13	77.0	scaffold22.0	1,251,146
Pyd13_035	SNP	13	79.9	scaffold116.0	278,064
Pybd13_008	SNP	13	80.5	scaffold116.0	323,051
Pybd13_009	SNP	13	81.9	scaffold22.0	539,762
Pybd13_010	SNP	13	83.8	scaffold22.0	298,455
Pyd13_037	SNP	13	85.5	scaffold648.0	101,302
NB133a	SSR	13	86.0	scaffold411.0	57,798-58,078
Pybd13_011	SNP	13	86.5	scaffold22.0	969,715
Pyd13_041	SNP	13	86.6	scaffold129.0	13,321
Pybd13_013	SNP	13	88.1	scaffold116.0	83,743
Pyb13_219	SNP	13	90.8	scaffold116.0	186,600
Pybd13_014	SNP	13	90.8	scaffold116.0	19,071
Pybd13_015	SNP	13	92.3	scaffold116.0	124,640

Pyb13_224	SNP	13	92.5	scaffold22.0	236,801
Pyb13_226	SNP	13	93.0	scaffold22.0	749,617
Pyb13_229	SNP	13	93.1	scaffold22.0	463,986
Pyb13_232	SNP	13	93.3	scaffold22.0	1,059,493
Pyb13_237	SNP	13	93.7	scaffold22.0	398,014
Pyb13_242	SNP	13	94.6	scaffold22.0	1,172,014
Pyb13_243	SNP	13	94.9	scaffold22.0	1,207,402
Pyb13_244	SNP	13	95.0	scaffold22.0	1,147,145
Pybd13_017	SNP	13	97.5	scaffold22.0	1,050,986
Pyd13_050	SNP	13	98.9	scaffold219.0	203,390
Pyb13_250	SNP	13	99.3	scaffold221.0	155,743
Pyb13_251	SNP	13	100.1	scaffold221.0	226,359
Pyb13_252	SNP	13	100.1	scaffold221.0	89,814
Pyb13_253	SNP	13	102.5	scaffold28.0	983,678
Pyd13_054	SNP	13	104.1	scaffold819.0	30,365
Pyb13_255	SNP	13	105.5	scaffold28.0	1,314,579
Pyb13_256	SNP	13	105.5	scaffold28.0	1,147,394
Pyb13_257	SNP	13	105.7	scaffold274.0	260,626
Pyb13_258	SNP	13	106.2	scaffold28.0	1,331,502
Pyb13_260	SNP	13	106.3	scaffold274.0	293,961
Pyb13_262	SNP	13	106.7	scaffold28.0	1,337,567
Pyb13_273	SNP	13	116.8	scaffold66.0	714,490
Pyb13_276	SNP	13	121.7	scaffold48.0	644,206
Pyb13_277	SNP	13	121.7	scaffold48.0	560,792
Pyb14_004	SNP	14	0.0	scaffold142.0	415,114
NH004a	SSR	14	0.2	scaffold326.0	128,156-128,258
Pybd14_001	SNP	14	6.8	scaffold142.0	761,632
Pybd14_002	SNP	14	8.1	scaffold17.0	302,538
Pybd14_003	SNP	14	8.6	scaffold25.0	759,357
Pyb14_006	SNP	14	9.0	scaffold17.0	128,695
Pyb14_007	SNP	14	10.0	scaffold641.0	140,740
Pyb14_010	SNP	14	10.3	scaffold421.0	377,455
Pyb14_011	SNP	14	10.4	scaffold17.0	316,992
Pyb14_012	SNP	14	10.5	scaffold17.0	391,785
Pyb14_013	SNP	14	10.5	scaffold17.0	476,600
Pyb14_014	SNP	14	10.5	scaffold17.0	453,135
Pyb14_015	SNP	14	10.5	scaffold17.0	263,268
Pyb14_016	SNP	14	10.5	scaffold17.0	246,866
Pyb14_023	SNP	14	11.1	scaffold17.0	213,135
Pyb14_025	SNP	14	11.5	scaffold421.0	192,153
Pybd14_004	SNP	14	12.1	scaffold17.0	369,839
Pybd14_005	SNP	14	12.4	scaffold17.0	418,250
Pybd14_006	SNP	14	12.4	scaffold17.0	418,299
Pyb14_027	SNP	14	12.8	scaffold25.0	275,351

Pyb14_028	SNP	14	12.9	scaffold25.0	1,115,747
Pyb14_029	SNP	14	13.1	scaffold25.0	778,409
Pyb14_039	SNP	14	13.6	scaffold142.0	82,134
Pyb14_042	SNP	14	13.7	scaffold25.0	166,679
Pyb14_045	SNP	14	14.1	scaffold291.0	443,548
Pyb14_046	SNP	14	14.4	scaffold25.0	144,307
Pyb14_047	SNP	14	14.4	scaffold142.0	578,887
Pyb14_050	SNP	14	14.5	scaffold25.0	584,643
Pyb14_051	SNP	14	15.3	scaffold25.0	1,151,847
Pyb14_056	SNP	14	18.6	scaffold1141.0	75,186
CH05g07	SSR	14	18.8	scaffold548.0	27-218
Pyb14_058	SNP	14	19.3	scaffold548.0	77,872
Pyb14_059	SNP	14	19.3	scaffold53.0	226,442
Pyb14_061	SNP	14	19.4	scaffold991.0	99,902
Pyb14_062	SNP	14	19.4	scaffold548.0	80,428
Pyb14_063	SNP	14	19.4	scaffold991.0	99,712
EMPc108	SSR	14	19.5	scaffold991.0	33,573-33,719
Pybd14_008	SNP	14	20.8	scaffold17.0	154,569
Pybd14_010	SNP	14	23.3	scaffold42.0	427,952
Pybd14_011	SNP	14	31.2	scaffold374.0	125,405
Pyb14_069	SNP	14	34.9	scaffold421.0	337,059
Pyb14_071	SNP	14	35.4	scaffold1444.0	9,808
Pyb14_075	SNP	14	36.4	scaffold1444.0	5,149
Pybd14_014	SNP	14	38.6	scaffold432.0	200,461
Pyb14_078	SNP	14	40.2	scaffold42.0	513,689
Pyb14_079	SNP	14	40.3	scaffold42.0	384,625
Pyd14_019	SNP	14	40.7	scaffold107.0	675,067
Pybd14_015	SNP	14	40.9	scaffold107.0	670,199
Pyb14_083	SNP	14	41.3	scaffold1526.0	3,989
Pyb14_092	SNP	14	42.0	scaffold1056.0	90,759
Pyb14_093	SNP	14	42.0	scaffold799.0	146,876
Pyb14_097	SNP	14	42.0	scaffold799.0	108,755
Pyb14_102	SNP	14	42.9	scaffold799.0	101,343
Pyb14_103	SNP	14	42.9	scaffold1501.0	5,696
Pyb14_105	SNP	14	42.9	scaffold799.0	146,493
Pyb14_111	SNP	14	43.7	scaffold107.0	328,287
Pyb14_114	SNP	14	44.1	scaffold107.0	340,622
Pyb14_115	SNP	14	45.2	scaffold53.0	232,610
Pybd14_018	SNP	14	61.5	scaffold168.0	87,913
Pyb14_124	SNP	14	66.6	scaffold75.0	478,085
Pyb14_129	SNP	14	67.1	scaffold736.0	89,940
Pyb14_134	SNP	14	67.5	scaffold431.0	383,945
Pybd14_021	SNP	14	68.9	scaffold736.0	60,332
Pyb14_141	SNP	14	69.3	scaffold934.0	48,774

CH04f06	SSR	14	69.5	scaffold987.0	97,377-97,534
Pyb14_145	SNP	14	69.6	scaffold987.0	113,621
Pyb14_153	SNP	14	70.0	scaffold811.0	44,759
Pyb14_163	SNP	14	70.8	scaffold272.0	54,686
Pyb14_165	SNP	14	71.0	scaffold859.0	164,737
Pyb14_168	SNP	14	71.1	scaffold859.0	98,157
Pyb14_169	SNP	14	71.3	scaffold934.0	70,003
Pyb14_172	SNP	14	71.8	scaffold75.0	704,330
Pyb14_176	SNP	14	74.0	scaffold744.0	41,903
Pyd14_031	SNP	14	74.1	scaffold626.0	238,858
NH001c	SSR	14	74.1	scaffold744.0	31,586-31,706
Pyb14_177	SNP	14	74.2	scaffold413.0	406,595
Pyb14_178	SNP	14	74.2	scaffold168.0	403,757
Pyd14_032	SNP	14	74.6	scaffold626.0	93,334
Pyb14_180	SNP	14	74.9	scaffold168.0	253,631
Pyb14_182	SNP	14	75.3	scaffold168.0	162,658
CH05g11	SSR	14	75.5	scaffold168.0	197,526-197,749
CH04c07	SSR	14	75.7	scaffold379.0	362,773-362,928
Pyd14_035	SNP	14	75.8	scaffold431.0	245,336
Pybd14_023	SNP	14	76.2	scaffold106.0	153,818
Pyd14_036	SNP	14	79.0	scaffold168.0	308,443
Pyb14_184	SNP	14	84.7	scaffold44.2	434,639
Pyb14_186	SNP	14	85.0	scaffold44.2	579,103
Pyb14_187	SNP	14	85.2	scaffold74.0	860,145
Pyb14_189	SNP	14	85.7	scaffold44.2	523,914
Pyb14_191	SNP	14	85.8	scaffold74.0	331,926
Pyb14_192	SNP	14	85.8	scaffold44.2	550,829
Pyb14_193	SNP	14	85.9	scaffold44.2	456,448
Pyb14_194	SNP	14	86.4	scaffold1265.0	44,376
Pyb14_195	SNP	14	86.6	scaffold74.0	545,858
Pyb14_198	SNP	14	86.8	scaffold74.0	868,201
Pyb14_199	SNP	14	86.8	scaffold74.0	670,201
Pyb14_200	SNP	14	86.8	scaffold74.0	855,313
Pyb14_201	SNP	14	86.9	scaffold74.0	611,005
Pyb14_202	SNP	14	87.0	scaffold74.0	447,508
Pyb14_204	SNP	14	87.2	scaffold74.0	677,516
Pyb14_207	SNP	14	93.4	scaffold14.0	1,363,717
Pyb14_208	SNP	14	93.5	scaffold14.0	1,662,276
Pyb14_209	SNP	14	93.5	scaffold14.0	866,553
Pyb14_210	SNP	14	93.5	scaffold14.0	1,070,387
CH03a03	SSR	14	93.7	scaffold14.0	1,072,634-1,072,230
Pyb14_211	SNP	14	94.1	scaffold14.0	1,372,097
Pyb14_215	SNP	14	97.7	scaffold82.0	989,292
Pyb14_217	SNP	14	99.5	scaffold82.0	586,069

Pyb14_219	SNP	14	99.9	scaffold82.0	596,453
Pyb14_220	SNP	14	99.9	scaffold82.0	550,704
Pyb14_221	SNP	14	99.9	scaffold82.0	598,367
Pyb14_222	SNP	14	99.9	scaffold82.0	459,769
Pyb14_223	SNP	14	100.0	scaffold82.0	676,685
Pyb14_224	SNP	14	100.3	scaffold82.0	471,389
Pyb14_226	SNP	14	100.3	scaffold82.0	225,832
Pyb14_227	SNP	14	100.4	scaffold82.0	202,439
Pyb14_228	SNP	14	100.4	scaffold82.0	131,297
Pyb14_231	SNP	14	100.6	scaffold82.0	162,203
Pyb14_235	SNP	14	100.7	scaffold82.0	95,721
Pyb14_238	SNP	14	101.1	scaffold82.0	345,431
Pyb14_242	SNP	14	101.5	scaffold82.0	596,514
Pyb14_246	SNP	14	102.0	scaffold82.0	369,453
Pybd14_025	SNP	14	102.7	scaffold616.0	260,680
Pyd14_045	SNP	14	108.0	scaffold82.0	598,327
Pyd14_047	SNP	14	109.9	scaffold14.0	474,492
Pyd14_049	SNP	14	110.0	scaffold14.0	632,674
Pyd14_051	SNP	14	113.6	scaffold14.0	907,649
Pybd14_027	SNP	14	119.7	scaffold14.0	1,178,816
Pyb14_247	SNP	14	120.6	scaffold82.0	447,966
Pyb14_248	SNP	14	120.6	scaffold82.0	596,282
Pyb14_249	SNP	14	121.2	scaffold82.0	928,263
Pyb14_254	SNP	14	122.4	scaffold82.0	965,306
Pyd14_058	SNP	14	122.9	scaffold106.0	111,774
Pyb14_258	SNP	14	123.8	scaffold14.0	673,278
Pyb14_260	SNP	14	125.5	scaffold14.0	880,516
Pyb14_261	SNP	14	125.6	scaffold14.0	1,415,562
Pyb14_262	SNP	14	125.7	scaffold14.0	1,661,030
Pyb14_267	SNP	14	126.3	scaffold106.0	695,970
Pyd14_059	SNP	14	126.6	scaffold74.0	631,647
Pyd14_060	SNP	14	126.7	scaffold74.0	944,808
Pyb14_271	SNP	14	128.5	scaffold14.0	1,212,814
Pybd14_028	SNP	14	128.6	scaffold14.0	1,084,448
Pyb14_273	SNP	14	128.7	scaffold14.0	807,834
Pyb14_274	SNP	14	128.8	scaffold14.0	1,157,487
Pyb14_275	SNP	14	128.8	scaffold14.0	1,191,352
Pyb14_276	SNP	14	128.8	scaffold708.0	2,058
Pyb14_282	SNP	14	129.1	scaffold708.0	13,217
Pyb14_284	SNP	14	129.4	scaffold14.0	921,638
Pyb14_294	SNP	14	130.6	scaffold106.0	889,747
Pyb14_297	SNP	14	131.0	scaffold106.0	562,948
Pyb14_298	SNP	14	131.0	scaffold106.0	691,339
Pyb14_300	SNP	14	131.4	scaffold106.0	558,077

Pyb14_301	SNP	14	131.6	scaffold106.0	682,793
Pyb14_302	SNP	14	131.8	scaffold106.0	865,027
Pyb14_305	SNP	14	133.6	scaffold106.0	501,210
Pyb14_307	SNP	14	133.6	scaffold106.0	132,413
Pyb14_310	SNP	14	133.7	scaffold106.0	155,865
Pyb14_311	SNP	14	133.7	scaffold106.0	338,010
CH05d03	SSR	14	133.9	scaffold106.0	332,515-332,681
Pyb14_317	SNP	14	138.0	scaffold744.0	2,576
Pyb14_318	SNP	14	138.0	scaffold744.0	31,271
Pyb14_319	SNP	14	138.0	scaffold168.0	550,001
Pyd14_066	SNP	14	139.2	scaffold596.0	2,502
Pyd14_067	SNP	14	139.2	scaffold811.0	104,198
Pyb14_321	SNP	14	139.6	scaffold744.0	134,598
Pyb14_323	SNP	14	140.4	scaffold75.0	701,807
Pyb14_324	SNP	14	140.7	scaffold75.0	637,784
Pyd14_075	SNP	14	140.9	scaffold431.0	392,192
Pyb14_325	SNP	14	141.0	scaffold75.0	471,941
Pyb14_329	SNP	14	141.4	scaffold75.0	719,666
Pyb14_330	SNP	14	141.4	scaffold626.0	97,832
Pyb14_331	SNP	14	141.5	scaffold75.0	918,037
Pyb14_333	SNP	14	141.5	scaffold75.0	880,207
Pyd14_076	SNP	14	142.7	scaffold950.0	146,683
Pyb14_339	SNP	14	147.1	scaffold272.0	139,421
Pyb14_344	SNP	14	156.1	scaffold53.0	226,411
Pyb14_345	SNP	14	156.1	scaffold814.0	172,978
Pyb14_351	SNP	14	156.8	scaffold42.0	503,796
Pyb14_355	SNP	14	158.5	scaffold548.0	2,259
Pyb15_005	SNP	15	0.0	scaffold23.0	1,085,527
Pyb15_006	SNP	15	0.0	scaffold23.0	1,152,388
Pyb15_007	SNP	15	0.1	scaffold23.0	823,975
Pyb15_008	SNP	15	0.9	scaffold23.0	1,177,121
Pyb15_009	SNP	15	1.1	scaffold23.0	663,709
Pyd15_002	SNP	15	7.0	scaffold13.0	640,507
Pyd15_003	SNP	15	7.1	scaffold13.0	806,077
Pyd15_004	SNP	15	7.1	scaffold13.0	769,025
Pyd15_006	SNP	15	10.2	scaffold13.0	1,107,560
Pyb15_014	SNP	15	11.2	scaffold360.0	385,187
Pyb15_017	SNP	15	11.4	scaffold46.0	1,181,748
Pybd15_001	SNP	15	12.1	scaffold46.0	371,529
Pyb15_018	SNP	15	12.1	scaffold401.0	331,831
Pyb15_022	SNP	15	12.2	scaffold314.0	421,738
Pyb15_029	SNP	15	14.0	scaffold159.0	88,115
Pyb15_031	SNP	15	17.6	scaffold154.0	622,574
Pyb15_032	SNP	15	18.1	scaffold154.0	197,932

Pyb15_033	SNP	15	18.2	scaffold154.0	322,417
Pyd15_007	SNP	15	18.2	scaffold3.0	1,883,023
Pybd15_003	SNP	15	18.7	scaffold3.0	1,055,127
Pyb15_035	SNP	15	18.7	scaffold3.0	350,192
Pyb15_036	SNP	15	18.7	scaffold3.0	973,262
Pyb15_037	SNP	15	18.9	scaffold13.0	47,157
Pyb15_038	SNP	15	19.3	scaffold3.0	531,817
Pyb15_039	SNP	15	19.3	scaffold3.0	844,414
Pyb15_040	SNP	15	19.4	scaffold3.0	370,430
Pyb15_042	SNP	15	19.5	scaffold3.0	248,583
Pyb15_043	SNP	15	19.5	scaffold3.0	757,832
Pyb15_044	SNP	15	19.6	scaffold3.0	622,669
Pyb15_046	SNP	15	19.9	scaffold3.0	1,685,288
Pyb15_047	SNP	15	19.9	scaffold3.0	1,513,215
Pyb15_048	SNP	15	20.1	scaffold154.0	178,073
Pyb15_051	SNP	15	20.2	scaffold3.0	3,015,034
Pyb15_052	SNP	15	20.4	scaffold3.0	1,382,031
Pyb15_053	SNP	15	20.5	scaffold3.0	1,262,065
Pyb15_054	SNP	15	20.5	scaffold3.0	1,382,074
Pybd15_004	SNP	15	20.8	scaffold13.0	409,257
Pyb15_058	SNP	15	21.2	scaffold13.0	54,837
Pyb15_059	SNP	15	21.2	scaffold13.0	646,279
Pyb15_060	SNP	15	21.2	scaffold13.0	54,890
Pyb15_061	SNP	15	21.6	scaffold13.0	892,986
Pyb15_062	SNP	15	21.6	scaffold13.0	1,148,822
Pyb15_064	SNP	15	21.8	scaffold3.0	2,197,808
Pyb15_065	SNP	15	21.8	scaffold3.0	2,917,983
Pyb15_066	SNP	15	21.9	scaffold3.0	3,777,776
Pyb15_067	SNP	15	21.9	scaffold3.0	3,005,051
Pyb15_068	SNP	15	21.9	scaffold3.0	3,002,968
Pyb15_069	SNP	15	21.9	scaffold3.0	2,945,737
Pyb15_070	SNP	15	21.9	scaffold3.0	2,299,621
Pyb15_071	SNP	15	21.9	scaffold3.0	3,150,929
Pyb15_072	SNP	15	21.9	scaffold3.0	3,028,780
Pyb15_073	SNP	15	22.0	scaffold3.0	2,495,866
Pyb15_075	SNP	15	22.0	scaffold3.0	2,731,572
Pyb15_077	SNP	15	22.0	scaffold3.0	3,300,534
Pyb15_079	SNP	15	22.0	scaffold3.0	2,864,271
Pyb15_081	SNP	15	22.3	scaffold3.0	2,715,636
Pyd15_014	SNP	15	22.3	scaffold3.0	833,588
Pyb15_082	SNP	15	22.4	scaffold13.0	90,977
Pyb15_084	SNP	15	22.6	scaffold13.0	542,857
Pyb15_085	SNP	15	22.6	scaffold13.0	978,039
Pyb15_086	SNP	15	22.6	scaffold13.0	47,099

Pyb15_088	SNP	15	22.6	scaffold13.0	962,989
Pyb15_089	SNP	15	22.6	scaffold13.0	507,700
Pyb15_090	SNP	15	22.6	scaffold13.0	49,131
Pyb15_091	SNP	15	22.6	scaffold13.0	979,542
Pyb15_093	SNP	15	22.8	scaffold13.0	905,388
Pyb15_094	SNP	15	22.8	scaffold13.0	1,021,274
Pyb15_095	SNP	15	22.8	scaffold13.0	139,791
NH027a	SSR	15	22.9	scaffold3.0	3,734,538-3,734,677
Pyb15_099	SNP	15	23.2	scaffold3.0	2,646,902
Pyb15_100	SNP	15	23.3	scaffold3.0	2,188,079
Pyb15_104	SNP	15	23.5	scaffold3.0	2,884,231
Pyb15_105	SNP	15	23.5	scaffold3.0	2,989,218
Pyb15_109	SNP	15	23.8	scaffold3.0	2,945,771
Pyb15_112	SNP	15	24.2	scaffold13.0	317,936
Pyb15_113	SNP	15	24.4	scaffold13.0	1,038,983
Pyb15_114	SNP	15	24.5	scaffold13.0	901,238
Pyb15_115	SNP	15	24.8	scaffold3.0	2,519,666
CH02d10b	SSR	15	28.1	scaffold159.0	127,400-127,555
Pyd15_030	SNP	15	33.6	scaffold3.0	3,090,304
Pybd15_005	SNP	15	34.5	scaffold3.0	3,090,257
Pyb15_122	SNP	15	35.1	scaffold3.0	3,457,150
Pyb15_124	SNP	15	35.4	scaffold3.0	3,107,251
Pybd15_006	SNP	15	35.6	scaffold3.0	3,010,377
Pyb15_128	SNP	15	35.8	scaffold3.0	1,979,770
Pyb15_129	SNP	15	35.8	scaffold3.0	2,623,306
Pyb15_130	SNP	15	35.8	scaffold3.0	3,248,011
Pyb15_131	SNP	15	35.9	scaffold3.0	1,874,222
Pyb15_132	SNP	15	35.9	scaffold3.0	2,124,196
Pyb15_133	SNP	15	35.9	scaffold3.0	2,340,384
Pyd15_035	SNP	15	36.2	scaffold3.0	2,822,770
Pyb15_137	SNP	15	36.3	scaffold3.0	2,344,317
Pyb15_139	SNP	15	36.7	scaffold3.0	2,178,736
Pyb15_140	SNP	15	36.9	scaffold3.0	1,385,769
Pyb15_144	SNP	15	37.5	scaffold3.0	1,761,748
Pyb15_145	SNP	15	37.7	scaffold3.0	614,733
Pyb15_146	SNP	15	37.8	scaffold3.0	318,727
Pyb15_147	SNP	15	37.8	scaffold154.0	345,934
Pyb15_148	SNP	15	37.8	scaffold3.0	662,527
Pyb15_150	SNP	15	37.9	scaffold154.0	354,737
Pyb15_153	SNP	15	38.3	scaffold3.0	625,074
Pybd15_007	SNP	15	38.9	scaffold3.0	1,235,461
Pyb15_155	SNP	15	39.2	scaffold154.0	651,199
CH03b06	SSR	15	39.4	scaffold154.0	401,346-400,998
Pyb15_157	SNP	15	39.7	scaffold154.0	413,775

Pyb15_158	SNP	15	39.7	scaffold154.0	489,599
Pyb15_159	SNP	15	39.7	scaffold154.0	591,168
Pyb15_162	SNP	15	40.2	scaffold154.0	645,485
Pyd15_041	SNP	15	41.7	scaffold3.0	565,227
Pyb15_168	SNP	15	42.8	scaffold111.0	121,668
Pyb15_171	SNP	15	42.9	scaffold159.0	143,280
Pyb15_172	SNP	15	42.9	scaffold159.0	693,515
Pyb15_173	SNP	15	42.9	scaffold316.0	86,414
Pyb15_174	SNP	15	42.9	scaffold111.0	45,537
Pyb15_175	SNP	15	42.9	scaffold159.0	180,593
Pyb15_176	SNP	15	42.9	scaffold111.0	28,781
Pyb15_178	SNP	15	43.6	scaffold46.0	329,239
Pyb15_179	SNP	15	43.7	scaffold46.0	329,503
Pyb15_180	SNP	15	43.8	scaffold46.0	375,381
Pyb15_181	SNP	15	43.9	scaffold46.0	406,039
Pyb15_182	SNP	15	44.2	scaffold46.0	1,008,436
Pybd15_008	SNP	15	44.4	scaffold111.0	129,682
Pyb15_183	SNP	15	44.7	scaffold64.0	439,391
Pyb15_185	SNP	15	44.9	scaffold360.0	325,973
Pyb15_186	SNP	15	44.9	scaffold360.0	335,004
Pyb15_187	SNP	15	44.9	scaffold64.0	262,811
Pyb15_188	SNP	15	44.9	scaffold64.0	345,323
Pyb15_189	SNP	15	44.9	scaffold64.0	449,052
Pyb15_190	SNP	15	44.9	scaffold46.0	1,002,984
Pyb15_191	SNP	15	45.0	scaffold64.0	196,478
Pyb15_197	SNP	15	45.8	scaffold64.0	1,075,145
Pyb15_200	SNP	15	46.8	scaffold154.0	419,062
Pybd15_009	SNP	15	47.1	scaffold159.0	110,983
Pyb15_202	SNP	15	48.0	scaffold172.0	517,011
Pyb15_203	SNP	15	48.0	scaffold172.0	717,156
Pyb15_204	SNP	15	48.2	scaffold172.0	511,529
Pyb15_206	SNP	15	48.3	scaffold172.0	638,366
Pyb15_207	SNP	15	48.3	scaffold172.0	542,892
Pyb15_212	SNP	15	48.7	scaffold172.0	322,716
Pyd15_048	SNP	15	53.4	scaffold172.0	244,505
Pyd15_050	SNP	15	53.5	scaffold172.0	325,342
Pyd15_052	SNP	15	53.9	scaffold250.0	391,269
Pyd15_053	SNP	15	54.7	scaffold23.0	387,548
Pyb15_218	SNP	15	56.1	scaffold64.0	1,111,997
Pyb15_219	SNP	15	57.6	scaffold250.0	287,146
Pyb15_220	SNP	15	58.0	scaffold172.0	581,920
Pyd15_055	SNP	15	58.1	scaffold457.0	352,481
Pybd15_010	SNP	15	61.2	scaffold360.0	215,773
Pybd15_011	SNP	15	64.9	scaffold64.0	48,421

Pyb15_224	SNP	15	67.8	scaffold64.0	154,453
Pyb15_225	SNP	15	68.9	scaffold64.0	430,245
Pyb15_235	SNP	15	71.7	scaffold172.0	700,168
Pyb15_237	SNP	15	72.2	scaffold172.0	305,234
Pyb15_239	SNP	15	72.3	scaffold172.0	293,674
Pyb15_241	SNP	15	72.5	scaffold250.0	399,721
Pyb15_242	SNP	15	72.5	scaffold968.0	91,145
Pyb15_243	SNP	15	72.6	scaffold23.0	484,745
Pyb15_245	SNP	15	72.6	scaffold250.0	62,828
Pyb15_247	SNP	15	72.8	scaffold23.0	129,281
Pyb15_250	SNP	15	73.0	scaffold23.0	536,619
Pyb15_251	SNP	15	73.1	scaffold250.0	374,932
Pyb15_252	SNP	15	73.1	scaffold172.0	210,087
Pyb15_253	SNP	15	73.1	scaffold23.0	272,888
Pyb15_254	SNP	15	73.1	scaffold968.0	82,988
Pyb15_255	SNP	15	73.2	scaffold23.0	577,293
Pyb15_256	SNP	15	73.2	scaffold250.0	340,019
Pyb15_258	SNP	15	73.3	scaffold250.0	253,096
Pyb15_259	SNP	15	73.3	scaffold250.0	335,280
CTG1063001	SSR	15	73.4	scaffold23.0	371,078-371,275
Pyb15_264	SNP	15	74.3	scaffold23.0	641,840
Pyb15_265	SNP	15	74.3	scaffold23.0	827,926
Pybd15_013	SNP	15	74.4	scaffold23.0	455,161
Pyb15_269	SNP	15	74.4	scaffold23.0	1,058,823
Pyb15_273	SNP	15	74.5	scaffold23.0	1,129,437
IPPN08	SSR	15	74.5	scaffold23.0	1,195,905-1,196,056
Pyb15_275	SNP	15	74.5	scaffold23.0	750,525
CH01d08	SSR	15	74.6	scaffold23.0	536,654-536,945
Pyb15_276	SNP	15	74.6	scaffold23.0	1,368,614
Pyb15_278	SNP	15	74.8	scaffold23.0	914,465
Pyb15_279	SNP	15	74.9	scaffold23.0	1,227,598
Pyb15_281	SNP	15	74.9	scaffold23.0	969,138
Pyb15_282	SNP	15	74.9	scaffold23.0	1,227,629
Pyb15_284	SNP	15	75.6	scaffold1200.0	24,012
Pyb15_286	SNP	15	75.7	scaffold23.0	1,426,750
Pyb15_287	SNP	15	75.8	scaffold564.0	201,151
Pyb15_291	SNP	15	76.2	scaffold1200.0	51,211
Pyb15_293	SNP	15	76.3	scaffold1200.0	79,023
Pyb15_294	SNP	15	76.7	scaffold564.0	249,419
Pyb15_296	SNP	15	77.4	scaffold1200.0	51,273
Pyb15_297	SNP	15	77.8	scaffold564.0	45,550
Pyb15_298	SNP	15	78.0	scaffold564.0	56,437
Pyb15_299	SNP	15	78.0	scaffold76.0	659,044
Pyb15_303	SNP	15	78.4	scaffold76.0	837,871

Pyb15_304	SNP	15	78.5	scaffold76.0	483,316
Pyb15_305	SNP	15	78.5	scaffold564.0	129,284
Pyb15_306	SNP	15	78.5	scaffold564.0	149,961
Pyb15_307	SNP	15	78.5	scaffold76.0	493,835
Pyb15_309	SNP	15	78.5	scaffold76.0	1,030,249
Pyb15_310	SNP	15	78.5	scaffold76.0	791,067
Pyb15_311	SNP	15	78.7	scaffold76.0	450,143
Pyb15_312	SNP	15	78.9	scaffold76.0	451,510
Pyb15_314	SNP	15	79.2	scaffold66.0	315,318
Pyb15_315	SNP	15	79.3	scaffold76.0	379,568
Pyb15_316	SNP	15	79.3	scaffold76.0	373,843
Pyb15_317	SNP	15	79.5	scaffold76.0	817,046
Pyb15_318	SNP	15	79.7	scaffold76.0	551,810
NB129a	SSR	15	80.0	scaffold76.0	521,018-520,472
Pyb15_322	SNP	15	80.2	scaffold76.0	240,941
Pyb15_325	SNP	15	80.6	scaffold76.0	72,810
Pyb15_328	SNP	15	80.6	scaffold457.0	226,962
Pyb15_330	SNP	15	81.1	scaffold211.0	267,428
Pyb15_332	SNP	15	81.6	scaffold211.0	486,178
Pyb15_334	SNP	15	82.0	scaffold211.0	322,927
Pyb15_336	SNP	15	82.0	scaffold211.0	291,549
Pyb15_339	SNP	15	82.0	scaffold211.0	219,251
Pybd15_016	SNP	15	88.8	scaffold66.0	480,150
Pybd15_017	SNP	15	90.3	scaffold76.0	881,124
Pybd15_018	SNP	15	91.0	scaffold76.0	779,700
Pyb15_343	SNP	15	93.0	scaffold66.0	299,726
Pyd15_071	SNP	15	94.5	scaffold569.0	80,626
Pyb15_349	SNP	15	97.9	scaffold76.0	533,632
Pyb15_350	SNP	15	98.2	scaffold66.0	214,671
Pyb15_351	SNP	15	98.3	scaffold457.0	217,385
Pyb15_352	SNP	15	98.7	scaffold211.0	566,189
Pyb15_353	SNP	15	99.1	scaffold457.0	299,273
Pyb15_354	SNP	15	99.3	scaffold332.0	33,244
Pyb15_355	SNP	15	99.3	scaffold332.0	413,464
Pyb15_357	SNP	15	99.6	scaffold211.0	309,323
Pybd15_022	SNP	15	99.8	scaffold258.0	535,862
Pyb15_358	SNP	15	100.0	scaffold76.0	43,332
Pyb15_359	SNP	15	100.0	scaffold66.0	204,547
Pyb15_361	SNP	15	100.0	scaffold457.0	317,270
Pybd15_023	SNP	15	100.5	scaffold211.0	121,637
Pyb15_365	SNP	15	100.7	scaffold211.0	110,406
Pyb15_367	SNP	15	101.0	scaffold211.0	311,416
Pyb15_368	SNP	15	101.1	scaffold332.0	123,618
Pyb15_372	SNP	15	101.4	scaffold145.0	199,919

Pyd15_074	SNP	15	101.7	scaffold122.0	641,950
Pyb15_374	SNP	15	101.9	scaffold258.0	502,099
Pyb15_377	SNP	15	102.1	scaffold520.0	106,902
Pyb15_382	SNP	15	102.2	scaffold211.0	136,383
Pyb15_384	SNP	15	102.3	scaffold294.0	402,744
Pyb15_385	SNP	15	102.3	scaffold294.0	516,955
Pyb15_386	SNP	15	102.4	scaffold145.0	158,354
EMPc104	SSR	15	102.5	scaffold294.0	420,853-420,951
Pyd15_078	SNP	15	102.8	scaffold361.0	323,354
Pyb15_391	SNP	15	103.3	scaffold877.0	4,833
Pyb15_392	SNP	15	103.3	scaffold686.0	221,459
Pyb15_393	SNP	15	103.3	scaffold520.0	226,562
Pyb15_394	SNP	15	103.4	scaffold226.0	26,754
Pyb15_395	SNP	15	103.4	scaffold877.0	80,447
Pyb15_398	SNP	15	103.6	scaffold226.0	445,261
Pyb15_399	SNP	15	103.6	scaffold226.0	57,928
Pyb15_400	SNP	15	103.7	scaffold226.0	456,342
Pyb15_401	SNP	15	103.7	scaffold22.0	1,086,984
Pyb15_403	SNP	15	103.7	scaffold226.0	351,966
Pyb15_404	SNP	15	104.0	scaffold122.0	606,396
Pybd15_026	SNP	15	104.0	scaffold520.0	325,569
Pyb15_406	SNP	15	104.4	scaffold122.0	766,878
Pyb15_408	SNP	15	104.4	scaffold122.0	778,998
Pyb15_411	SNP	15	104.5	scaffold38.0	219,702
Pyb15_413	SNP	15	104.5	scaffold38.0	218,323
Pyb15_417	SNP	15	104.8	scaffold26.0	1,109,208
Pyb15_420	SNP	15	104.9	scaffold122.0	693,460
Pyb15_423	SNP	15	104.9	scaffold122.0	663,935
Pyd15_088	SNP	15	104.9	scaffold858.0	53,096
Pyb15_424	SNP	15	104.9	scaffold361.0	350,511
Pyb15_425	SNP	15	105.0	scaffold361.0	160,692
Pyb15_430	SNP	15	105.5	scaffold298.0	462,187
Pybd15_028	SNP	15	105.8	scaffold686.0	6,696
Pybd15_030	SNP	15	106.8	scaffold122.0	281,488
Pyb15_438	SNP	15	108.1	scaffold1131.0	47,843
Pybd15_031	SNP	15	109.4	scaffold361.0	49,208
Pyb15_444	SNP	15	110.7	scaffold186.0	40,856
Pyb15_445	SNP	15	110.9	scaffold437.0	259,392
Pyb15_446	SNP	15	110.9	scaffold437.0	370,215
Pyb15_447	SNP	15	111.1	scaffold186.0	5,501
Pyb15_448	SNP	15	111.2	scaffold1512.0	503
Pyb15_449	SNP	15	111.2	scaffold186.0	54,428
Pyb15_450	SNP	15	111.7	scaffold437.0	110,521
Pyb15_451	SNP	15	111.9	scaffold437.0	227,217

Pyb15_454	SNP	15	112.7	scaffold376.0	412,311
Pyb15_455	SNP	15	112.9	scaffold376.0	412,324
Pyb15_456	SNP	15	113.2	scaffold312.2	158,343
Pyb15_457	SNP	15	113.5	scaffold376.0	140,870
Pyb15_459	SNP	15	113.7	scaffold620.0	5,241
Pyb15_460	SNP	15	114.1	scaffold399.0	396,807
Pyd15_092	SNP	15	119.3	scaffold38.0	1,120,900
Pybd15_033	SNP	15	121.9	scaffold361.0	49,265
Pyb15_474	SNP	15	127.2	scaffold298.0	485,729
Pyb15_476	SNP	15	127.2	scaffold122.0	397,245
Pyb15_477	SNP	15	127.3	scaffold298.0	404,173
Pybd15_037	SNP	15	127.8	scaffold858.0	48,849
Pyb15_481	SNP	15	128.3	scaffold481.0	238,652
Pyb15_483	SNP	15	128.7	scaffold481.0	296,708
Pyb15_484	SNP	15	129.4	scaffold1131.0	32,650
Pyb15_485	SNP	15	129.4	scaffold569.0	69,837
Pyb15_486	SNP	15	129.4	scaffold1131.0	47,874
Pyb15_487	SNP	15	129.5	scaffold537.0	308,264
Pyb15_488	SNP	15	129.8	scaffold537.0	269,980
Pyb15_490	SNP	15	129.9	scaffold141.0	63,584
Pyb15_491	SNP	15	129.9	scaffold141.0	335,868
Pyb15_493	SNP	15	130.0	scaffold1131.0	76,983
Pyb15_496	SNP	15	131.2	scaffold186.0	41,626
Pyb15_497	SNP	15	132.5	scaffold437.0	107,302
Pyb15_505	SNP	15	138.2	scaffold108.0	339,364
Pyb15_507	SNP	15	139.0	scaffold742.0	94,253
Pyb15_508	SNP	15	139.3	scaffold306.0	84,083
Pyb15_509	SNP	15	139.3	scaffold149.0	340,181
Pyb15_510	SNP	15	139.3	scaffold306.0	40,297
Pyb15_514	SNP	15	139.8	scaffold149.0	624,096
Pyb15_518	SNP	15	140.0	scaffold149.0	154,478
Pyb15_522	SNP	15	140.2	scaffold108.0	201,426
Pyb15_523	SNP	15	140.2	scaffold380.0	101,080
Pyb15_525	SNP	15	140.3	scaffold108.0	102,362
Pyb15_528	SNP	15	140.4	scaffold149.0	446,086
Pyb15_532	SNP	15	140.5	scaffold178.0	486,932
Pyb15_536	SNP	15	141.2	scaffold178.0	450,346
Pyb15_541	SNP	15	141.5	scaffold178.0	504,659
Pyb15_545	SNP	15	141.9	scaffold178.0	328,719
Pyb15_546	SNP	15	141.9	scaffold178.0	446,353
Pyb15_547	SNP	15	142.3	scaffold776.0	17,590
Pyb15_548	SNP	15	142.7	scaffold9.0	397,735
Pyb15_549	SNP	15	143.1	scaffold9.0	1,356,648
Pyb15_551	SNP	15	143.5	scaffold141.0	560,524

Pyb15_557	SNP	15	144.0	scaffold141.0	485,994
Pybd15_042	SNP	15	148.3	scaffold9.0	278,897
Pyb15_565	SNP	15	150.2	scaffold100.0	455,771
Pybd15_044	SNP	15	153.9	scaffold108.0	324,130
Pybd15_046	SNP	15	158.7	scaffold742.0	157,659
Pyb15_567	SNP	15	158.9	scaffold742.0	44,041
Pyb15_568	SNP	15	159.0	scaffold306.0	90,697
Pyb15_569	SNP	15	159.0	scaffold149.0	743,003
Pyb15_570	SNP	15	159.0	scaffold108.0	169,555
Pyb15_571	SNP	15	159.0	scaffold742.0	106,933
Pyb15_572	SNP	15	159.5	scaffold756.0	205,828
Pyb15_581	SNP	15	160.9	scaffold306.0	53,163
Pyb15_585	SNP	15	161.9	scaffold178.0	344,987
Pyb15_586	SNP	15	161.9	scaffold9.0	356,083
Pyb15_587	SNP	15	162.0	scaffold9.0	1,245,729
Pyb15_588	SNP	15	162.4	scaffold9.0	1,258,935
Pyb15_589	SNP	15	162.4	scaffold9.0	294,160
Pyb15_590	SNP	15	162.5	scaffold9.0	1,417,132
Pyb15_591	SNP	15	162.5	scaffold9.0	1,258,984
Pyd15_109	SNP	15	162.6	scaffold178.0	417,812
Pyb15_592	SNP	15	162.7	scaffold9.0	514,207
Pyb15_598	SNP	15	163.8	scaffold141.0	379,794
Pyb15_602	SNP	15	164.1	scaffold141.0	485,961
Pyb15_603	SNP	15	164.2	scaffold141.0	549,353
Pyb15_608	SNP	15	164.8	scaffold141.0	454,650
Pyb15_615	SNP	15	165.7	scaffold55.0	693,507
Pyb15_617	SNP	15	166.0	scaffold55.0	850,102
Pyb15_618	SNP	15	166.0	scaffold55.0	938,146
Pyb15_622	SNP	15	166.7	scaffold55.0	701,721
CH02c09	SSR	15	166.8	scaffold100.0	96,299-96,541
Pyb15_625	SNP	15	170.5	scaffold100.0	482,667
Pyb15_626	SNP	15	170.5	scaffold100.0	443,562
Pyb15_627	SNP	15	170.6	scaffold100.0	776,453
NB102a	SSR	15	170.7	scaffold139.0	48,052-48,222
Pyb15_629	SNP	15	171.7	scaffold33.0	436,696
Pyb15_632	SNP	15	172.6	scaffold1074.0	114,548
Pyb15_634	SNP	15	172.7	scaffold965.0	57,854
Pyb15_636	SNP	15	172.8	scaffold1074.0	105,937
Pyb15_642	SNP	15	173.6	scaffold31.0	799,009
Pyb15_645	SNP	15	174.2	scaffold602.0	123,130
Pyb15_646	SNP	15	174.4	scaffold31.0	827,278
Pyb15_647	SNP	15	174.4	scaffold31.0	833,032
Pyb15_648	SNP	15	175.1	scaffold146.0	516,693
Pyb15_654	SNP	15	176.4	scaffold602.0	229,355

Pyb15_655	SNP	15	176.4	scaffold31.0	1,176,048
Pyb15_657	SNP	15	176.5	scaffold601.0	4,200
Pyb15_659	SNP	15	176.7	scaffold602.0	109,134
Pyb15_661	SNP	15	176.8	scaffold497.0	16,623
Pyd16_002	SNP	16	0.0	scaffold2.0	2,422,213
Pyd16_003	SNP	16	0.3	scaffold2.0	2,478,739
CN910353	SSR	16	11.2	scaffold488.0	315,599-315,035
Pyb16_007	SNP	16	11.9	scaffold488.0	207,357
Pyb16_014	SNP	16	14.5	scaffold1465.0	20,985
Pyb16_016	SNP	16	15.5	scaffold179.0	480,149
Pyd16_010	SNP	16	15.9	scaffold19.0	281,367
CH02d10a	SSR	16	16.1	scaffold19.0	1,020,644-1,020,242
Pyd16_016	SNP	16	17.2	scaffold19.0	1,237,850
Pyd16_017	SNP	16	17.7	scaffold2.0	3,484,424
Pyd16_018	SNP	16	17.7	scaffold19.0	425,943
Pyb16_025	SNP	16	18.4	scaffold885.0	134,031
Pyb16_028	SNP	16	19.2	scaffold885.0	44,665
Pyb16_029	SNP	16	19.2	scaffold885.0	44,693
Pybd16_003	SNP	16	20.6	scaffold78.0	253,805
Pyb16_044	SNP	16	23.7	scaffold1178.0	17,963
Pyb16_049	SNP	16	26.3	scaffold78.0	324,864
Pyb16_050	SNP	16	26.3	scaffold78.0	303,102
Pyb16_052	SNP	16	26.4	scaffold78.0	301,320
Pyb16_055	SNP	16	26.5	scaffold78.0	276,893
Pybd16_004	SNP	16	28.1	scaffold78.0	80,703
AU301431	SSR	16	28.1	scaffold224.0	59,512-59,713
Pyb16_058	SNP	16	28.4	scaffold78.0	426,466
Pyb16_059	SNP	16	28.7	scaffold78.0	387,512
Pyd16_028	SNP	16	28.8	scaffold71.0	1,074,866
Pyb16_060	SNP	16	29.9	scaffold78.0	438,420
Pyd16_030	SNP	16	31.3	scaffold78.0	528,123
Pyd16_035	SNP	16	34.5	scaffold885.0	37,347
CTG1062447	SSR	16	51.6	scaffold179.0	248,121-248,287
Pyb16_071	SNP	16	52.2	scaffold179.0	316,101
Pyb16_073	SNP	16	52.3	scaffold179.0	339,474
Pyb16_075	SNP	16	53.1	scaffold488.0	301,705
Pyb16_076	SNP	16	53.6	scaffold1553.0	20,662
Pyb16_078	SNP	16	55.0	scaffold488.0	207,324
Pybd16_005	SNP	16	55.3	scaffold60.0	90,108
Pyb16_084	SNP	16	57.2	scaffold218.0	497,865
Pyb16_085	SNP	16	57.6	scaffold284.0	403,074
Pyb16_088	SNP	16	58.6	scaffold284.0	522,906
Pyb16_091	SNP	16	59.1	scaffold184.0	174,758
Pyb16_092	SNP	16	59.2	scaffold184.0	238,723

Pyd16_047	SNP	16	60.4	scaffold185.0	434,482
Pyb16_100	SNP	16	60.5	scaffold184.0	238,679
Pyb16_101	SNP	16	60.6	scaffold184.0	215,935
Pyb16_105	SNP	16	61.5	scaffold306.0	24,660
Pyb16_106	SNP	16	61.9	scaffold1055.0	6,521
Pyb16_108	SNP	16	62.3	scaffold200.0	184,055
Pyb16_110	SNP	16	62.7	scaffold135.0	119,474
Pyb16_116	SNP	16	65.8	scaffold60.0	435,463
Pyb16_120	SNP	16	66.6	scaffold60.0	299,713
Pyb16_122	SNP	16	66.7	scaffold60.0	158,832
Pyb16_123	SNP	16	67.0	scaffold60.0	268,224
Pyd16_055	SNP	16	67.0	scaffold60.0	299,683
Pyb16_124	SNP	16	67.1	scaffold60.0	220,393
Pyb16_125	SNP	16	67.2	scaffold60.0	107,198
Pyb16_130	SNP	16	68.5	scaffold597.0	205,044
Pyb16_135	SNP	16	72.0	scaffold597.0	79,289
Pyd16_056	SNP	16	72.7	scaffold79.0	320,445
Pyb16_138	SNP	16	72.7	scaffold597.0	107,520
Pyb16_140	SNP	16	72.9	scaffold1133.0	28,732
Pyb16_142	SNP	16	73.1	scaffold1133.0	26,042
Pyb16_143	SNP	16	73.1	scaffold363.0	449,166
Pyb16_144	SNP	16	73.1	scaffold448.0	71,995
Pyb16_149	SNP	16	73.6	scaffold21.0	1,061,119
Pyb16_153	SNP	16	73.9	scaffold363.0	426,255
Pyb16_156	SNP	16	73.9	scaffold1496.0	9,507
Pyb16_160	SNP	16	74.0	scaffold79.0	252,937
Pyd16_058	SNP	16	74.2	scaffold79.0	315,964
Pyb16_164	SNP	16	74.3	scaffold79.0	516,431
Pyb16_168	SNP	16	75.0	scaffold555.0	184,259
Pyb16_170	SNP	16	76.1	scaffold796.0	9,338
Pyb16_171	SNP	16	76.2	scaffold392.0	102,845
Pyb16_172	SNP	16	76.3	scaffold68.0	1,092,790
Pyb16_176	SNP	16	77.4	scaffold77.0	128,404
Pyb16_180	SNP	16	77.8	scaffold77.0	283,109
Pyb16_182	SNP	16	78.5	scaffold77.0	712,004
Pyb16_184	SNP	16	79.3	scaffold187.0	619,376
Pyb16_186	SNP	16	79.6	scaffold219.0	465,599
Pyb16_187	SNP	16	79.8	scaffold187.0	372,394
Pyb16_189	SNP	16	79.8	scaffold187.0	313,661
Pyb16_190	SNP	16	80.0	scaffold187.0	620,977
Pyb16_192	SNP	16	80.1	scaffold187.0	679,740
Pyb16_193	SNP	16	80.3	scaffold219.0	499,150
Pyb16_198	SNP	16	80.7	scaffold187.0	621,029
Pyb16_201	SNP	16	81.4	scaffold130.0	676,592

Pyb16_203	SNP	16	81.4	scaffold130.0	453,261
Pyd16_069	SNP	16	98.2	scaffold184.0	121,741
Pyd16_073	SNP	16	103.8	scaffold179.0	295,915
Pyd16_075	SNP	16	106.2	scaffold179.0	272,383
Pyd16_081	SNP	16	107.4	scaffold928.0	104,820
Pyd16_083	SNP	16	107.4	scaffold77.0	638,837
Pyd16_093	SNP	16	115.5	scaffold555.0	238,804
NB123a	SSR	16	115.9	scaffold555.0	101,484-102,343
Pybd16_010	SNP	16	125.4	scaffold102.0	256,448
Pybd16_011	SNP	16	126.3	scaffold694.0	187,032
Pyb16_213	SNP	16	127.0	scaffold494.0	99,370
Pyb16_214	SNP	16	127.2	scaffold21.0	1,084,427
Pyb16_218	SNP	16	127.5	scaffold694.0	26,646
Pyb16_221	SNP	16	127.6	scaffold694.0	114,124
Pyb16_222	SNP	16	127.6	scaffold102.0	283,854
Pyb16_225	SNP	16	127.7	scaffold694.0	201,169
Pybd16_012	SNP	16	127.9	scaffold694.0	169,724
Pyb16_231	SNP	16	128.9	scaffold750.0	44,174
Pyb16_233	SNP	16	128.9	scaffold102.0	254,762
Pyb16_236	SNP	16	129.7	scaffold102.0	141,380
Pybd16_013	SNP	16	130.1	scaffold102.0	355,642
Pyb16_240	SNP	16	133.0	scaffold1167.0	15,557
Pyb16_242	SNP	16	133.2	scaffold79.0	559,038
Pybd16_014	SNP	16	134.6	scaffold750.0	153,796
Pybd16_015	SNP	16	137.1	scaffold694.0	87,898
Pybd17_001	SNP	17	0.0	scaffold192.0	527,600
NH014a	SSR	17	0.9	scaffold129.0	700,785-700,704
Pyb17_001	SNP	17	4.6	scaffold507.0	290,075
Pyb17_002	SNP	17	4.7	scaffold528.0	298,095
Pyb17_005	SNP	17	5.2	scaffold1096.0	72,741
Pyb17_006	SNP	17	5.4	scaffold31.0	1,082,355
Pyb17_009	SNP	17	5.7	scaffold192.0	608,438
Pyd17_003	SNP	17	5.8	scaffold635.0	244,289
Pyb17_012	SNP	17	6.6	scaffold129.0	553,294
Pyb17_016	SNP	17	7.2	scaffold129.0	655,560
Pyb17_030	SNP	17	9.6	scaffold34.0	291,746
Pyd17_004	SNP	17	9.9	scaffold501.0	18,930
Pyb17_034	SNP	17	10.2	scaffold34.0	298,127
Pyb17_035	SNP	17	10.3	scaffold34.0	314,174
Pyb17_037	SNP	17	10.3	scaffold658.0	20,750
Pyb17_044	SNP	17	11.0	scaffold34.0	398,959
Pyb17_045	SNP	17	11.1	scaffold34.0	1,030,265
Pyb17_047	SNP	17	11.2	scaffold34.0	943,698
Pyb17_048	SNP	17	11.2	scaffold34.0	1,004,380

Pyb17_049	SNP	17	11.3	scaffold34.0	339,417
Pyb17_055	SNP	17	11.6	scaffold34.0	413,997
Pyb17_057	SNP	17	11.8	scaffold34.0	979,931
Pyb17_059	SNP	17	11.9	scaffold851.0	63,417
Pybd17_005	SNP	17	12.3	scaffold658.0	216,488
Pyb17_065	SNP	17	12.7	scaffold749.0	41,668
Pyb17_066	SNP	17	12.7	scaffold851.0	22,577
Pyb17_068	SNP	17	13.1	scaffold194.0	18,299
Pybd17_006	SNP	17	14.4	scaffold194.0	101,187
Pyb17_082	SNP	17	15.0	scaffold194.0	255,762
Pyb17_083	SNP	17	15.0	scaffold194.0	288,110
Pyb17_085	SNP	17	15.6	scaffold194.0	346,140
Pyb17_086	SNP	17	15.8	scaffold194.0	271,252
Pyb17_087	SNP	17	16.1	scaffold153.0	287,806
Pyd17_012	SNP	17	16.3	scaffold194.0	306,894
Pybd17_007	SNP	17	16.6	scaffold631.0	96,495
Pyb17_089	SNP	17	17.0	scaffold611.0	144,841
Pyb17_090	SNP	17	17.1	scaffold611.0	133,452
Pyd17_013	SNP	17	17.1	scaffold348.0	269,837
Pyb17_091	SNP	17	17.1	scaffold611.0	23,890
Pyb17_093	SNP	17	17.4	scaffold153.0	230,359
Pyb17_094	SNP	17	17.4	scaffold153.0	127,665
Pyb17_095	SNP	17	17.6	scaffold153.0	99,662
Pyb17_096	SNP	17	17.7	scaffold611.0	161,231
Pyd17_018	SNP	17	25.6	scaffold192.0	566,848
Pyd17_020	SNP	17	28.3	scaffold34.0	245,222
Pyd17_022	SNP	17	31.2	scaffold34.0	737,102
Pyd17_024	SNP	17	31.7	scaffold631.0	107,646
Pyb17_098	SNP	17	32.6	scaffold85.0	454,780
Pyb17_100	SNP	17	33.0	scaffold385.0	40,608
Pyb17_102	SNP	17	33.3	scaffold194.0	18,235
Pyb17_103	SNP	17	33.4	scaffold385.0	205,990
Pyb17_106	SNP	17	33.6	scaffold385.0	179,489
Pyb17_107	SNP	17	33.9	scaffold348.0	373,687
Pyb17_109	SNP	17	35.0	scaffold34.0	502,824
NH008b	SSR	17	35.2	scaffold884.0	5,324-5,622
Pyb17_113	SNP	17	35.8	scaffold851.0	61,987
Pyb17_114	SNP	17	35.9	scaffold34.0	1,064,356
Pyb17_115	SNP	17	36.4	scaffold658.0	78,484
Pyb17_122	SNP	17	40.6	scaffold507.0	142,060
Pyb17_124	SNP	17	40.9	scaffold1096.0	83,628
Pyd17_026	SNP	17	41.2	scaffold1695.0	3,186
Pyd17_027	SNP	17	41.2	scaffold710.0	220,550
Pyb17_134	SNP	17	42.6	scaffold884.0	6,906

Pyb17_137	SNP	17	42.9	scaffold11.0	1,704,667
Pyb17_141	SNP	17	44.9	scaffold11.0	1,637,128
Pyb17_145	SNP	17	45.4	scaffold501.0	206,396
Pyb17_146	SNP	17	45.5	scaffold479.0	310,184
Pyb17_147	SNP	17	45.6	scaffold476.0	229,560
Pyb17_152	SNP	17	47.4	scaffold423.0	4,032
Pyb17_156	SNP	17	47.5	scaffold469.0	283,658
Pyb17_160	SNP	17	47.9	scaffold8.0	329,412
Pyd17_034	SNP	17	48.4	scaffold335.0	247,537
Pyb17_165	SNP	17	48.9	scaffold8.0	174,382
Pyb17_167	SNP	17	49.1	scaffold998.0	107,633
Pyb17_169	SNP	17	49.2	scaffold8.0	266,996
Pyb17_170	SNP	17	49.8	scaffold8.0	244,516
Pyb17_175	SNP	17	51.8	scaffold406.0	259,920
Pybd17_011	SNP	17	52.9	scaffold768.0	19,543
Pyb17_182	SNP	17	55.6	scaffold11.0	1,599,037
Pybd17_012	SNP	17	58.0	scaffold192.0	534,785
Pybd17_013	SNP	17	58.3	scaffold406.0	312,567
Pybd17_014	SNP	17	61.1	scaffold11.0	726,302
Pybd17_015	SNP	17	61.2	scaffold406.0	336,408
Pybd17_017	SNP	17	63.8	scaffold524.0	109,242
Pyd17_040	SNP	17	67.3	scaffold658.0	52,474
Pyd17_041	SNP	17	68.8	scaffold8.0	279,993
Pyb17_195	SNP	17	69.4	scaffold323.0	470,405
Pyd17_044	SNP	17	70.9	scaffold42.0	1,135,000
Pyb17_196	SNP	17	72.6	scaffold1026.0	62,570
Pyd17_045	SNP	17	75.0	scaffold1371.0	38,197
Pybd17_019	SNP	17	75.4	scaffold1026.0	92,510
Pybd17_020	SNP	17	80.1	scaffold61.0	1,137,255
Pyb17_206	SNP	17	80.9	scaffold335.0	225,017
Pybd17_021	SNP	17	81.4	scaffold335.0	45,292
Pyb17_207	SNP	17	81.8	scaffold1230.0	59,473
CH01h01	SSR	17	82.7	scaffold703.0	160,426-160,550
Pybd17_022	SNP	17	83.0	scaffold524.0	42,834
Pyb17_209	SNP	17	84.1	scaffold11.0	890,184
Pyb17_210	SNP	17	84.1	scaffold11.0	880,537
Pyd17_057	SNP	17	86.6	scaffold524.0	118,622
Pyb17_216	SNP	17	86.8	scaffold11.0	139,588
Pyb17_218	SNP	17	86.9	scaffold11.0	609,904
Pyd17_058	SNP	17	86.9	scaffold1026.0	62,626
Pyd17_062	SNP	17	88.4	scaffold703.0	207,111
Pyd17_063	SNP	17	88.4	scaffold1430.0	14,953
Pybd17_025	SNP	17	90.9	scaffold323.0	446,005
CH05g03	SSR	17	95.9	scaffold245.0	164,785-164,956

NB110a	SSR	17	99.0	Not_anchored	-
Pyb17_219	SNP	17	100.9	scaffold201.0	216,817
Pyb17_224	SNP	17	101.8	scaffold703.0	206,330
Pyb17_225	SNP	17	101.9	scaffold245.0	221,283
Pyb17_227	SNP	17	102.2	scaffold703.0	175,772
Pyb17_228	SNP	17	102.3	scaffold703.0	202,449
Pyb17_229	SNP	17	102.3	scaffold201.0	9,910
Pyb17_232	SNP	17	102.5	scaffold245.0	171,351
Pyb17_233	SNP	17	102.5	scaffold245.0	157,961
Pyb17_234	SNP	17	102.6	scaffold201.0	185,372
Pyb17_236	SNP	17	102.8	scaffold18.0	603,818
Pyb17_237	SNP	17	102.8	scaffold201.0	235,598
Pyb17_241	SNP	17	104.0	scaffold1348.0	38,174
Pyb17_246	SNP	17	104.7	scaffold245.0	484,642
Pyb17_247	SNP	17	104.7	scaffold1348.0	38,204
Pyb17_253	SNP	17	105.5	scaffold80.0	37,294
Pyb17_254	SNP	17	105.5	scaffold80.0	46,168
Pyb17_255	SNP	17	105.5	scaffold80.0	740,058
Pyb17_256	SNP	17	105.5	scaffold80.0	81,029
Pyb17_257	SNP	17	105.6	scaffold422.0	177,175
Pyb17_258	SNP	17	105.6	scaffold80.0	12,477
Pyb17_259	SNP	17	105.6	scaffold269.0	1,132
Pyb17_265	SNP	17	106.0	scaffold80.0	683,365
Pyb17_266	SNP	17	106.0	scaffold587.0	118,974
Pyb17_267	SNP	17	106.0	scaffold959.0	51,348
Pyb17_268	SNP	17	106.0	scaffold80.0	9,733
Pyb17_269	SNP	17	106.1	scaffold80.0	316,700
Pyb17_270	SNP	17	106.1	scaffold80.0	983,863
Pyb17_271	SNP	17	106.1	scaffold587.0	140,846
Pyb17_272	SNP	17	106.1	scaffold422.0	353,595
Pyb17_274	SNP	17	106.1	scaffold80.0	579,481
Pyb17_275	SNP	17	106.1	scaffold80.0	187,607
Pyb17_286	SNP	17	107.2	scaffold269.0	103,401
Pybd17_031	SNP	17	113.5	scaffold18.0	513,216
Pyb17_292	SNP	17	118.2	scaffold18.0	455,400
Pyb17_296	SNP	17	122.0	scaffold703.0	53,174
Pyb17_303	SNP	17	124.8	scaffold710.0	113,438
Pyb17_305	SNP	17	125.2	scaffold1695.0	4,884
Pyb17_306	SNP	17	125.2	scaffold88.0	738,248
Pyb17_315	SNP	17	127.9	scaffold954.0	120,283
AT000174	SSR	17	129.4	scaffold959.0	31,067-31,220
Pyb17_324	SNP	17	129.9	scaffold18.0	181,394
Pyb17_329	SNP	17	130.4	scaffold645.0	71,353
Pyb17_330	SNP	17	130.5	scaffold18.0	372,134

Pyb17_331	SNP	17	130.6	scaffold18.0	249,175
Pyb17_332	SNP	17	130.6	scaffold18.0	352,775
Pyb17_333	SNP	17	130.6	scaffold645.0	224,034
Pyb17_334	SNP	17	130.6	scaffold645.0	201,665
Pyb17_336	SNP	17	130.9	scaffold18.0	315,097
Pyb17_337	SNP	17	131.0	scaffold18.0	260,407
Pyb17_338	SNP	17	131.0	scaffold645.0	36,479
Pyb17_339	SNP	17	131.1	scaffold18.0	259,021
CH04c06	SSR	17	131.5	scaffold794.0	125,339-125,036
Pybd17_034	SNP	17	133.0	scaffold18.0	325,329
Pybd17_035	SNP	17	133.5	scaffold245.0	88,876
Pyb17_342	SNP	17	135.7	scaffold80.0	72,474
Pyb17_344	SNP	17	136.0	scaffold959.0	24,527
Pyb17_345	SNP	17	136.0	scaffold959.0	142,063
Pyb17_347	SNP	17	136.0	scaffold959.0	70,108
Pyb17_349	SNP	17	136.2	scaffold422.0	214,456
Pyb17_351	SNP	17	136.6	scaffold193.0	645,659
Pyb17_358	SNP	17	138.1	scaffold794.0	104,087
Pyb17_359	SNP	17	139.2	scaffold328.0	373,052
Pyb17_360	SNP	17	139.2	scaffold328.0	352,940
Pyb17_362	SNP	17	139.2	scaffold328.0	314,702
Pyb17_363	SNP	17	139.2	scaffold328.0	373,107
Pyb17_365	SNP	17	139.8	scaffold328.0	346,422
Pybd17_037	SNP	17	141.7	scaffold328.0	442,827
Pyb17_367	SNP	17	142.3	scaffold44.1	168,716
Pyb17_369	SNP	17	143.0	scaffold269.0	178,716
Pyb17_370	SNP	17	143.0	scaffold44.1	67,764
Pybd17_040	SNP	17	145.1	scaffold328.0	442,777
Pybd17_041	SNP	17	145.2	scaffold328.0	360,432
Pybd17_042	SNP	17	146.2	scaffold80.0	19,753
Pyd17_069	SNP	17	148.3	scaffold328.0	192,688
Pyd17_071	SNP	17	148.4	scaffold214.0	84,938
Pyd17_073	SNP	17	148.4	scaffold214.0	155,031

Supplemental figure lengend

Supplemental Figure S1. Figure of ‘Bayuehong’ x ‘Dangshansuli’ genetic map constructed with 3,241 markers (3,143 SNPs and 98 SSRs).

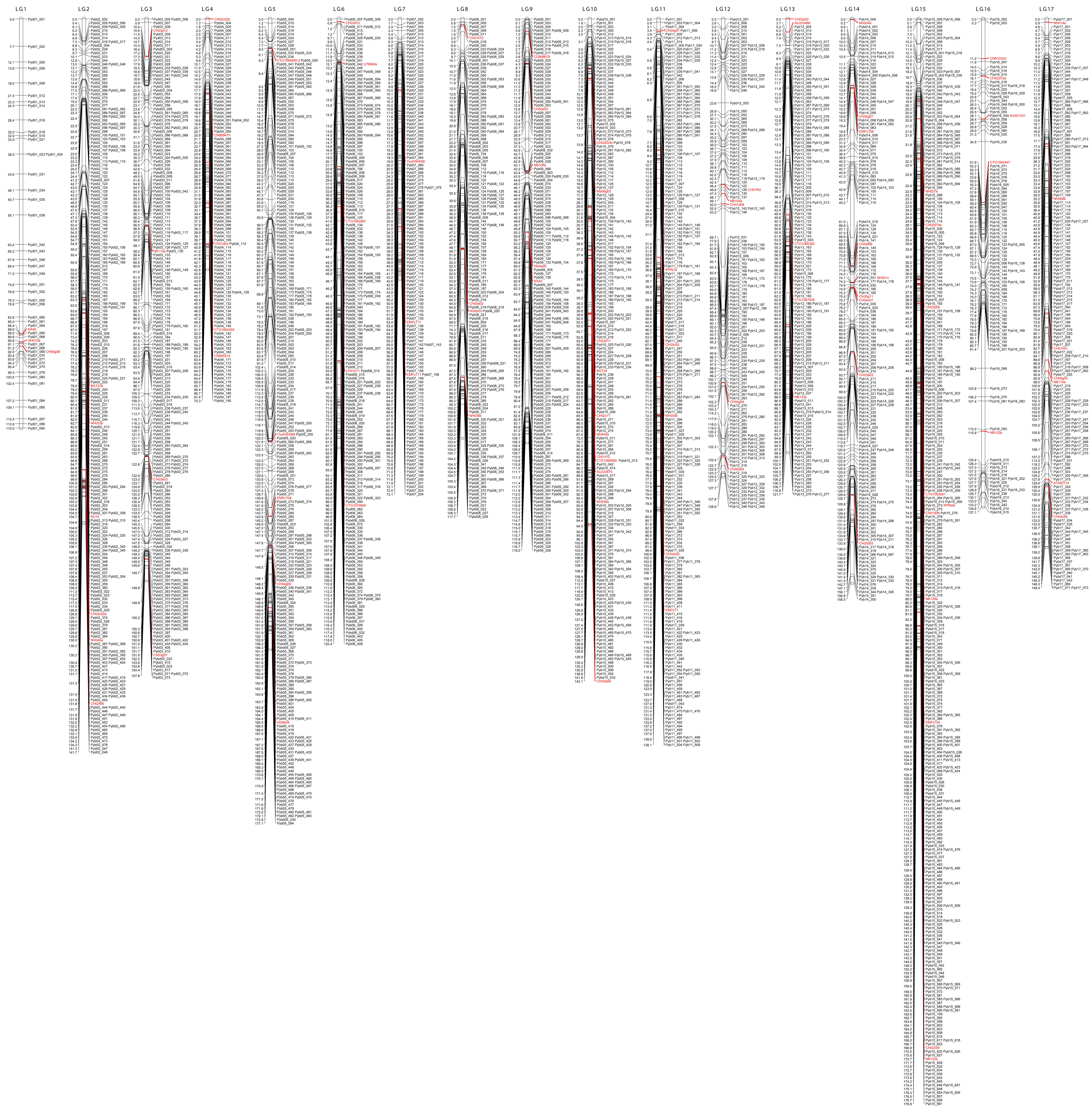
LG1
LG2
LG3
LG4
LG5
LG6
LG7
LG8
LG9
LG10
LG11
LG12
LG13
LG14
LG15
LG16
LG17
